# Ultrasound-induced gelation of a giant macrocycle†
†Electronic supplementary information (ESI) available: Detailed synthetic procedures, ^1^H and ^13^C NMR spectra of all compounds, experimental details for the gelation including a video, molecular modelling methods, single crystal and powder diffraction X-ray data, SEM/TEM experimental details. CCDC 1838654. For ESI and crystallographic data in CIF or other electronic format see DOI: 10.1039/c8cc04742a


**DOI:** 10.1039/c8cc04742a

**Published:** 2018-09-11

**Authors:** Diego Núñez-Villanueva, Michael A. Jinks, Jorge Gómez Magenti, Christopher A. Hunter

**Affiliations:** a Department of Chemistry , University of Cambridge , Lensfield Road , Cambridge CB2 1EW , UK . Email: dn325@cam.ac.uk

## Abstract

A 68-membered macrocycle undergoes ultrasound-induced supramolecular gelation in acetonitrile. The sonogel shows a remarkable thermostability, indicating that self-assembly is mediated by exceptionally robust non-covalent interactions. Model compounds indicate that the macrocyclic topology is essential for gelation to occur.

## Abstract 2

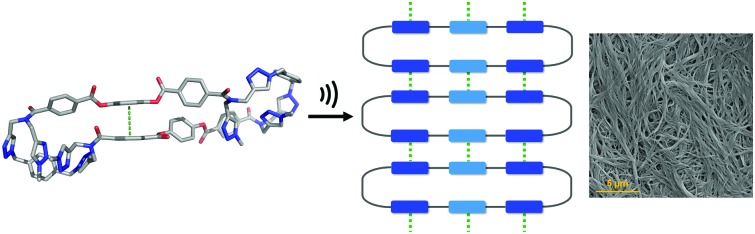
Supramolecular gelation of a 68-membered macrocycle triggered by sonication.

## 

Stimuli responsive materials have potential applications in biosensing, controlled release drug delivery, tissue engineering and catalysis.^1^ Supramolecular gels are particularly suitable to implement stimuli responsiveness because of the non-covalent nature of the fibrous gel networks that arise from the self-assembly of low-molecular weight gelators in a given solvent. Examples of supramolecular gels based on hydrogen bonding, metal–ligand coordination, aromatic interactions, van der Waals forces and hydrophobic effects have been described.^2^ External stimuli, such as temperature, pH, light, mechanical stress, solvent or additives, can induce gel–sol transitions by modulating the gel-forming interactions.^3^ The first example of a sol–gel transition triggered by sonication was reported by Naota and Koori.^4^ These sonogels have potential applications as wettability switches, hybrid materials, pollutant decontaminants or cell encapsulators.^5^

Ultrasound is commonly employed to break and disperse particles and supramolecular assemblies in the liquid state. Ultrasound produces high frequency mechanical waves that can create extreme physical and chemical conditions by the formation and collapse of bubbles under the pressure of the surrounding liquid.^6^ This cavitation effect can release enough kinetic energy to induce gelation by helping to overcome the activation barrier of kinetically disfavoured self-assembly pathways. Two different mechanisms of sonogelation have been proposed. At the molecular level, the activation of gelators can occur by inducing a conformational change, the transformation of intramolecular interactions into intermolecular interactions, or a rearrangement of labile bonds. At the supramolecular level, sonication can disrupt nucleation by fragmenting and dispersing aggregates, so that gelation occurs by the formation of entangled fibrillar structures from the independent propagation of separate assemblies.3e,6d,^7^ The chemical structures of reported sonogelators include organometallics, amino acids, peptides, ureas, cholesterols and heterocycles.^4^,^8^–^12^ Although organic macrocycles, such as calixarenes, porphyrins and cucurbiturils, can form supramolecular gels,^13^ macrocyclic sonogelators are rare. Some metallo-macrocycles have been reported, but only one example of an organic macrocyclic sonogelator is known.^4^,8c,^14^ Here, we describe the ultrasound-induced gelation of a new class of giant macrocycle (**1**, Fig. 1).

**Fig. 1:**
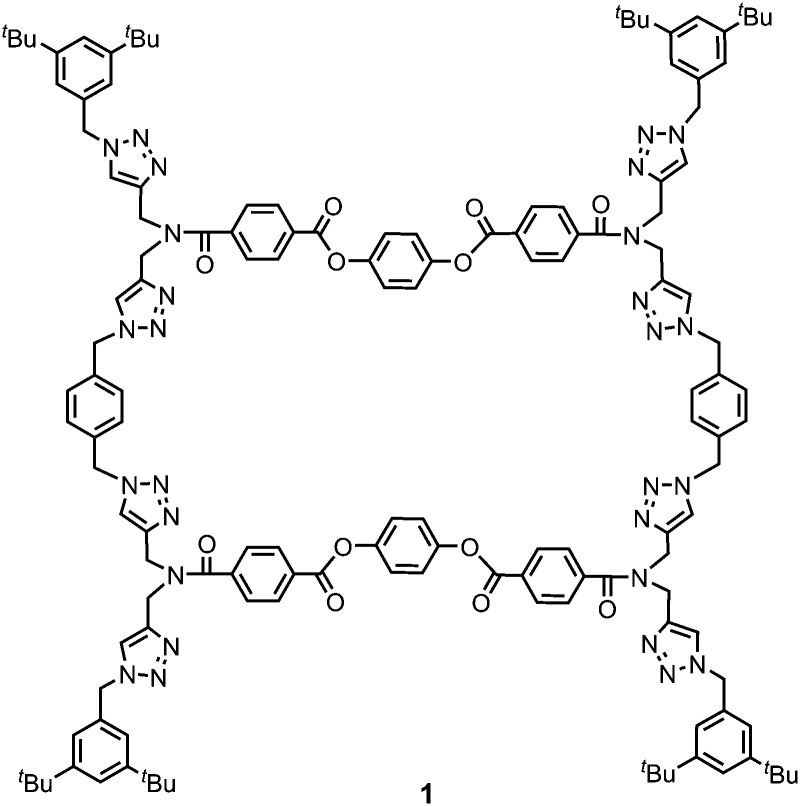
Chemical structure of gelator **1**.

Gelator **1** was synthesized in 9 steps from dipropargylamine and mono-methyl terephthalate (see ESI† for details). The compound was assembled using a series of sequential copper(i)-catalyzed azide–alkyne cycloaddition (CuAAC) and ester coupling reactions. The key macrocyclization step was achieved by double CuAAC reaction of a bis-propargyl derivative with 1,4-bis(azidomethyl)benzene under high dilution conditions. Control compounds **2** and **3** containing fragments of macrocycle **1** were also synthesised in order to obtain insights into the relationship between the chemical structure and the self-assembly properties (Fig. 2).

**Fig. 2:**
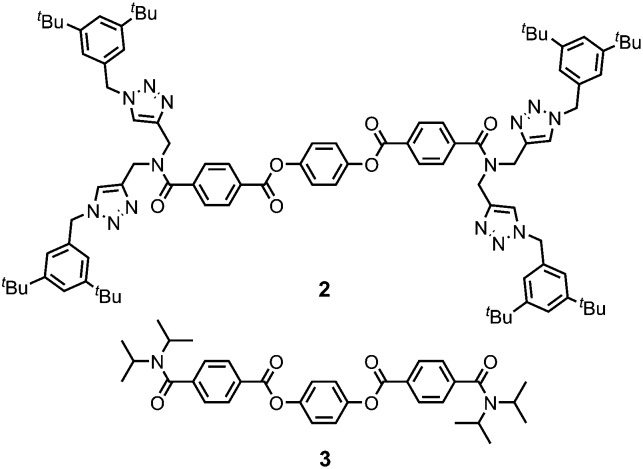
Chemical structure of control compounds **2** and **3**.

When a 14 mM solution of macrocycle **1** in CH_3_CN (4.3 wt%) was sonicated for 1 minute (45 kHz, 0.19 W cm^–2^), a white, opaque, and stable gel is formed. Fig. 3(a) shows the inverted test-tube test for gel formation. When this solution was not subjected to sonication, some turbidity could be observed after a period of hours, but a gel was not formed (Fig. S1a, ESI†). A 10-fold more dilute solution of **1** in CH_3_CN (1.4 mM, 0.4 wt%) formed a partial gel on sonication (Fig. S1b, ESI†). When this solution was not subjected to sonication, it remained clear for days with no signs of gelation. The critical gelation concentration (CGC) of **1** in CH_3_CN, the lowest concentration of the gelator which forms a stable gel, is 1.3–1.9 wt% (Fig. 3b). This value is typical for supramolecular gels formed by low-molecular weight gelators (0.1 to 10 wt%).^15^ Analysis of the material after ultrasound-induced gelation showed no evidence of any chemical change in composition, confirming the supramolecular nature of the gelation process (Fig. S2, ESI†).

**Fig. 3:**
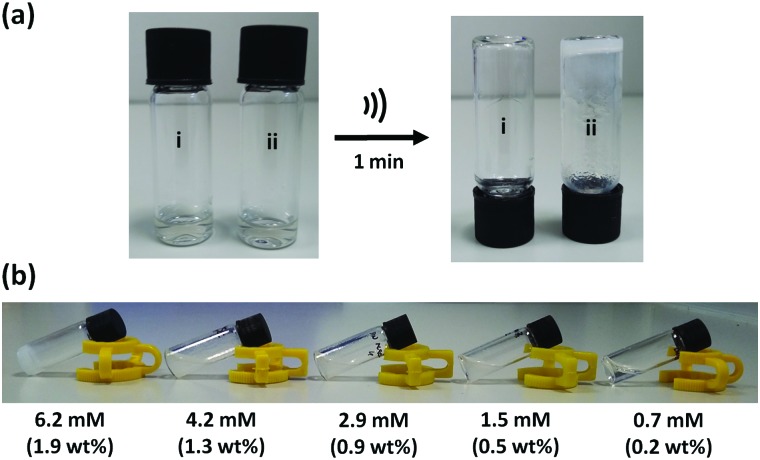
(a) Ultrasound-induced gelation of compound **1** in CH_3_CN at 14 mM. The left image shows two identical solutions of **1**. The right image corresponds to the sonication of solution (ii) for 1 min and solution (i) is the negative control. (b) Gel formed upon sonication of solutions of **1** in CH_3_CN at different concentrations.

The temperature-dependent behaviour of the gel was analysed in order to assess the strength of the non-covalent interactions that mediate the self-assembly of **1**. The dropping ball method can be used to estimate the gel–sol phase-transition temperature (*T*_gel_). For the sonogel of **1**, the temperature at which the ball started dipping into the gel was 105 °C (Fig. S5, ESI†).^16^ However, visual inspection of the sample at this temperature revealed that the gel was stable and had not undergone a gel–sol phase transition. Rather, shrinkage of the gel had occurred due to evaporation of solvent, because the temperature is much higher than the boiling point of CH_3_CN. Commonly, supramolecular gels exhibit a thermally reversible gel–sol phase transition, and only a few systems have been reported to show thermally triggered deswelling.^17^ The thermostability and unusual thermal shrinking properties of the gel indicate that remarkably robust non-covalent interactions govern the self-assembly of **1**. Only the addition of a competing solvent such as CHCl_3_ was found to disrupt the non-covalent network and promote dissociation of the gel (Fig. S1, ESI†).

Scanning electron microscopy (SEM) and transmission electron microscopy (TEM) were used to study the morphology of the sonogel formed by **1** at the macroscopic level (Fig. 4). SEM micrograph images of the xerogel prepared from the sonogel exhibit an intertwined 3D network consisting of twisted thin 1D nanofibrers. The fibres form bundles and intertwine to produce an entangled network. Similarly, the TEM images shows a web of fibres entangled together, with an average diameter of 100 ± 30 nm. X-ray powder diffraction was used to determine whether the ultrasound-induced aggregation of **1** proceeds with an increase in crystallinity.9*b* However, both the xerogel and the as-synthesized material show very broad X-ray diffraction peaks, which is typical of amorphous material, and a common feature of gels where disordered solvent is the major component (Fig. S7, ESI†).2*d*

**Fig. 4:**
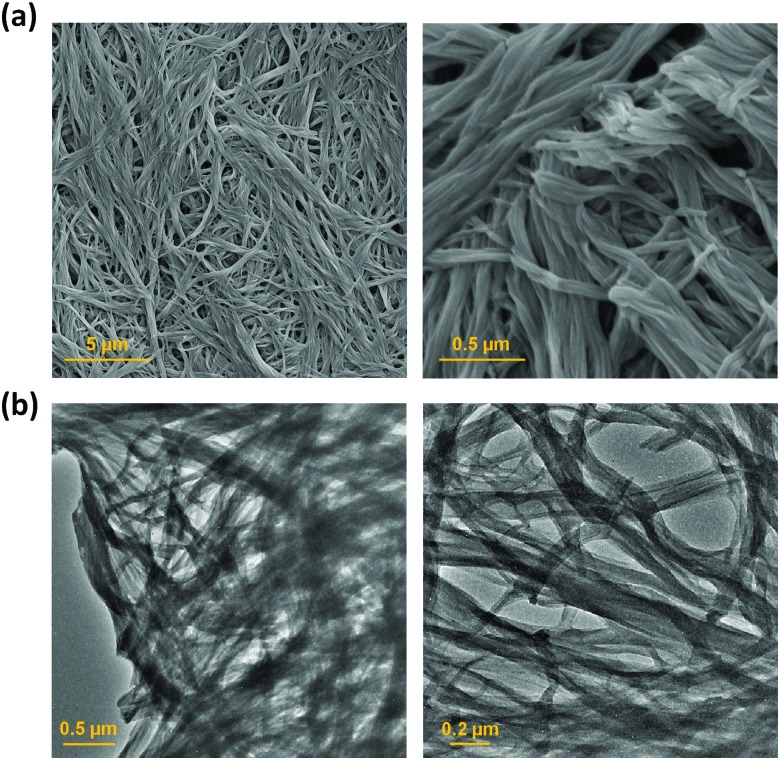
(a) SEM and (b) TEM micrographs of a xerogel formed by compound **1** at two magnifications.

Ultrasound-induced gelation experiments were carried out with CH_3_CN solutions of the acyclic analogues of **1**, compounds **2** and **3**. Solutions of these compounds were stable and remained clear without any sign of self-assembly after extensive sonication times (Fig. S4, ESI†). Similarly, no gel formation was observed for the acyclic precursor of **1** (compound **S16**, ESI†). These results suggest that the macrocyclic structure of **1** is the key to the observed gelation properties.

Macrocycle **1** has no strong H-bond donors, so H-bonding cannot be responsible for gelation, but the large number of aromatic rings suggests that aromatic interactions probably mediate the self-assembly process. Insight into the structural features of macrocycle **1** that might play a role was obtained from the X-ray structure of compound **3**, which is equivalent to the triaryl diester diamide fragment of the macrocycle (Fig. 5a). The crystal structure of **3** shows that the molecules assemble through offset stacking interactions between the hydroquinone units (Fig. 5b) and tilted edge-to-face aromatic interactions between the terephthalate units (Fig. 5c).

**Fig. 5:**
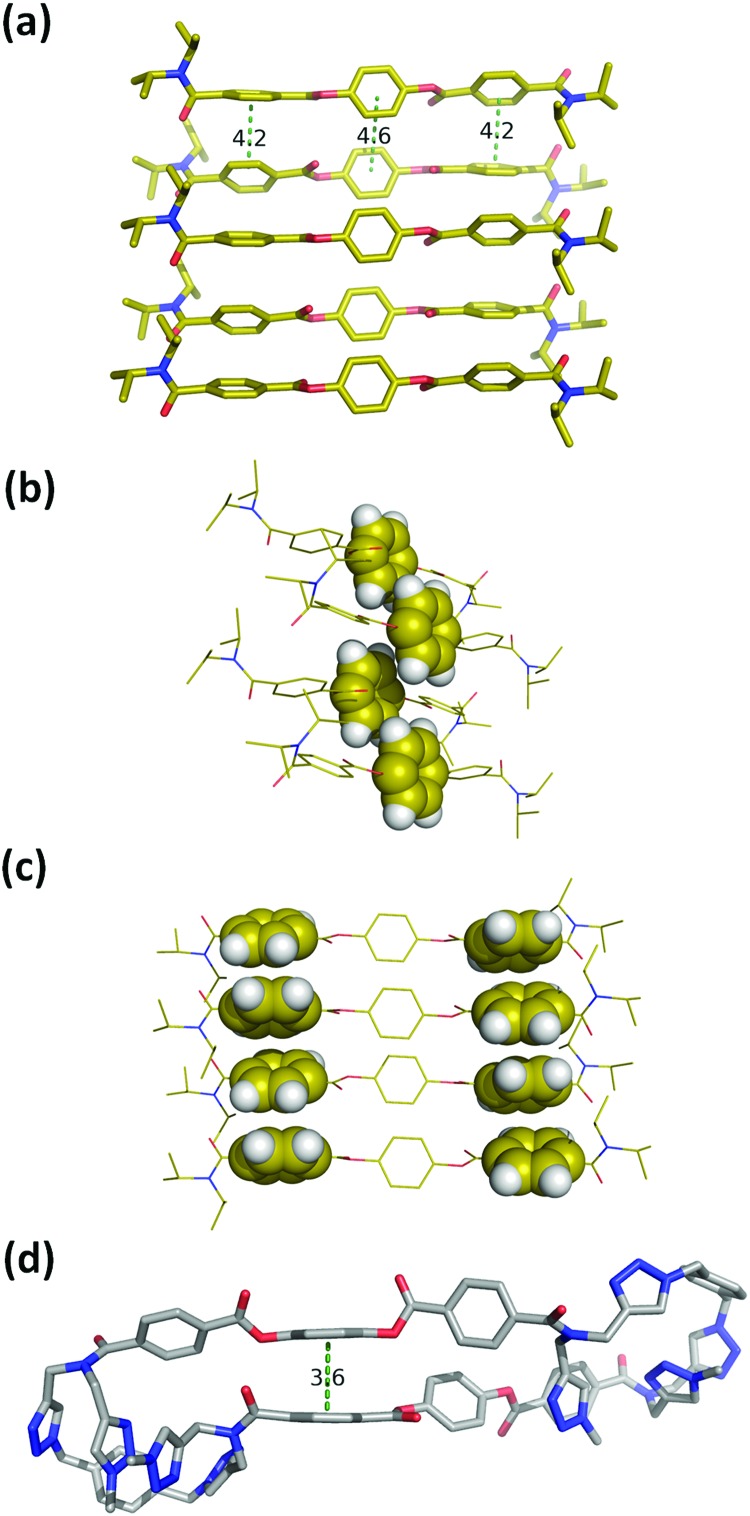
(a) Single crystal X-ray structure of **3**. Hydrogen atoms have been omitted for clarity.^18^ (b) View of the X-ray structure of **3** with the hydroquinone moiety highlighted in space-filling representation. (c) View of the X-ray structure of **3** with the terephthalate moieties highlighted in space-filling representation. (d) Lowest energy conformation from a conformational search of macrocycle **1** using molecular mechanics (OPLS3 force-field implemented in Macromodel, CHCl_3_ solvation).^19^,^20^ The external di-*t*-butylaryl groups were replaced by methyl groups to simplify the calculation. Hydrogen atoms have been omitted for clarity and the green dotted lines represent the aromatic interactions.

The fact that gel formation was not observed for compounds **2**, **3** and **S16** (Fig. S4, ESI†) suggests that the disposition of the aromatic groups in the macrocycle plays a pivotal role in the mechanism of self-assembly. Molecular mechanic calculations on macrocycle **1** suggest that there is an intramolecular aromatic stacking interaction between the hydroquinone and terephthalate moieties in the monomeric macrocycle (Fig. 5d). The ^1^H NMR spectrum of **1** is broad in CD_3_CN, consistent with intramolecular interactions that lead to slow conformational exchange processes on the NMR timescale. On dilution from 3.6 mM to 0.6 mM, no significant changes in the chemical shifts of the signals due to the aromatic protons were observed, indicating that the broadening is not due to self-association, which only takes place on sonication (Fig. S8, ESI†). Heating lead to changes in the ^1^H NMR spectrum: the signals due to the aromatic protons became less broad with small changes in chemical shift (Fig. S9, ESI†). In CDCl_3_, the solvent that dissociates the sonogel, the ^1^H NMR spectrum of **1** is sharper, and there are changes in the chemical shifts of the signals due to the aromatic protons compared with the spectra recorded in CD_3_CN (Fig. S8, ESI†). These results suggest that macrocycle **1** adopts a folded conformation in acetonitrile with intramolecular aromatic interactions that can be disrupted by heating or by a better solvent like chloroform.

One of the most common mechanisms for ultrasound-induced self-assembly involves the transformation of intramolecular interactions into intermolecular interactions, as the energy released by ultrasound waves helps to overcome the activation barrier of kinetically disfavoured self-assembly pathways.3e,^7^ Our hypothesis is that macrocycle **1** is kinetically trapped in a folded conformation due to the intramolecular aromatic interaction shown in Fig. 5(d). Ultrasonic irradiation induces a conformational change, breaking the intramolecular interaction and allowing self-assembly through the formation of an extended network of intermolecular aromatic interactions similar to those shown in Fig. 5(a). Fig. 6 illustrates how this mechanism could lead to fibrillar aggregates and give rise to the morphology observed by SEM and TEM.

**Fig. 6:**
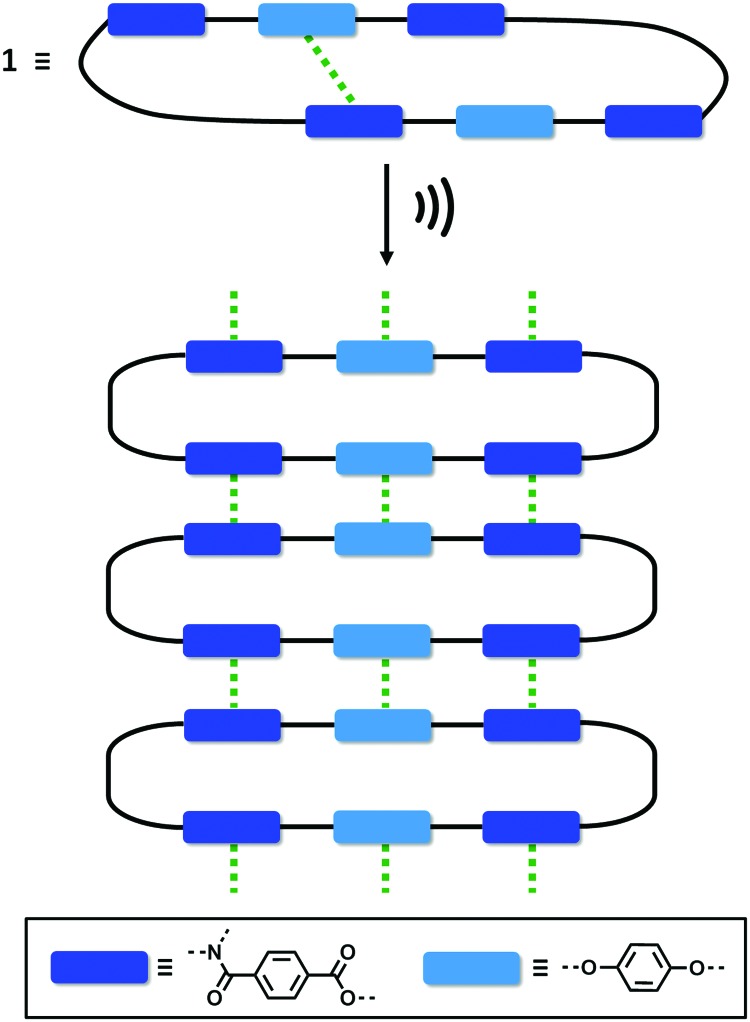
Proposed mechanism for the self-assembly of **1** upon ultrasound irradiation. The green dotted lines represent aromatic interactions.

In conclusion, we have synthesised a new type of giant macrocycle (68-membered ring) that undergoes ultrasound-induced supramolecular gelation. When dissolved in CH_3_CN, **1** self-assembles upon sonication generating a white, opaque and thermostable gel. This supramolecular gel not only shows a remarkable thermostability, but also exhibits thermally triggered shrinkage, which indicates that self-assembly is mediated by exceptionally robust non-covalent interactions. At the macroscopic level, the aggregates consist of 1D nanofibres that form bundles and intertwine to produce an entangled 3D network. The molecular structure of **1** suggests that aromatic interactions mediate the self-assembly process. The macrocyclic structure was found to be essential for sonogel formation: three acyclic analogues all failed to gelate under the same conditions. Molecular modelling, NMR spectroscopy and X-ray crystallography suggest that **1** is kinetically trapped in a folded conformation by weak intramolecular aromatic interactions. We suggest that when ultrasound is applied, the intramolecular interactions are disrupted allowing self-assembly to take place through intermolecular aromatic interactions.

This work was funded by Engineering and Physical Sciences Research Council (EP/J008044/2) and European Research Council (ERC-2012-AdG 320539-duplex). We thank Dr Andrew Bond for X-ray crystallographic data collection and analysis, Dr Heather Greer for electron microscopy studies, and Dr Ana M. Belenguer and Dr Giulio I. Lampronti for PXRD studies.

## Conflicts of interest

There are no conflicts to declare.

## Supplementary Material

Supplementary information

Supplementary movie

Crystal structure data
