# Supplementary material for: Ultrasound-induced gelation of a giant macrocycle
Source: Chem Commun (Camb). 2018 Sep 11;54(77):10874–7. doi: 10.1039/c8cc04742a (PMC6156880; doi:10.1039/c8cc04742a)
Supplement: Supplementary file 1 [file CC-054-C8CC04742A-s001.pdf]

Electronic Supplementary Material (ESI) for ChemComm.
This journal is © The Royal Society of Chemistry 2018

Supporting information

ULTRASOUND-INDUCED SELF-ASSEMBLY OF A GIANT MACROCYCLE

Diego Núñez-Villanueva,* Michael A. Jinks, Jorge Gómez Magenti and Christopher A. Hunter

Department of Chemistry, University of Cambridge, Lensfield Road, Cambridge CB2 1EW, UK. E-mail: dn325@cam.ac.uk

TABLE OF CONTENTS **Page**

Sonogel formation	S2
X-ray crystallography	S4
X-ray Powder Diffraction	S5
Molecular modelling calculations	S5
NMR dilution and variable temperature experiments of compound **1**	S6
NOESY and ROESY spectra of compound **1** in CD_3CN	S7
Transmission Electron Microscopy (TEM)	S8
Scanning Electron Microscopy (SEM)	S8
Synthesis and characterization of described compounds	S9
References	S67

Sonogel formation

Preparation of sonogels

CH_3CN was added to a vial containing compound **1**. The solution was filtered through cotton wool into a vial. A gel was formed upon sonication of the vial for 60 s (see supporting video) in a VWR USC100T ultrasonic bath (45 KHz, 0.19 W·cm^{-2}). The gel state was evaluated by the stable-to-inversion-of-a-test-tube method.

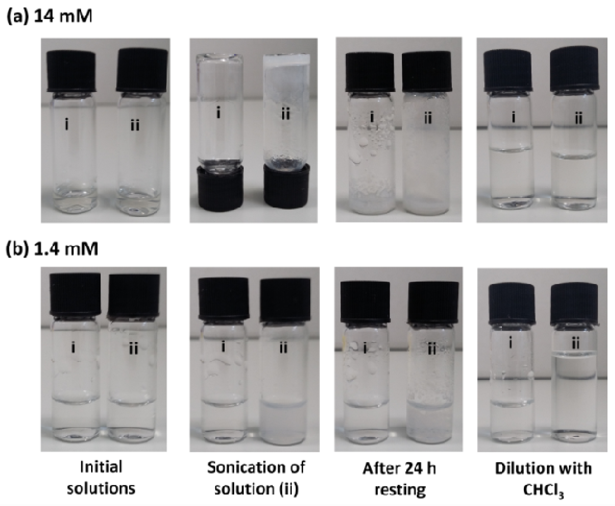

Figure S1. Ultrasound-induced gelation of compound **1** in CH_3CN at 14 mM (a) and 1.4 mM (b). From left to right, the images show two identical initial solutions of **1** (i and ii), the aspect after sonication of solution (ii) for 60 s while solution (i) is the negative control, the appearance after resting for 24 h is and the disassembly of the gel after dilution with $CHCl_3$.

Figure S2 shows the HPLC chromatogram of macrocycle **1** before and after the formation of the gel in order to show that there is no degradation or chemical transformation of **1** upon sonication and gel formation.

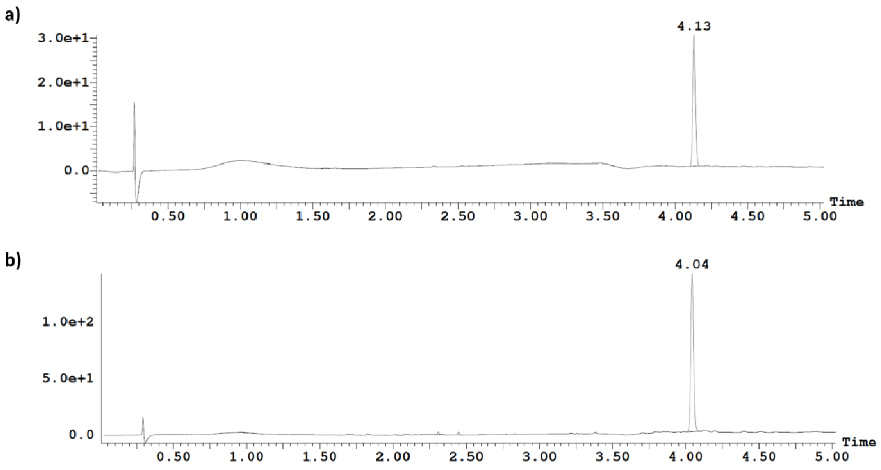

Figure S2. HPLC chromatogram of compound **1**: a) before formation of sonogel; b) after formation of the gel and dilution with $CHCl_3$. *Conditions*: UPLC CSH C18 Column, 2.1 x 50 mm, flow rate: 0.6 ml/min, gradient 5-95% B in 3 minutes + isocratic 95% B for 2 minutes (A: H_2O + 0.1% formic acid, B: acetonitrile + 0.1% formic acid).

Figure S3 shows the attempt to form a gel upon heating and cooling of a solution of **1** in CH_3CN. Formation of a gel is observed in the same solution after sonication.

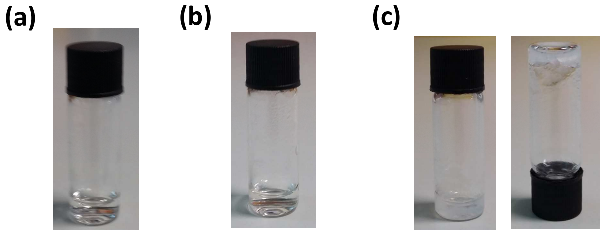

Figure S3. Attempts to form a gel upon heating and cooling of a solution of **1** in CH_3CN (2.8 mM). (a) and (b) Before and after heating at 60 ^{o}C for 1 h, respectively. (c) A gel is formed after sonication of the same solution for 1 min.

The same procedure for the gelation of compound **1** was applied to compounds **S16**, **2** and **3**. As shown in Figure S4, no gelation was observed in any case.

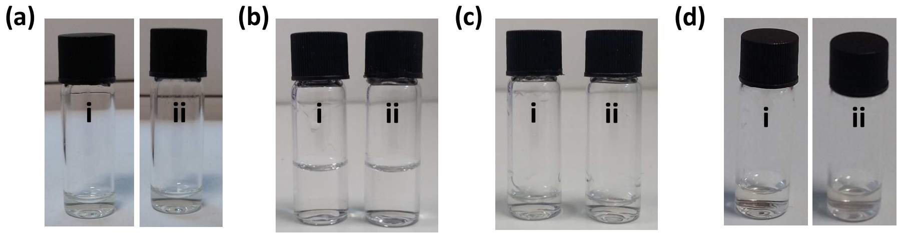

Figure S4. Attempts to form a gel upon sonication of solutions of compounds **S16**, **2** and **3**. (a) Solution (ii) is a solution of **S16** (9 mM) in CH_3CN after being sonicated for 5 minutes while solution (i) is the same solution before sonication. (b) Solution (ii) is a solution of **2** (1.4 mM) in CH_3CN after being sonicated for 5 minutes while solution (i) is the negative control. (c) Solution (ii) is a solution of **2** (14 mM) in CH_3CN after being sonicated for 5 minutes while solution (i) is the negative control. (d) Solution (ii) is a solution of **3** (12 mM) in CH_3CN after being sonicated for 5 minutes while solution (i) is the same solution before sonication.

Gel thermostability

T_{gel} was determined using the "dropping ball method".[S1] A stainless steel ball (63 mg, 2.5 mm in diameter) was placed on the surface of the gel, prepared in a glass tube as described in the text. The sample was placed in an oil bath and heated in steps of 5 ^{o}C from 40 ^{o}C until the ball started dipping into the gel (Figure S5).

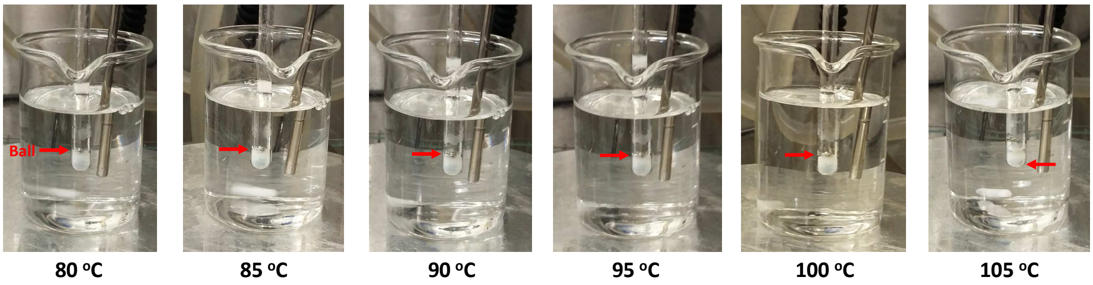

Figure S5. Determination of T_{gel} using the "dropping ball method"

X-ray crystallography

X-ray structure of compound 3 (CCDC 1838654)

Pure compound **3** (3 mg) was dissolved in $CHCl_3$ (1 mL), and the mixture was filtered to a vial and sealed with a plastic cap, resulting in crystallization after 5 days at room temperature. Crystals suitable for X-ray crystallography were selected using an optical microscope and examined at 180 K on a Nonius KappaCCD diffractometer using Mo Kα radiation (λ = 0.7107 Å). All non-hydrogen atoms were refined anisotropically. Hydrogen atoms were placed in idealized position.

**Formula**	$C_{34}H_{40}N_2O_6$		
**Temperature / K**	180		
**Space Group**	P 2_1/n		
**Cell Lengths/ Å**	**a** 7.4062 (2)	**b** 15.5550 (5)	**c** 27.3276 (9)
**Cell Angles/ °**	**α** 90	**β** 96.466(2)	**γ** 90
**Cell Volume/ Å^3**	3128.21		
**Z**	4		
**R factor**	0.039		

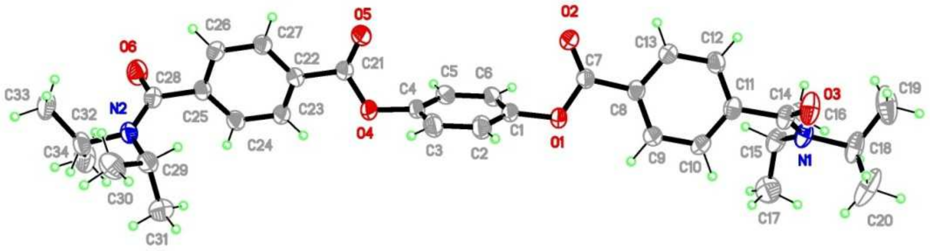

Figure S6. X-ray structure of derivative **3** in ORTEP view (ellipsoids are drawn at 50% probability level).

X-ray Powder Diffraction

Powder X-ray diffraction (PXRD) was performed at room temperature on a Panalytical Empyrean diffractometer emitting Cu Kα (1.540598 Å + 1.544426 Å) radiation. X-ray powder diffractogram was recorded in the 2θ range 3-45° (step size 0.02°, time/step 200 s).

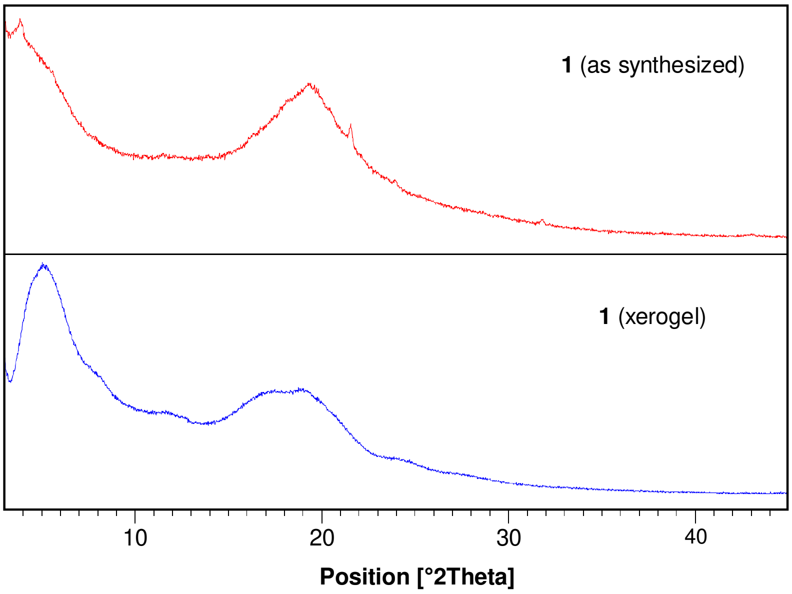

Figure S7. X-ray diffraction patterns of the as-synthesized compound **1** (top) and of the xerogel obtained after sonication of a solution of **1** in CH_3CN and deposition on the sample holder.

Molecular modelling calculations

Molecular mechanics calculations were performed using MacroModel implemented in Maestro 11 (Schrödinger release 2016-4).[S2] The structure of macrocycle **1** was simplified by replacing the 3,5-di-*tert*-butylbenzyl capping groups by methyl groups to save computational time. The structure was minimized first and the minimized structure was then used as the starting molecular structure for the MacroModel conformational search. The conformational search was performed twice from a different starting conformation. The force field used was OPLS3 as implemented in this software ($CHCl_3$ solvation). The charges were defined by the force field library and no cut off was used for non-covalent interactions. A Polak-Ribiere Conjugate Gradient (PRCG) was used and each minimisation was subjected to 10.000 iterations. The minima converged on a gradient with a threshold of 0.01. Conformational searches were performed from previously minimized structures using 100 steps per rotatable bond (maximum number of steps of 10.000). Images were created using PyMol.[S3]

NMR dilution and variable temperature experiments of compound 1

^{1}H NMR dilution experiment of compound **1** in CD_3CN was performed in a Bruker 500 MHz Avance TCI Cryoprobe spectrometer at 298 K. A 3.6 mM solution of **1** in CD_3CN was diluted by addition of neat CD_3CN and the NMR recorded at each concentration showed in Figure S8.

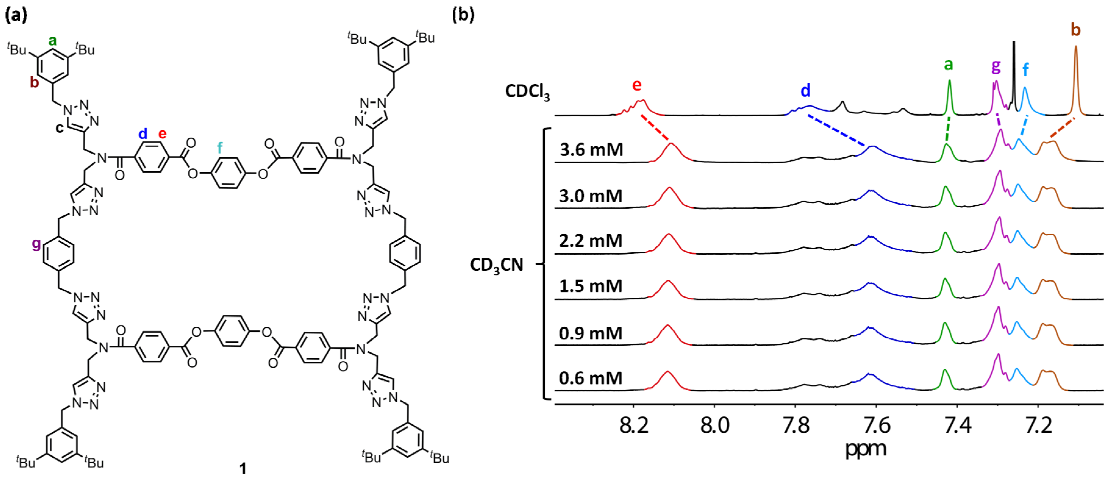

Figure S8. (a) Chemical structure of gelator **1** with aromatic protons labelled. (b) Partial 500 MHz ^{1}H NMR of **1** at different concentrations in CD_3CN and in $CDCl_3$ at 298 K.

Variable temperature NMR experiment of compound **1** (3.3 mM sample in CD_3CN) was performed in a Bruker 500 MHz AVIII HD Smart Probe spectrometer equipped with a BCU Chiller unit. The temperature of the sample was changed using the internal thermostat of the NMR spectrometer, and the sample was allowed to equilibrate in the probe until the probe thermometer gave a stable temperature (Figure S9).

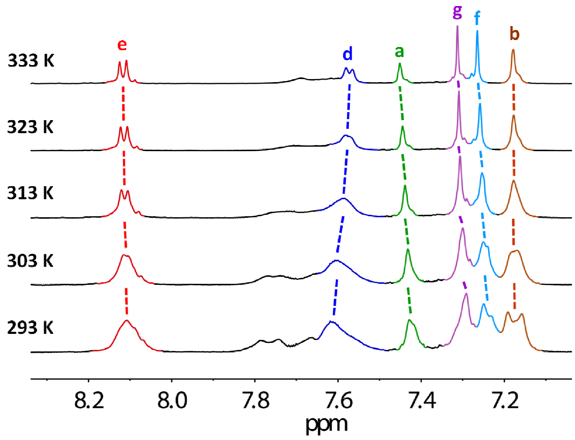

Figure S9. Variable temperature 500 MHz partial ^{1}H NMR of **1** at 3.3 mM in CD_3CN. Aromatic protons are labelled according to Fig. S8(a).

NOESY and ROESY spectra of compound 1 in CD_3CN

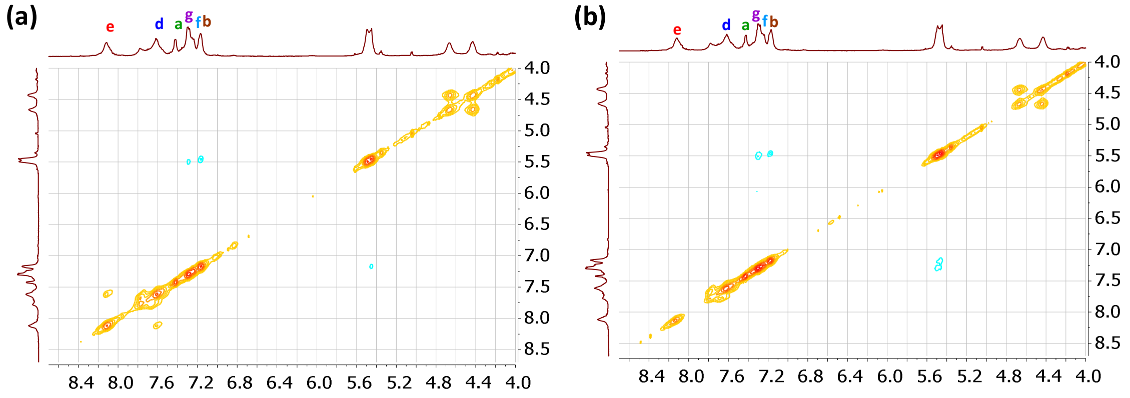

Figure S10. NOESY (a) and ROESY (b) spectra of **1** in CD_3CN. Aromatic protons are labelled according to Fig. S8(a).

Transmission Electron Microscopy (TEM)

The morphology of the gel was studied by using TEM at room temperature (25 °C). A 5 mM solution of the gel was prepared following the procedure described above. 4 µL of this solution was placed on a TEM grid (300 mesh size Cu grid) coated with a holey carbon film. The grid was allowed to dry by slow evaporation in air for 30 minutes and then under high vacuum for 4 hours. TEM images were recorded using a JEOL JEM-3010 electron microscope fitted with a LaB6 filament operating at 250kV. Images were recorded using a Gatan 794 CCD camera.

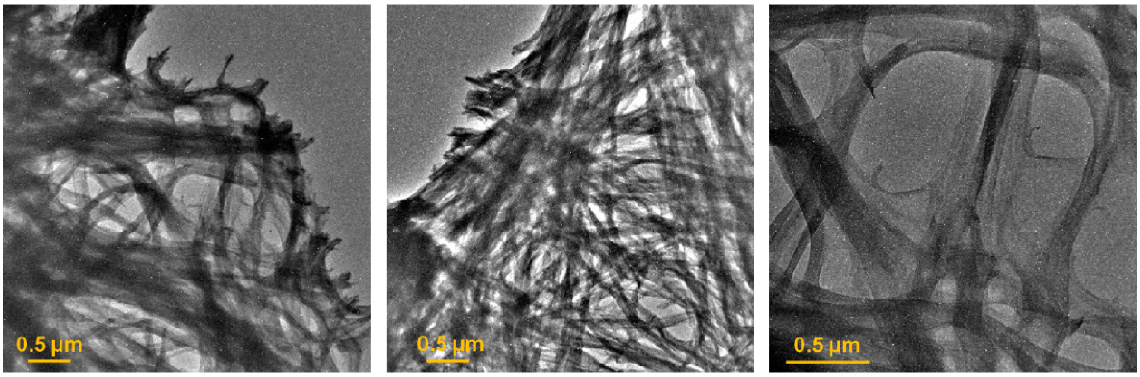

Figure S11. Additional TEM micrographs of a xerogel formed by compound **1** at different magnifications.

Scanning Electron Microscopy (SEM)

The same TEM grid was coated with 10nm Pt using a Quorum Technologies Q150T ES coater prior to SEM characterisation. Images were taken using a TESCAN MIRA3 FEG-SEM operating at 5kV.

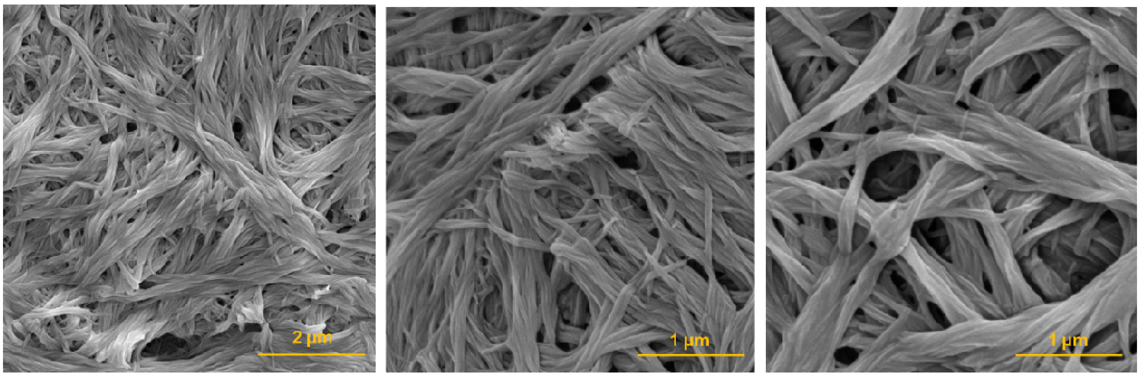

Figure S12. Additional SEM micrographs of a xerogel formed by compound **1** at different magnifications.

.

Synthesis and characterization of described compounds

Synthesis

The synthesis of gelator **1** involved the preparation of dipropargylamide building blocks **S2** and **S5** (Scheme 1). Amide coupling of dipropargylamine and mono-methyl terephthalate using 1-ethyl-3-(3-dimethylaminopropyl)carbodiimide (EDC) gave **S2** in quantitative yield. Basic hydrolysis of **S2** followed by ester coupling of carboxylic acid **S3** with monoprotected hydroquinone **S4** gave access to **S5** in good yield.

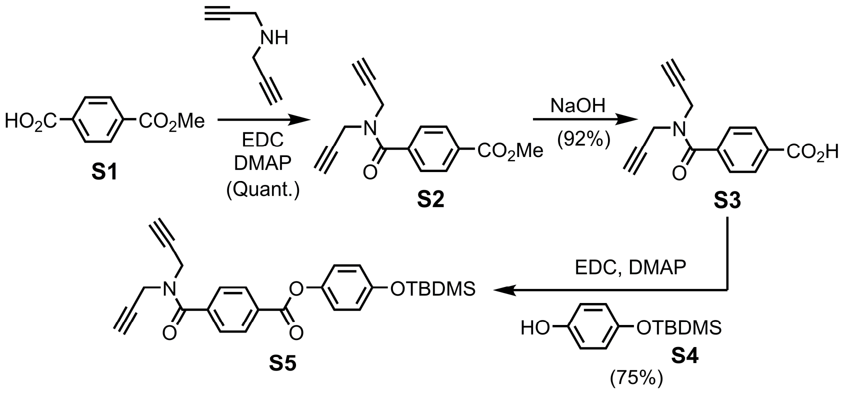

Scheme S1. Synthesis of building blocks **S2** and **S5**.

As shown in Scheme S2, the synthesis of the dicarboxylic acid **S10** started with the copper(I)-catalyzed azide-alkyne cycloaddition (CuAAC) reaction of **S2** with an excess of 1-(azidomethyl)-3,5-di-*tert*-butylbenzene **S6** to give a mixture compounds **S7** and **S8** that were separated by chromatography. CuAAC reaction of compound **S7** with 1,4-bis(azidomethyl)benzene **S9** followed by basic hydrolysis of the methyl ester afforded **S10** in excellent yield. Hydrolysis of **S8** gave the corresponding monocarboxylic acid **S11** in good yield. Following a similar strategy, compound **S5** was reacted with azide **S6** to give **S12** and **S13**, which were separated by chromatography (Scheme S3).

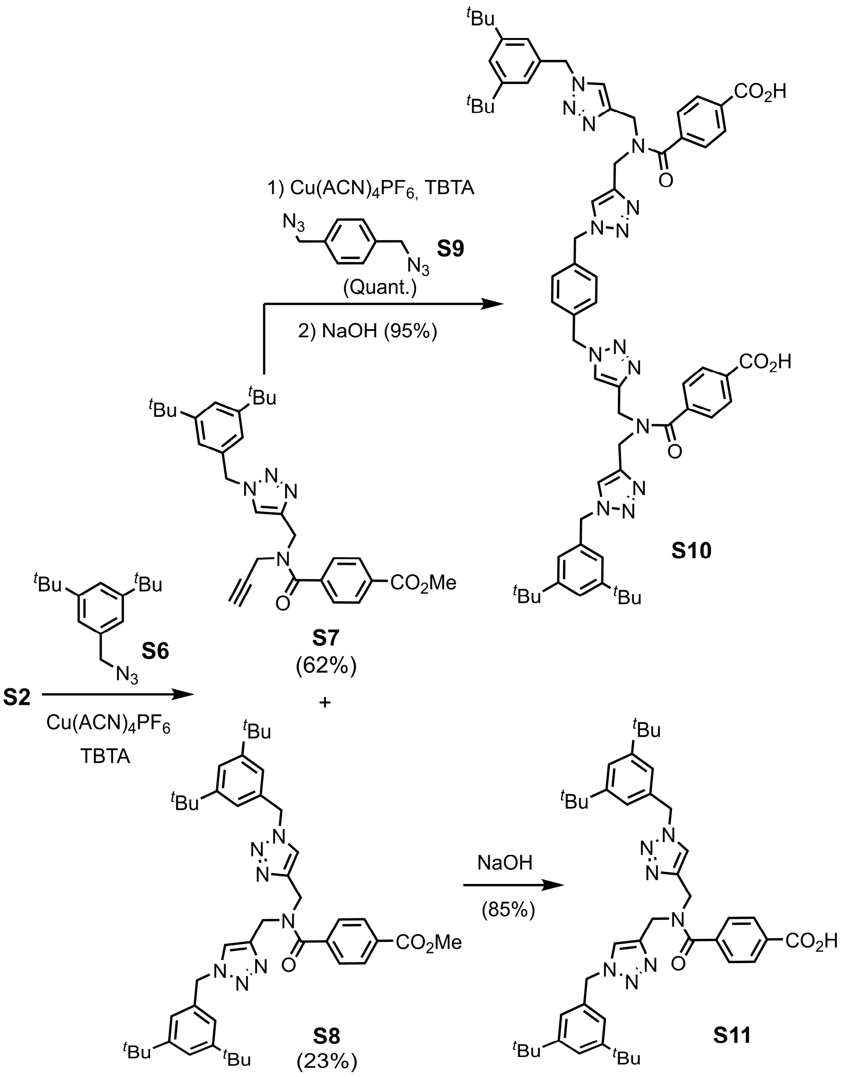

Scheme 2. Synthesis of carboxylic acids **S10** and **S11**.

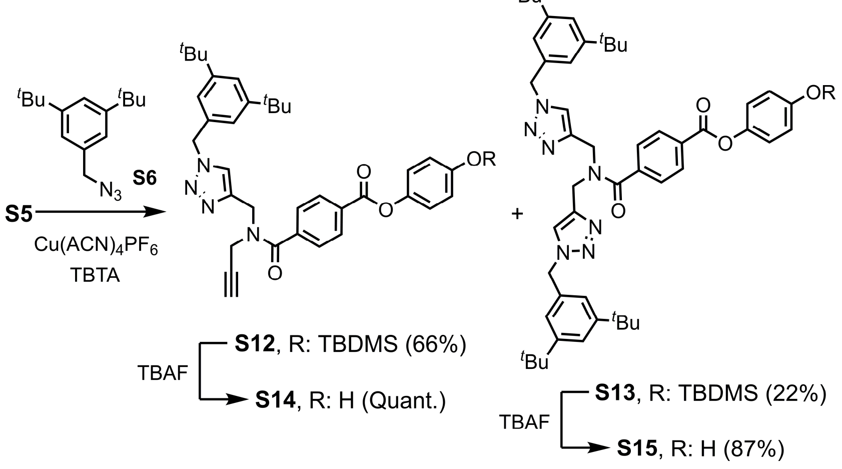

Scheme S3. Synthesis of phenol derivatives **S14** and **S15**.

Removal of the silyl protecting groups yielded phenols **S14** and **S15**. Ester coupling of the carboxylic acid dimer **S10** with **S14** gave compound **S16** in good yield (Scheme S4), and CuAAC macrocyclisation of **S16** with one equivalent of diazide **S9** under high dilution conditions afforded gelator **1**.

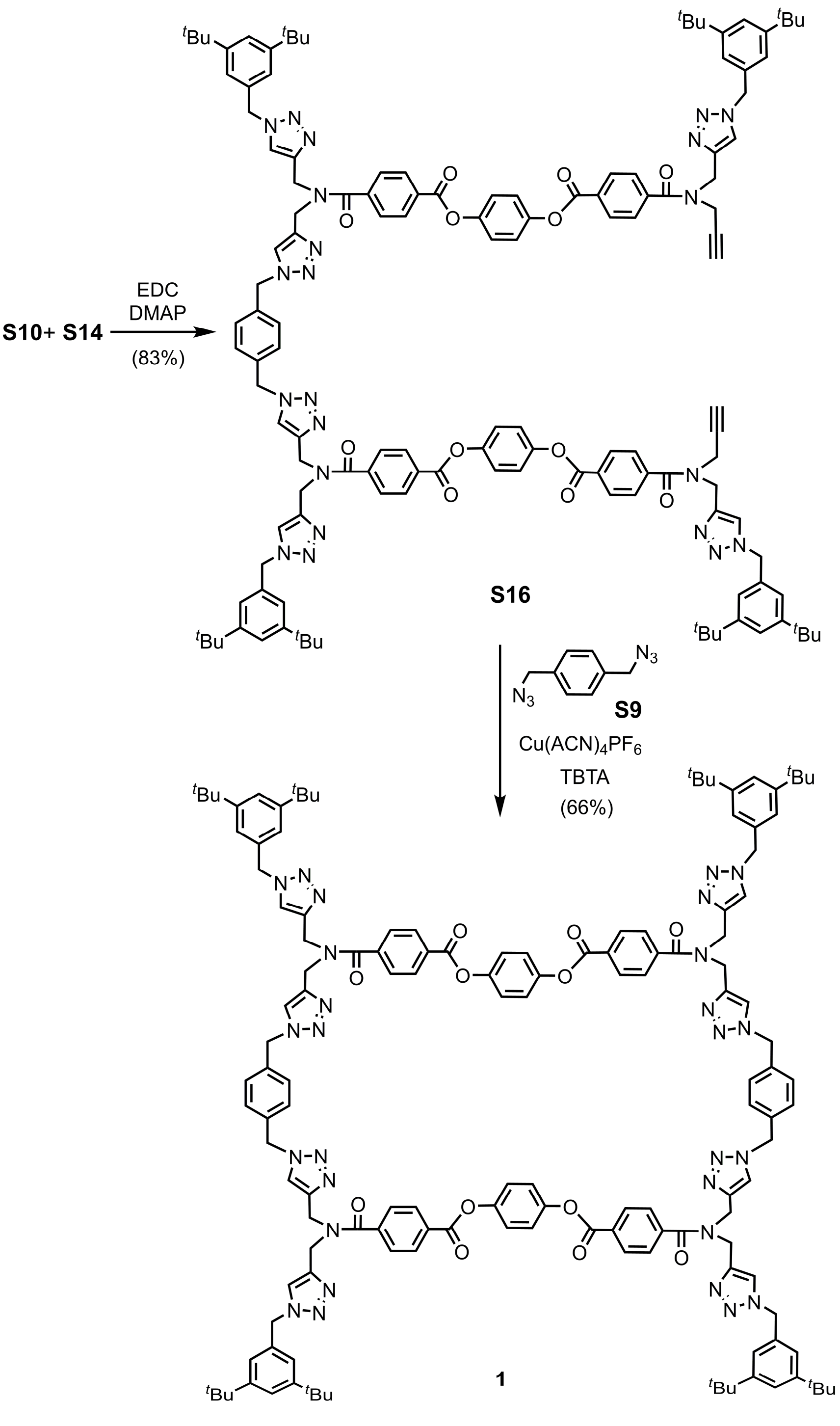

Scheme S4. Synthesis of macrocycle **1**.

Control compounds containing fragments of macrocycle **1** were synthesised in order to obtain insights into the relationship between the chemical structure and the self-assembly

properties. Ester coupling of **S11** with **S15** afforded **2** (Scheme S5), which is an acyclic analogue that contains most of the functional groups present in **1**. The diisopropylamide derivative **3** is another acyclic analogue that contains only the diester diamide moiety of **1**. The synthesis of **3** is shown in Scheme S6. Amide coupling of **S17** with diisopropylamine gave diisopropylamide **S18** in quantitative yield. Basic hydrolysis of **S18** followed by ester coupling with hydroquinone afforded **3**.

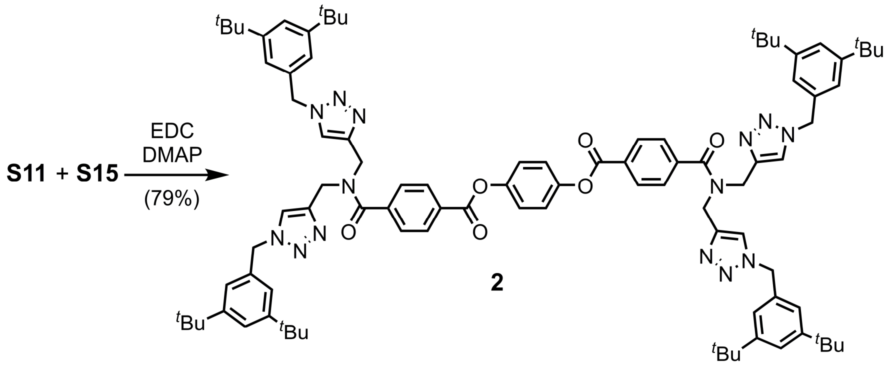

Scheme S5. Synthesis of **2**.

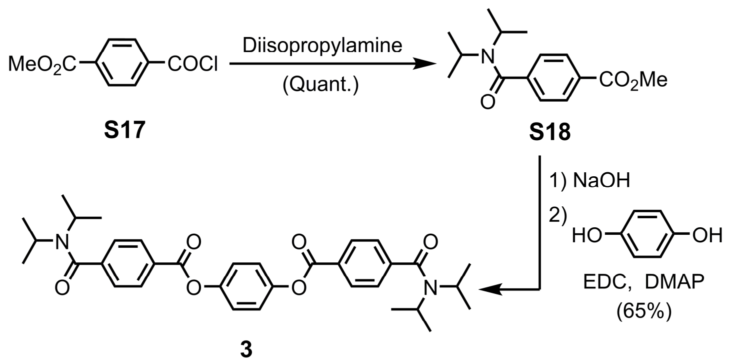

Scheme S6. Synthesis of **3**.

General experimental details

All the reagents and materials used in the synthesis of the compounds described below were bought from commercial sources, without prior purification. Thin layer chromatography was carried out using with silica gel 60F (Merck) on glass plates. Flash chromatography was carried out on an automated system (Combiflash Rf+ or Combiflash Rf Lumen) using prepacked cartridges of silica (25µ PuriFlash® columns). All NMR spectroscopy was carried out on a DPX400 or AVIII400 spectrometer using the residual solvent as the internal standard. All chemical shifts (δ) are quoted in ppm and coupling constants given in Hz. Splitting patterns are given as follows: s (singlet), bs (broad singlet), d (doublet), t (triplet), q (quadruplet), m (multiplet). FT-IR spectra were measured on a PerkinElmer Spectrum One spectrometer equipped with an ATR cell. Melting points were measured in a Mettler Toledo MP50 Melting Point System. ES+ was carried out on a Waters Xevo G2-S bench top QTOF machine.

Compounds **S1** and **S17** are commercially available. Compounds **S6**[S4] and **S9**[S5] were prepared according described procedures.

Methyl 4-(di(prop-2-yn-1-yl)carbamoyl)benzoate (S2).

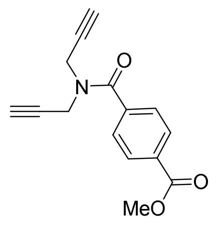

Mono-methyl terephthalate **S1** (0.977 g, 5.42 mmol), EDC (1.235 g, 6.44 mmol) and DMAP (0.065 g, 0.54 mmol) were dissolved in dry CH_2Cl_2 (10 ml). Dipropargylamine (0.500 g, 5.37 mmol) was added under N_2 atmosphere and the solution was left stirring at room temperature for 1h. The crude was diluted with EtOAc (20 mL) and washed with 5% aq. soln. HCl (3x), H_2O (1x) and brine. The organic phase was dried with $MgSO_4$ and concentrated *in vacuo* to yield **S2** as a light yellow solid (1.47 g, quantitative).

Melting point: 90-92 ^{o}C.

^{1}H NMR (400 MHz, CDCl$_3$): δ_H = 8.11 (d, 2H, J = 8.5 Hz), 7.62 (d, 2H, J = 8.5 Hz), 4.49 and 4.12 (bs, 4H, rotamers), 3.94 (s, 3H), 2.33 (s, 2H).

^{13}C NMR (100.6 MHz, CDCl$_3$): δ_C = 170.0, 166.4, 138.9, 132.0, 130.0, 127.3, 77.8, 73.7 and 72.8 (rotamers), 52.5, 38.4 and 33.8 (rotamers).

HRMS (ES+): calcd for $C_{15}H_{14}NO_3$ 256.0974 $[M+H]^+$, found 256.0974 $[M+H]^+$.

FT-IR (ATR): ν_{max} 3286, 1723, 1647, 1435, 1280, 1252 and 1111 cm^{-1}.

^{1}H-NMR (400 MHz, $CDCl_3$) Methyl 4-(di(prop-2-yn-1-yl)carbamoyl)benzoate (S2).

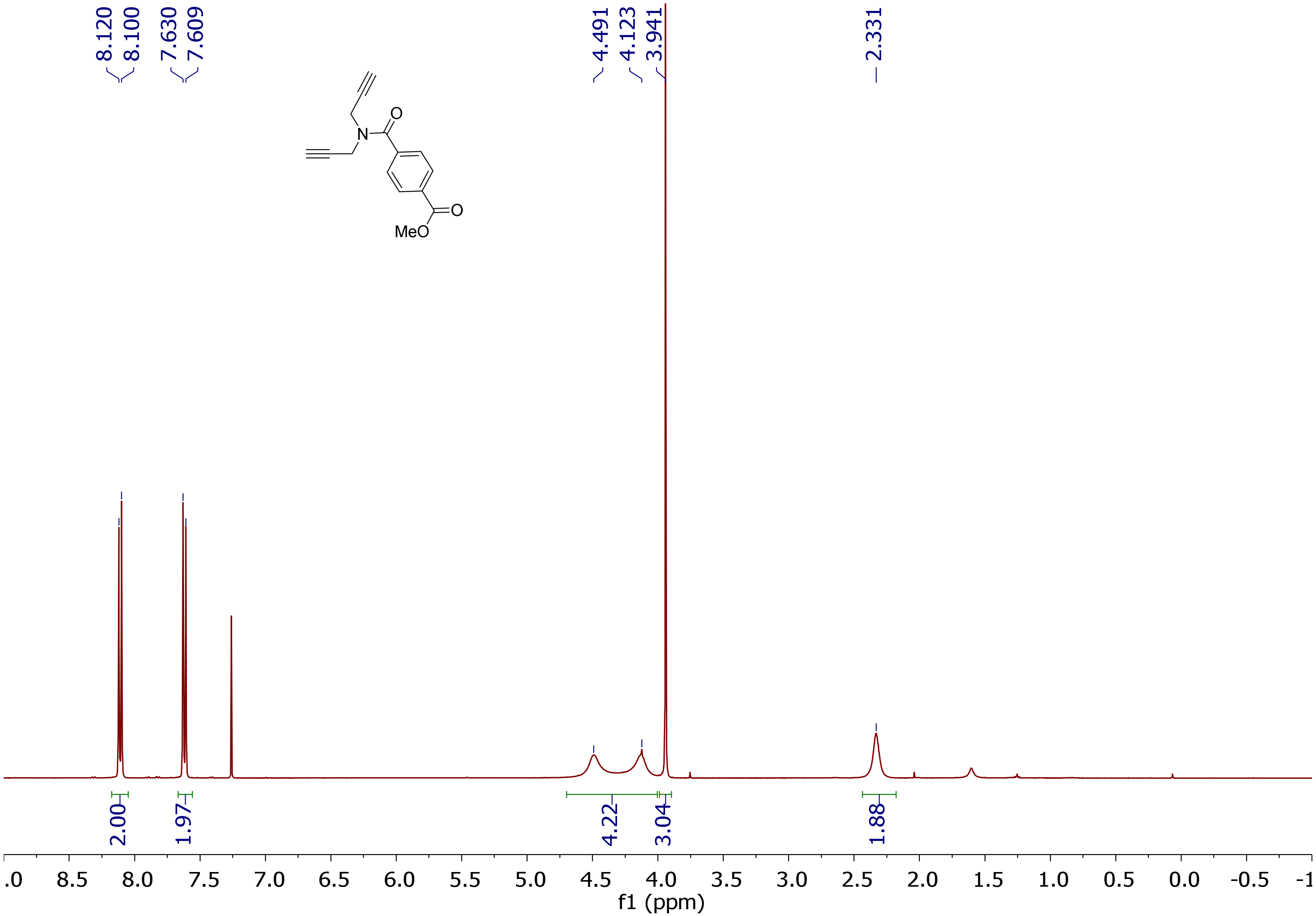

^{13}C-NMR (100.6 MHz, $CDCl_3$) Methyl 4-(di(prop-2-yn-1-yl)carbamoyl)benzoate (S2).

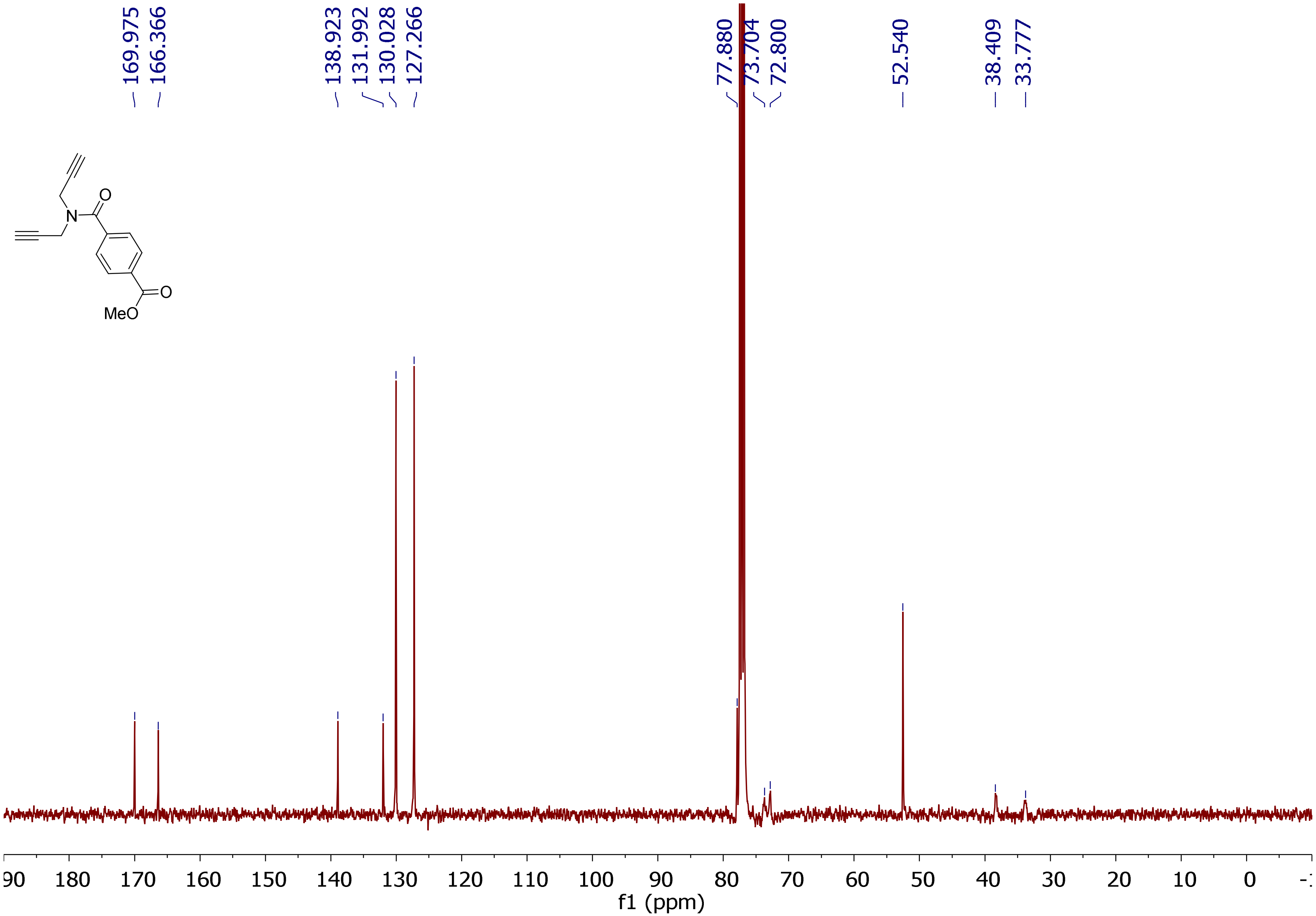

4-(Di(prop-2-yn-1-yl)carbamoyl)benzoic acid (S3).

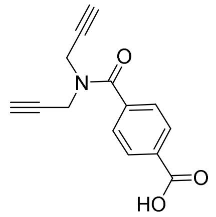

To a solution of **S2** (0.257 g, 1.00 mmol) in MeOH (5 mL) was added NaOH 2N solution (1.51 mL, 3.02 mmol). The reaction was stirred overnight at room temperature and then the solution was carefully quenched with 5% dilute HCl and extracted with EtOAc (3x) followed by washing with H_2O and brine. The organic layer was dried over anhydrous $MgSO_4$ and concentrate under vacuum, affording **S3** (0.224 g, 92 %) as a light yellow solid.

Melting point: 81-83 ^{o}C.

^{1}H NMR (400 MHz, DMSO-*d*$_6$): δ_H = 8.04 (s, 1H), 8.03 (d, 2H, *J* = 8.0 Hz), 7.57 (d, 2H, *J* = 8.0 Hz), 4.31 and 4.10 (bs, rotamers), 3.16 (s, 2H).

^{13}C NMR (100.6 MHz, DMSO-*d*$_6$): δ_C = 169.1, 166.6, 138.7, 132.3, 129.5, 126.9, 78.5, 68.6, 39.2.

HRMS (ES+): calcd for $C_{14}H_{12}NO_3$ 242.0817 [M+H]$^+$, found 242.0823 [M+H]$^+$.

FT-IR (ATR): ν_{max} 3292, 1696, 1639, 1508, 1416, 1280, 1252, 773 and 732 cm^{-1}.

^{1}H-NMR (400 MHz, DMSO-d_6) 4-(Di(prop-2-yn-1-yl)carbamoyl)benzoic acid (S3).

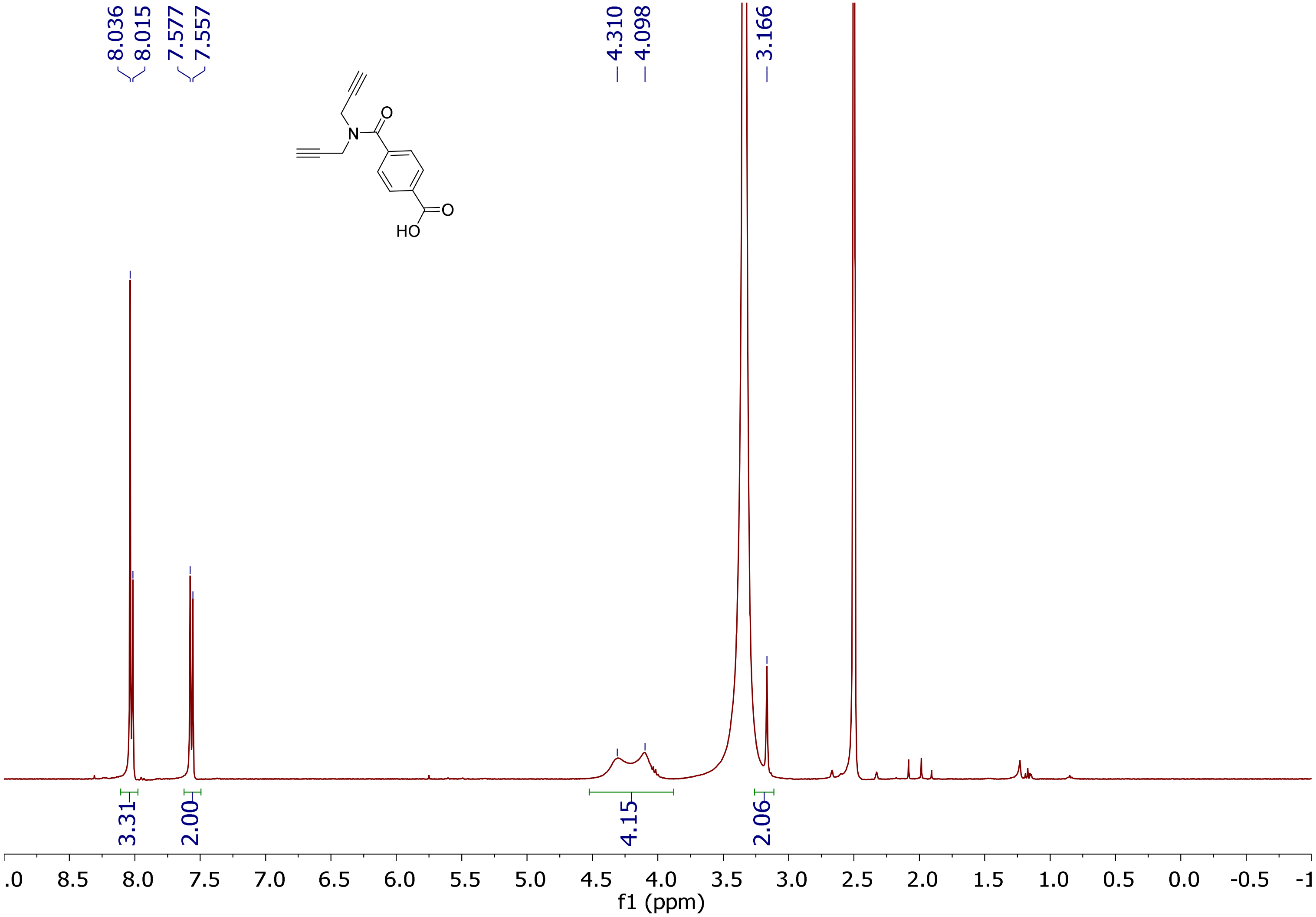

^{13}C-NMR (100.6 MHz, DMSO-d_6) 4-(Di(prop-2-yn-1-yl)carbamoyl)benzoic acid (S3).

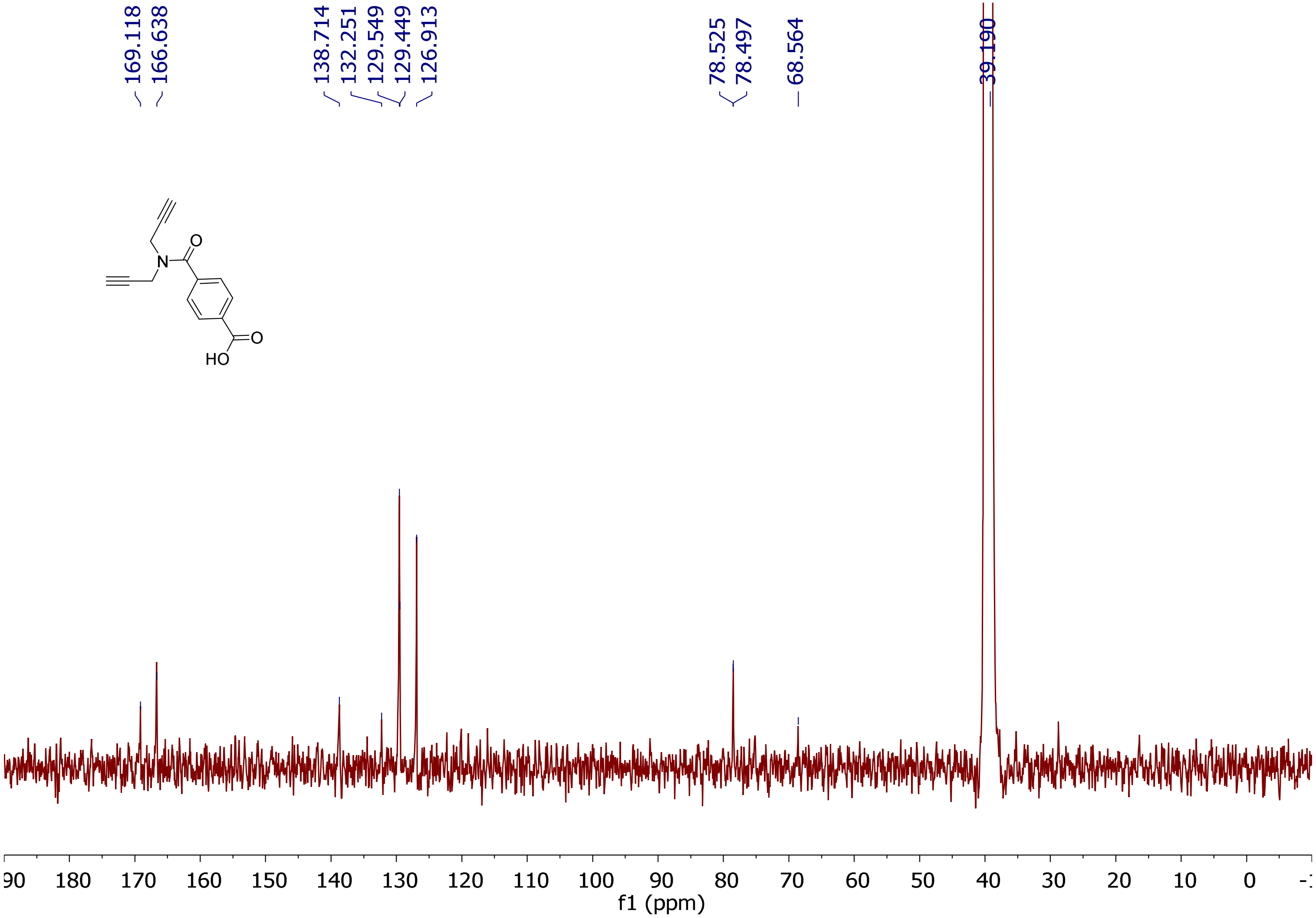

4-((*tert*-Butyldimethylsilyl)oxy)phenol (S4)

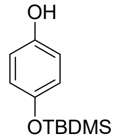

4-(Benzyloxy)phenol (2.5 g, 12.49 mmol) was dissolved in DMF (30 mL) and treated with imidazole (1.70 g, 24.97 mmol) and TBDMS-Cl (2.82 g, 18.73 mmol). The reaction was stirred at room temperature for 4 h. The reaction was diluted with H_2O/EtOAc, washed with 5% aq. soln. LiCl (3x), H_2O (1x) and brine. The organic fraction was dried ($MgSO_4$), and concentrated *in vacuo*. Pd-C (10 wt%, 1.3 g, 1.25 mmol) was added to a solution of the obtained crude in EtOAc (30 mL). The whole system was evacuated and backfilled with H_2 and this protocol was repeated three times. Then the heterogeneous mixture was allowed to stir at 25 °C under a positive pressure of hydrogen. After 1 h, the reaction mixture was filtered directly through Celite. The crude material was purified by flash column chromatography on silica gel (gradient from 0% to 10%of EtOAc in Pet. Ether) to afford **S4** (2.672 g, 95 %) as a white solid.

Melting point: 65-67 ^{o}C.

^{1}H NMR (400 MHz, $CDCl_3$): δ_H = 6.70 (d, 4H, *J* = 2.0 Hz), 4.40 (s, 1H), 0.97 (s, 9H), 0.16 (s, 6H).

^{13}C NMR (100.6 MHz, $CDCl_3$): δ_C = 149.9, 149.6, 120.9, 116.1, 25.9, 18.3, -4.4.

HRMS (ES-): calcd for $C_{12}H_{19}O_2Si$ 223.1154 $[M-H]^-$, found 223.1158 $[M-H]^-$.

FT-IR (ATR): ν_{max} 3333, 2955, 2930, 2859, 1507, 1235, 1213, 911, 829 and 693 cm^{-1}.

^{1}H-NMR (400 MHz, $CDCl_3$) 4-((*tert*-Butyldimethylsilyl)oxy)phenol (S4).

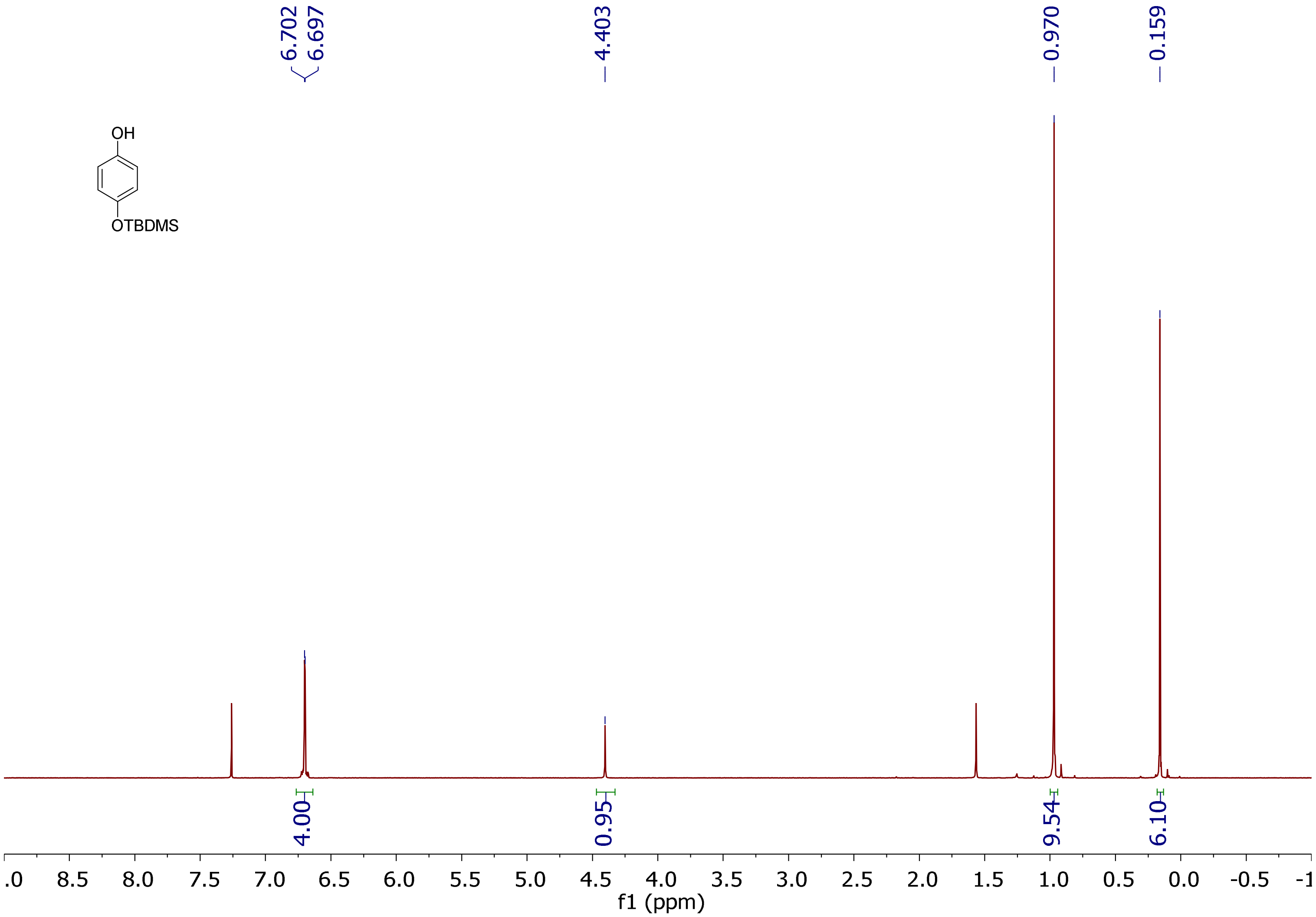

^{13}C-NMR (100.6 MHz, $CDCl_3$) 4-((*tert*-Butyldimethylsilyl)oxy)phenol (S4).

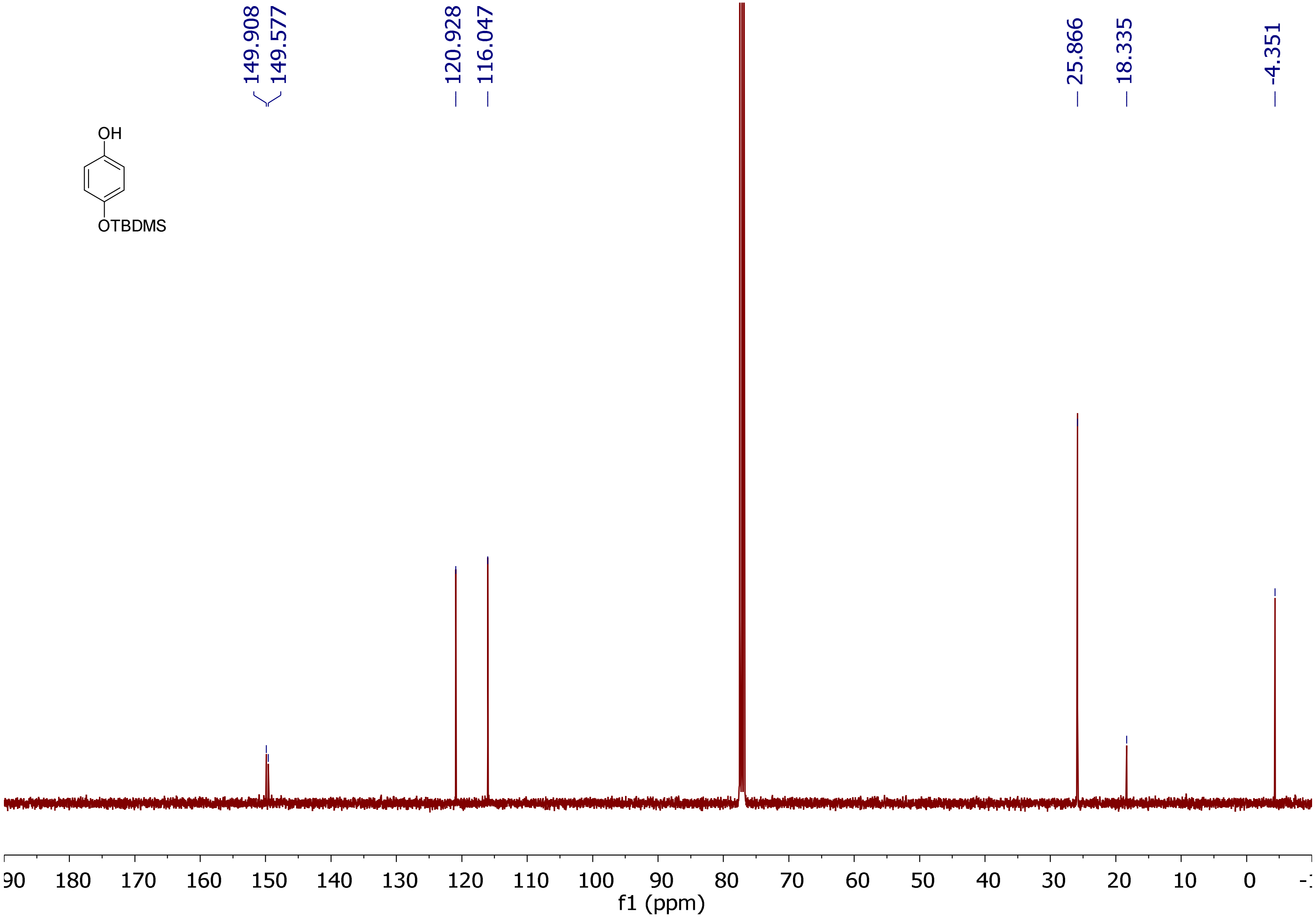

4-((*tert*-Butyldimethylsilyl)oxy)phenyl 4-(di(prop-2-yn-1-yl)carbamoyl)benzoate (S5).

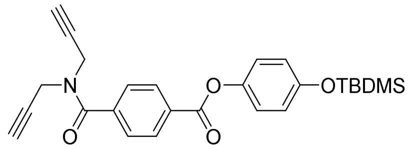

S3 (0.813 g, 3.37 mmol), **S4** (0.756 g, 3.37 mmol), EDC (0.711 g, 3.71 mmol) and DMAP (0.041 g, 0.34 mmol) were dissolved under N_2 atmosphere in dry CH_2Cl_2 (10 ml). The solution was stirred under N_2 atmosphere at room temperature for 1h. The crude was diluted with EtOAc (20 mL) and washed with 5% aq. soln. HCl (3x), H_2O (1x) and brine. The organic phase was dried with $MgSO_4$ and concentrated *in vacuo*. The crude material was purified by flash column chromatography on silica gel (gradient from 0% to 10%of EtOAc in Pet. Ether) to afford **S5** (1.127, 75 %) as a clear oil.

^{1}H NMR (400 MHz, $CDCl_3$): δ_H = 8.26 (d, 2H, *J* = 8.0 Hz), 7.69 (d, 2H, *J* = 8.0 Hz), 7.08 (d, 2H, *J* = 9.0 Hz), 6.87 (d, 2H, *J* = 9.0 Hz), 4.51 and 4.16 (bs, 4H, rotamers), 2.35 (s, 2H), 1.00 (s, 9H), 0.22 (s, 6H).

^{13}C NMR (100.6 MHz, $CDCl_3$): δ_C = 169.9, 164.7, 153.7, 144.8, 139.5, 131.6, 130.6, 127.4, 122.4, 120.8, 77.9, 73.8, 38.6, 25.8, 18.4, -4.3.

HRMS (ES+): calcd for $C_{26}H_{30}NO_4Si$ 448.1944 $[M+H]^+$, found 448.1938 $[M+H]^+$.

FT-IR (ATR): ν_{max} 3295, 2955, 2931, 2858, 1737, 1650, 1501, 1258, 1191, 1075, 913, 840, 831 and 779 cm^{-1}.

^{1}H-NMR (400 MHz, $CDCl_3$) 4-((*tert*-Butyldimethylsilyl)oxy)phenyl 4-(di(prop-2-yn-1-yl)carbamoyl)benzoate (S5).

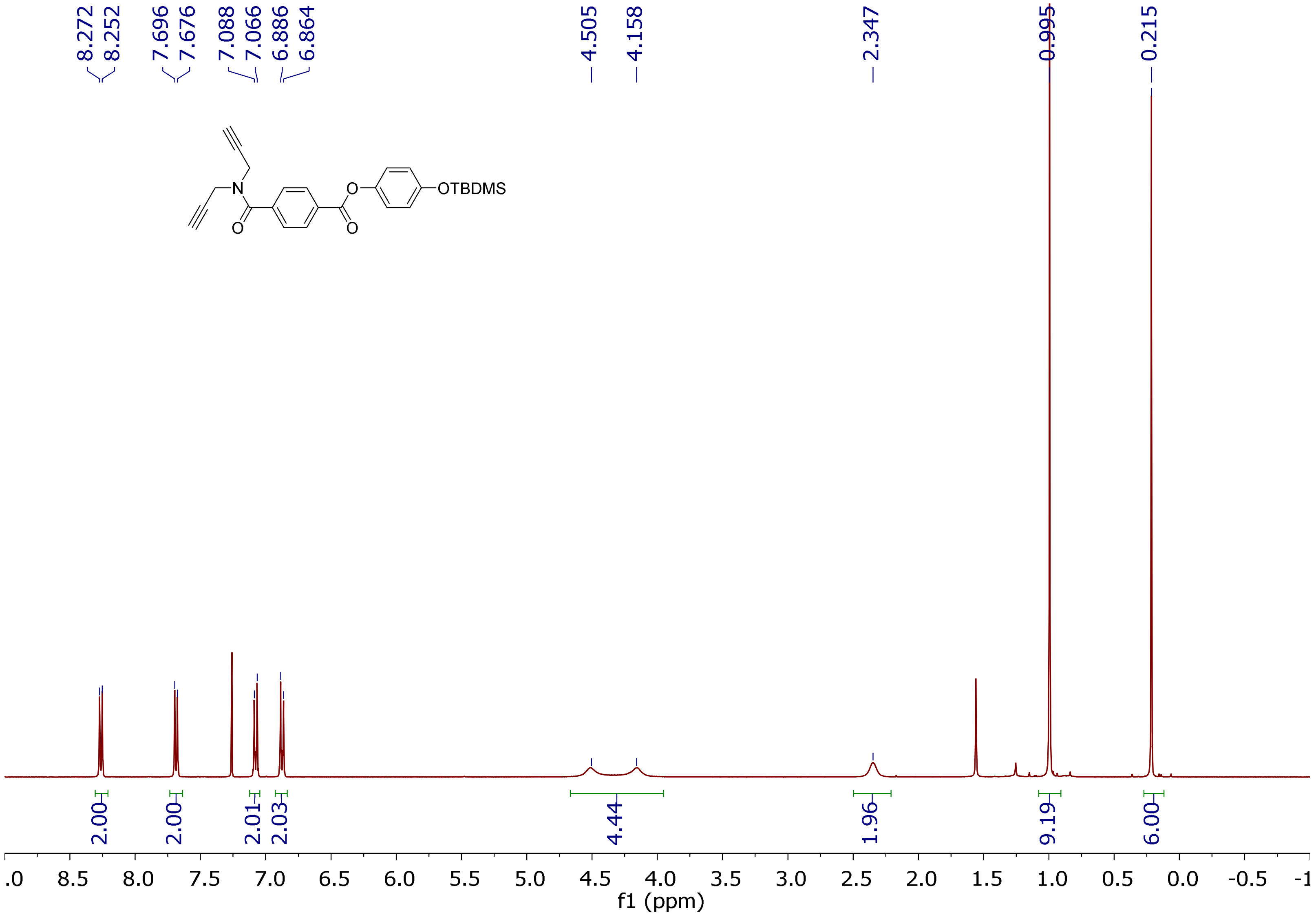

^{13}C-NMR (100.6 MHz, $CDCl_3$) 4-((*tert*-Butyldimethylsilyl)oxy)phenyl 4-(di(prop-2-yn-1-yl)carbamoyl)benzoate (S5).

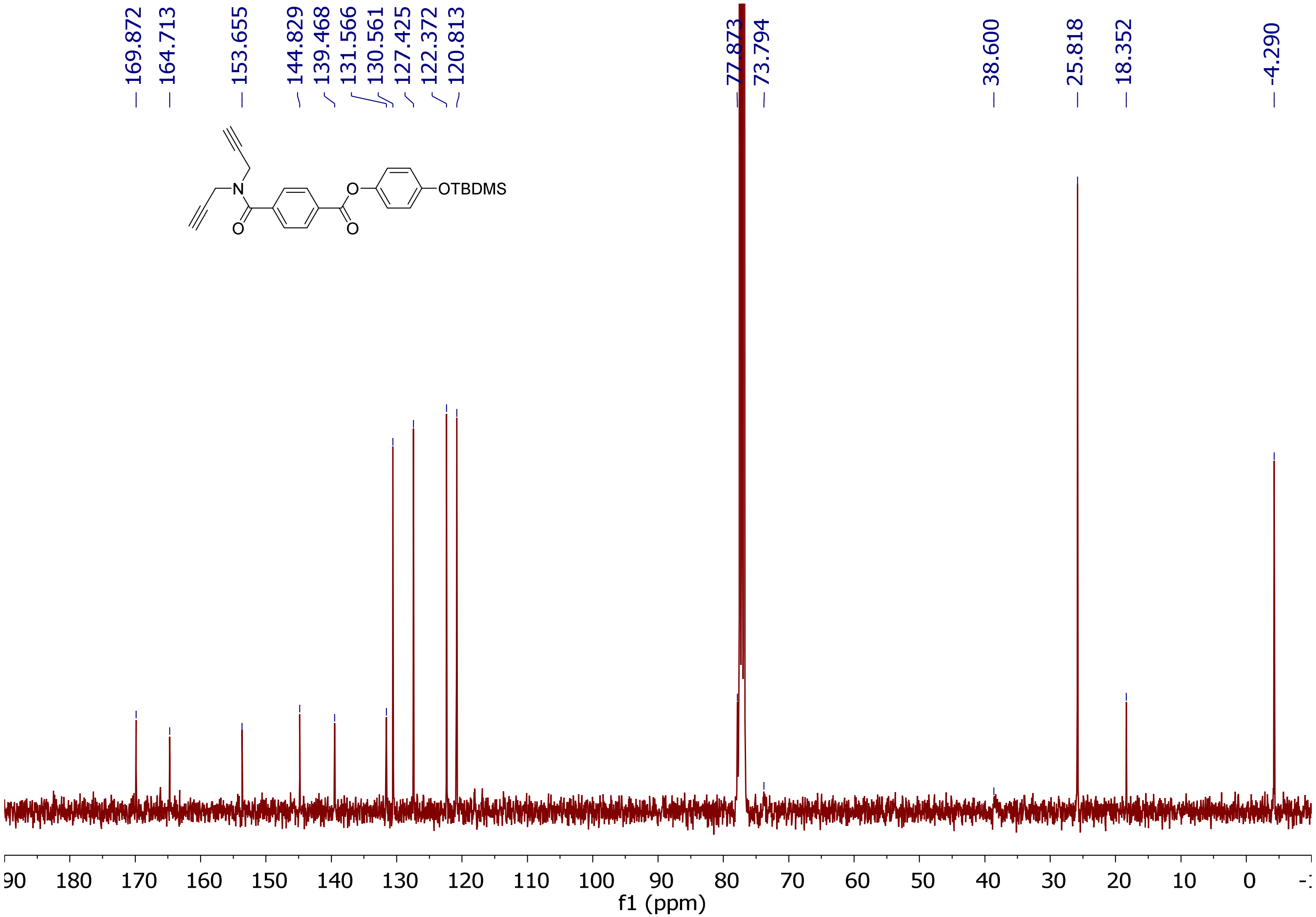

Methyl 4-(((1-(3,5-di-*tert*-butylbenzyl)-1H-1,2,3-triazol-4-yl)methyl)(prop-2-yn-1-yl)carbamoyl)benzoate (S7).

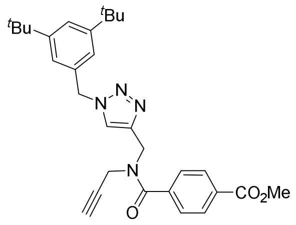

Compound **S2** (0.431 g, 1.69 mmol), $Cu(CH_3CN)_4PF_6$ (0.011 g, 0.03 mmol) and TBTA (0.016 g, 0.03 mmol) were mixed in a round-bottom flask and, under N_2, THF (35 mL) was added. A solution of azide **S6** (0.083 g, 0.34 mmol) in THF (2 mL) was added and the reaction was stirred overnight at room temperature. The solvent was evaporated to dryness and the crude was purified by flash column chromatography on silica gel (gradient from 10% to 100%of EtOAc in Pet. Ether) to afford **S7** (0.100 g, 62%) as a clear syrup and the corresponding disubstituted compound (**S8**, 0.055 g, 23%) as a foam.

^{1}H NMR (400 MHz, CDCl$_3$): δ_H = 8.07 (d, 2H, *J* = 8.0 Hz), 7.64 (s partially overlapped, 1H), 7.57 (d, 2H, *J* = 8.0 Hz), 7.42 (s, 1H), 7.10 (s, 2H), 5.50 (s, 2H), 4.85, 4.63, 4.35 and 4.06 (bs, 4H, rotamers), 3.93 (s, 3H), 2.32 and 2.22 (bs, 1H, rotamers), 1.30 (s, 18H).

^{13}C NMR (125.7 MHz, CDCl$_3$): δ_C = 170.3, 166.4, 152.0, 143.7, 139.3, 133.8, 131.9, 130.0, 127.5, 127.3, 123.6, 123.0, 122.5, 78.5, 73.5, 55.0, 52.5, 40.1 and 39.3 (rotamers), 35.1, 31.5.

HRMS (ES+): calcd for $C_{30}H_{37}N_4O_3$ 501.2866 [M+H]$^+$, found 501.2856 [M+H]$^+$.

FT-IR (ATR): ν_{max} 2955, 1725, 1639, 1456, 1277, 1249, 1110 and 754 cm^{-1}.

^{1}H-NMR (400 MHz, $CDCl_3$) Methyl 4-(((1-(3,5-di-*tert*-butylbenzyl)-1H-1,2,3-triazol-4-yl)methyl)(prop-2-yn-1-yl)carbamoyl)benzoate (S7).

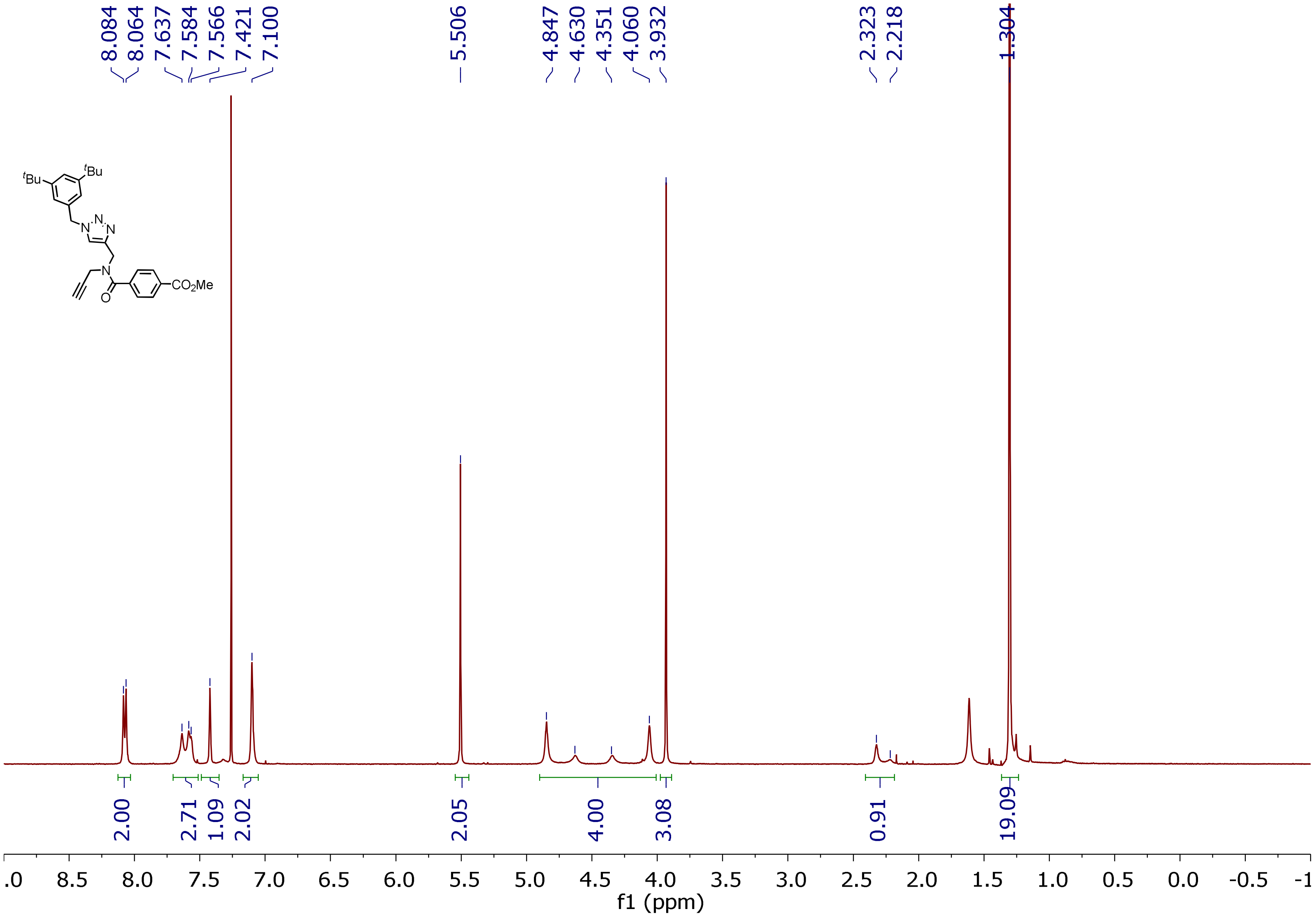

^{13}C-NMR (125.7 MHz, $CDCl_3$) Methyl 4-(((1-(3,5-di-*tert*-butylbenzyl)-1H-1,2,3-triazol-4-yl)methyl)(prop-2-yn-1-yl)carbamoyl)benzoate (S7).

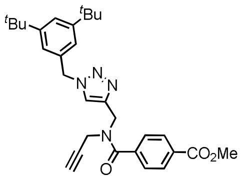

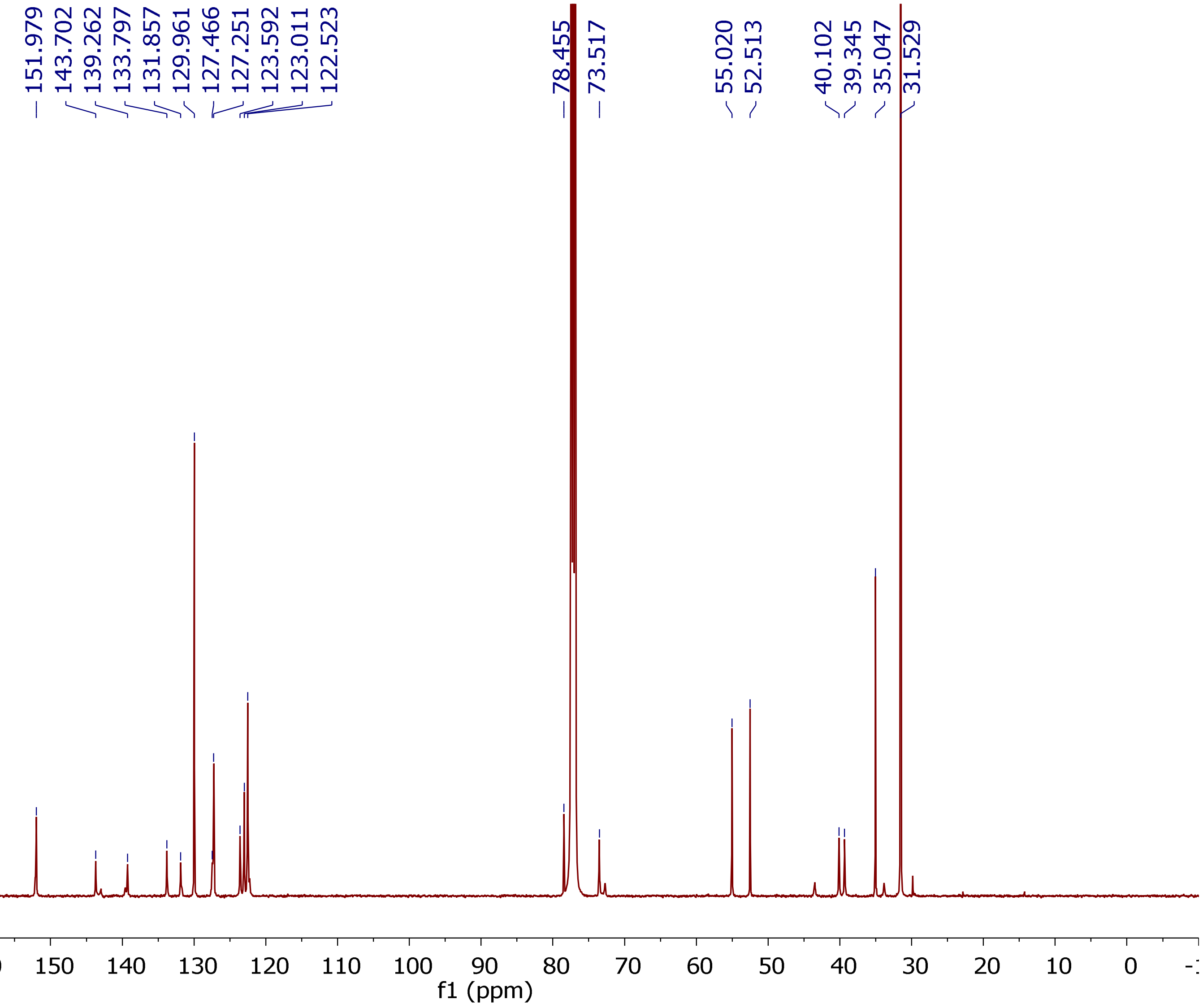

Methyl 4-(bis((1-(3,5-di-*tert*-butylbenzyl)-1H-1,2,3-triazol-4-yl)methyl)carbamoyl)benzoate (S8).

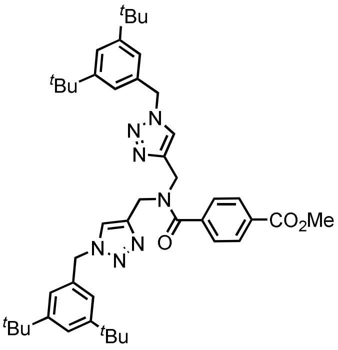

Melting point: 193-195 °C.

^{1}H NMR (400 MHz, $CDCl_3$): δ_H = 8.04 (d, 2H, J = 8.0 Hz), 7.70 (d, 2H, J = 8.0 Hz), 7.68 and 7.57 (s, 2H, rotamers), 7.42 (s, 2H), 7.10 (s, 4H), 5.51 and 5.49 (bs, 4H, rotamers), 4.66 and 4.52 (bs, 4H, rotamers), 3.90 (s, 3H,), 1.30 (s, 38H).

^{13}C NMR (100.6 MHz, $CDCl_3$): δ_C = 170.8, 166.4, 151.9 and 151.8 (rotamers), 143.8 and 143.0 (rotamers), 133.7, 131.4, 130.0, 127.7, 123.7, 123.0, 122.9, 122.4, 54.9, 52.3, 43.8 and 39.3 (rotamers), 35.0, 31.5.

HRMS (ES+): calcd for $C_{45}H_{60}N_7O_3$ 746.4758 $[M+H]^+$, found 746.4754 $[M+H]^+$.

FT-IR (ATR): ν_{max} 2961, 2867, 1725, 1634, 1601, 1277, 1243, 1110, 1049, 752 cm^{-1}.

^{1}H-NMR (400 MHz, $CDCl_3$) Methyl 4-(bis((1-(3,5-di-tert-butylbenzyl)-1H-1,2,3-triazol-4-yl)methyl)carbamoyl)benzoate (S8).

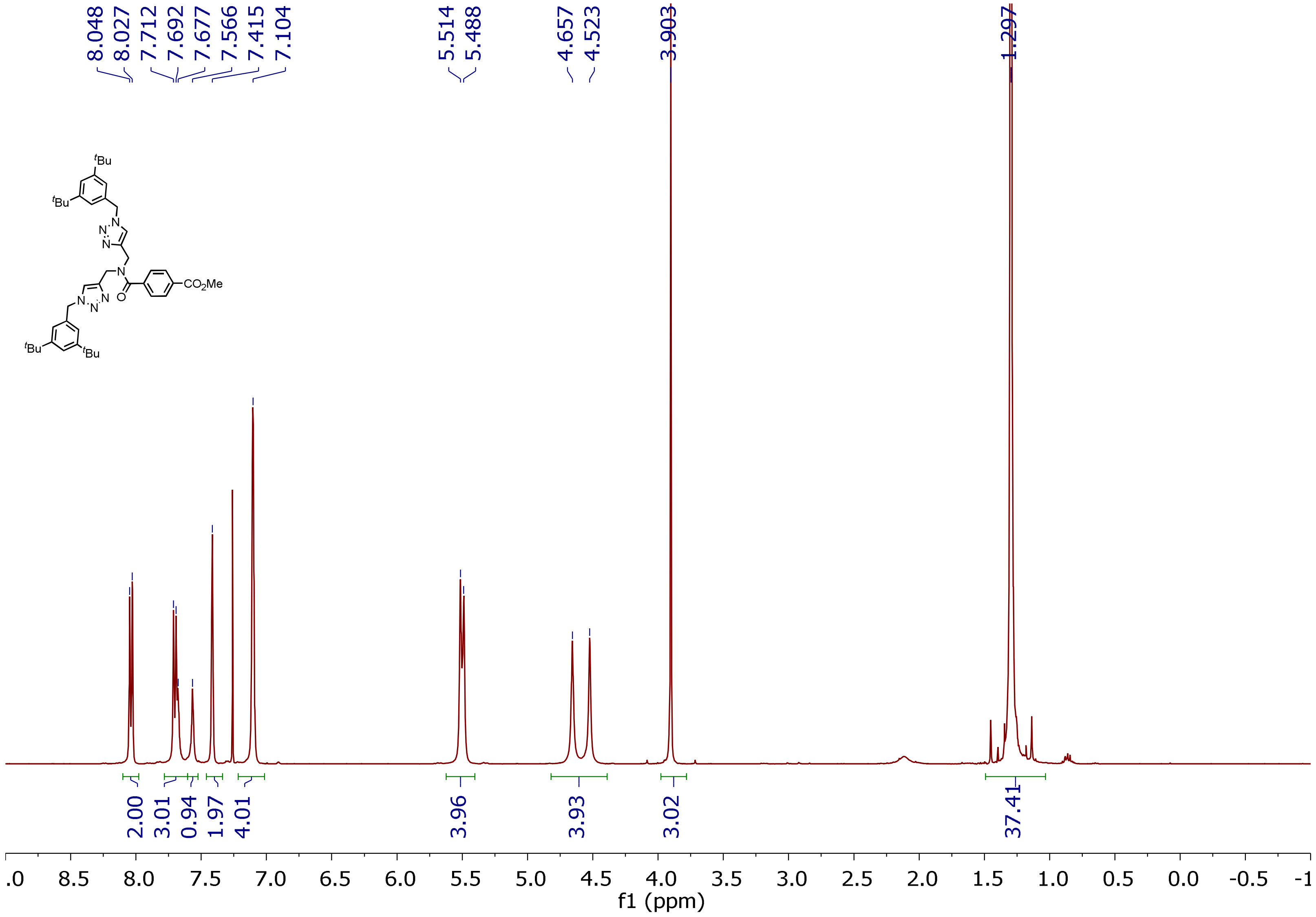

^{13}C-NMR (100.6 MHz, $CDCl_3$) Methyl 4-(bis((1-(3,5-di-tert-butylbenzyl)-1H-1,2,3-triazol-4-yl)methyl)carbamoyl)benzoate (S8).

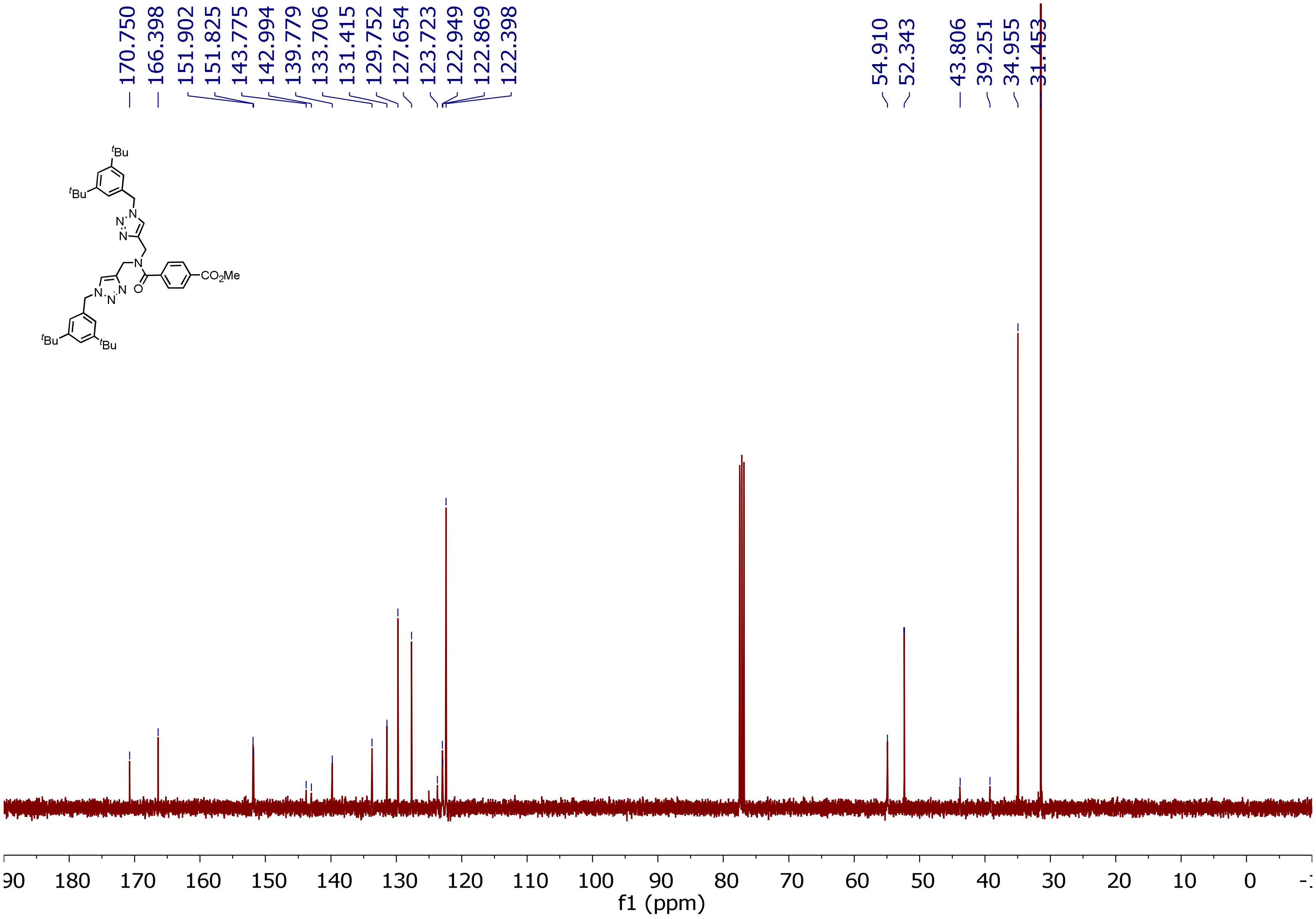

Compound S10-ester.

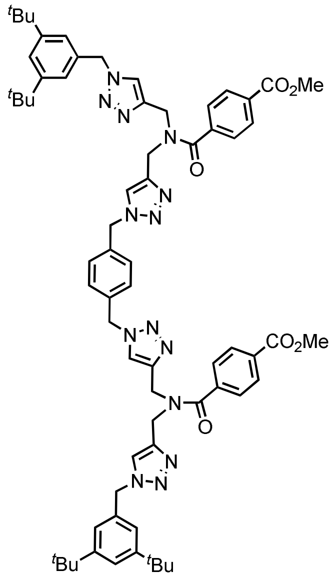

Compound **S7** (0.107 g, 0.21 mmol), $Cu(CH_3CN)_4PF_6$ (0.004 g, 0.01 mmol) and TBTA (0.005 g, 0.01 mmol) were mixed in a round-bottom flask and, under N_2, THF (2 mL) was added. A solution of azide **S9** (0.019 g, 0.10 mmol) in THF (0.5 mL) was added and the reaction was stirred overnight at room temperature. The solvent was evaporated to dryness and the crude was purified by flash column chromatography on silica gel (gradient from 0% to 6% of MeOH in CH_2Cl_2) to afford **S10-ester** (0.121 g, quantitative) as a foam.

^{1}H NMR (400 MHz, CDCl$_3$): δ_H = 8.04 (d, 4H, *J* = 8.0 Hz), 7.70 (d, 4H, *J* = 8.0 Hz), 7.67 (s partially overlapped, 2H), 7.58 and 7.51 (s, 2H), 7.42 (s, 2H), 7.29 (s, 4H), 7.10 (s, 4H), 5.50 (m, 8H), 4.65 and 4.53 (bs, 8H, rotamers), 3.91 (s, 6H), 1.30 (s, 36H).

^{13}C NMR (125.7 MHz, CDCl$_3$): δ_C = 170.9, 166.5, 152.0 and 151.9 (rotamers), 143.4 and 143.1 (rotamers), 139.8 (4-C), 135.4, 133.7, 131.5, 129.9, 129.0, 128.9, 127.7, 123.9, 123.3, 123.0, 122.5, 55.0 and 53.8 (rotamers), 52.5, 44.2, 43.8 and 39.6 (rotamers), 35.0, 31.5.

HRMS (ES+): calcd for $C_{68}H_{81}N_{14}O_6$ 1189.6464 $[M+H]^+$, found 1189.6434 $[M+H]^+$.

FT-IR (ATR): ν_{max} 2955, 2867, 1724, 1634, 1460, 1435, 1278, 1247 and 754 cm^{-1}.

^{1}H-NMR (400 MHz, $CDCl_3$) Compound S10-ester.

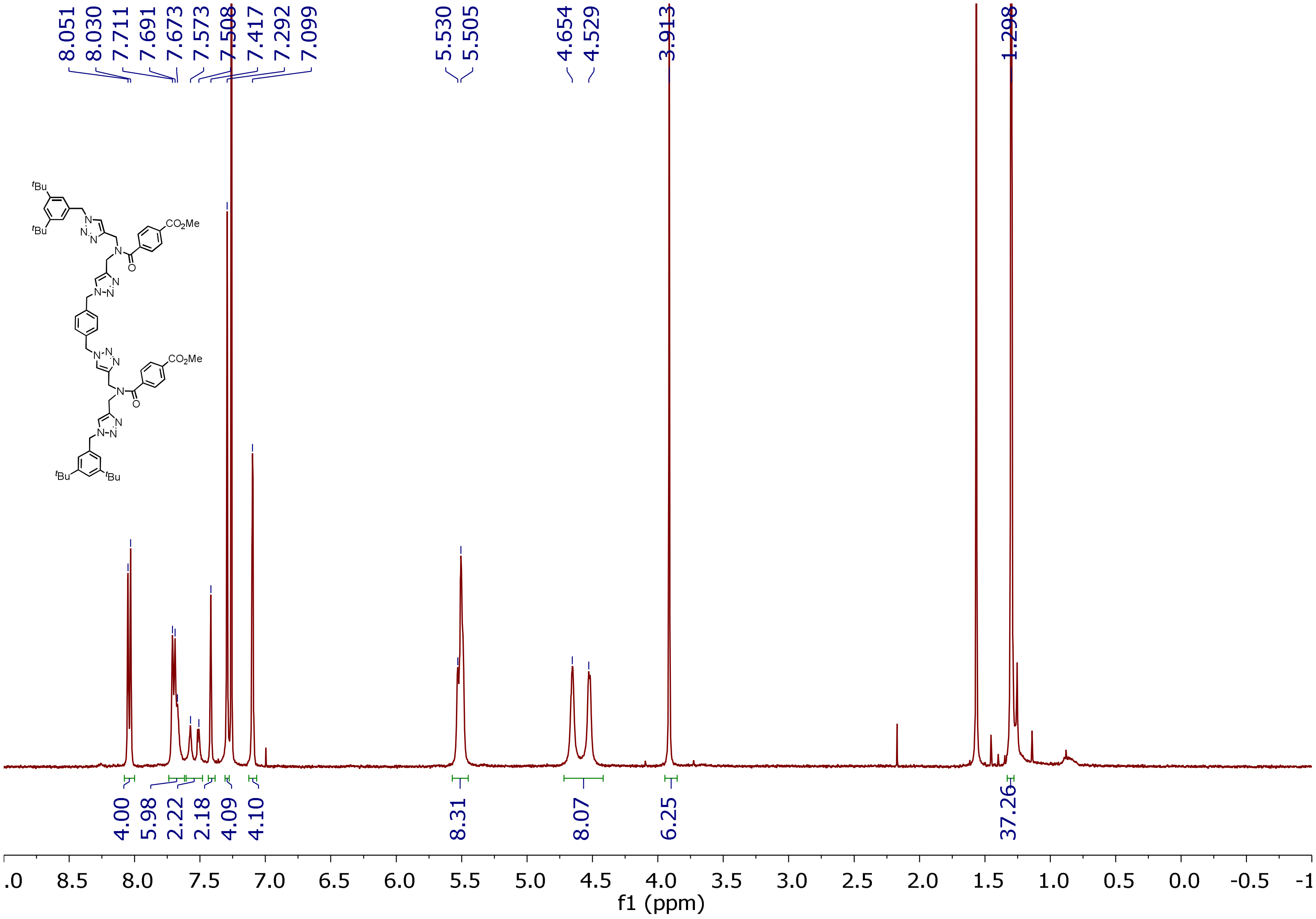

^{13}C-NMR (125.7 MHz, $CDCl_3$) Compound S10-ester.

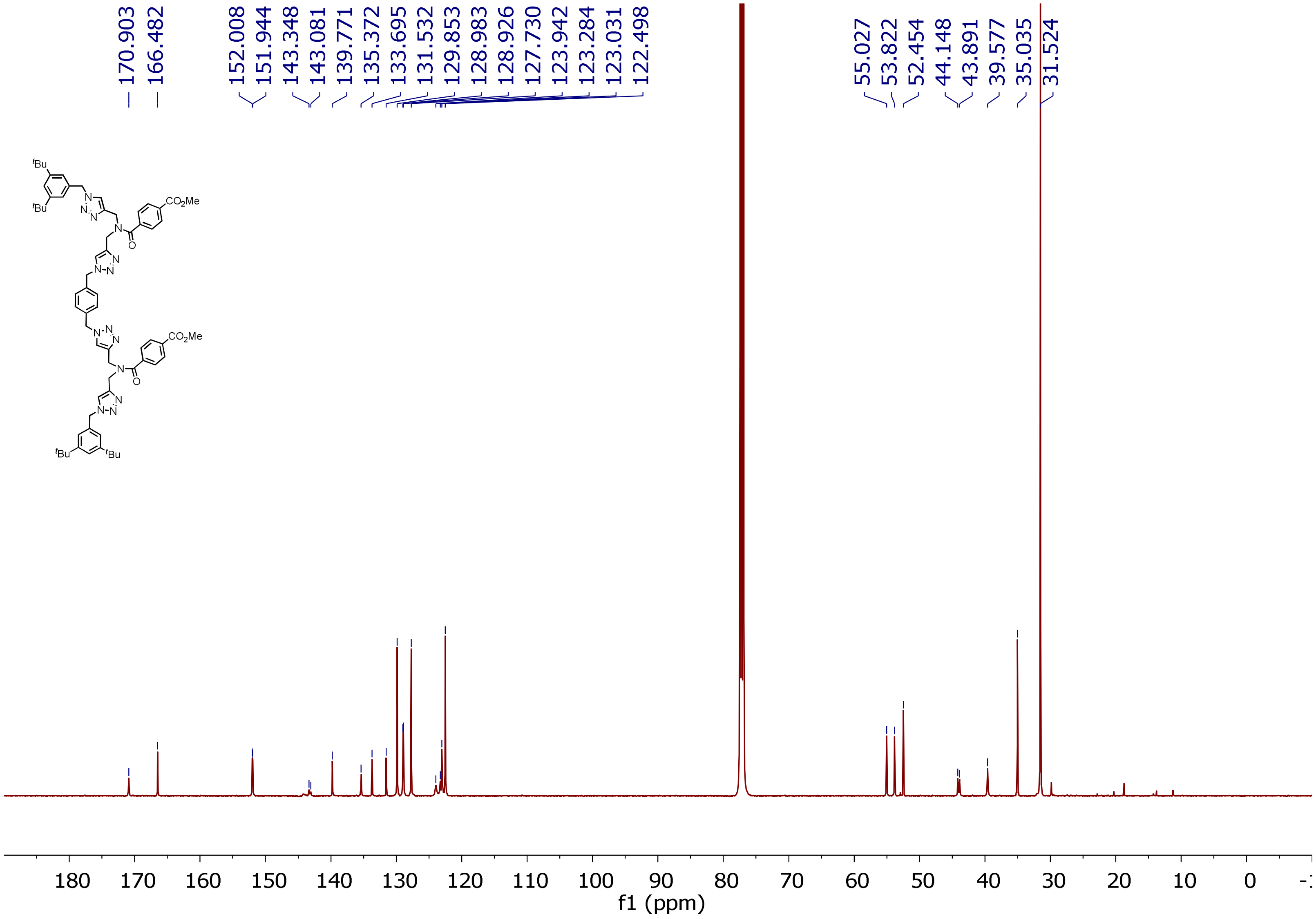

Compound S10.

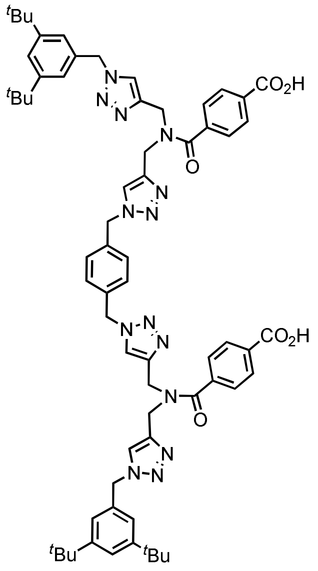

To a solution of **S10-ester** (0.790 g, 0.66 mmol) in MeOH (15 mL) was added NaOH 2N solution (1.99 mL, 3.99 mmol). The reaction was stirred overnight at room temperature and then the solution was carefully quenched with 5% dilute HCl and extracted with EtOAc (3x) followed by washing with H_2O and brine. The organic layer was dried over anhydrous $MgSO_4$ and concentrate under vacuum, affording compound **S10** (0.731 g, 95 %) as a white solid.

Melting point: 142-144°C.

^{1}H NMR (400 MHz, CDCl$_3$): δ_H = 7.94 (m, 4H), 7.79-7-51 (m, 5H), 7.44 (bs, partially overlapped, 3H), 7.42 (s, 2H), 7.26 (s, overlapped, 4H), 7.12 (s, 4H), 5.53 (m, 8H), 4.92 and 4.58 (bs, 8H, rotamers), 1.30 (s, 36H).

^{13}C NMR (100.6 MHz, CDCl$_3$): δ_C = 171.3, 168.5, 151.9, 143.7, 139.7, 135.4, 133.5, 131.6, 130.2, 128.8, 127.3, 124.3, 123.3, 123.0, 122.5, 55.1, 53.8, 44.1 and 40.2 (rotamers), 35.0, 31.5.

HRMS (ES+): calcd for $C_{66}H_{77}N_{14}O_6$ 1161.6151 $[M+H]^+$, found 1161.6135 $[M+H]^+$.

FT-IR (ATR): ν_{max} 2964, 2867, 1709, 1629, 1246, 1218, 1051 and 750 cm^{-1}.

^{1}H-NMR (400 MHz, $CDCl_3$) Compound S10.

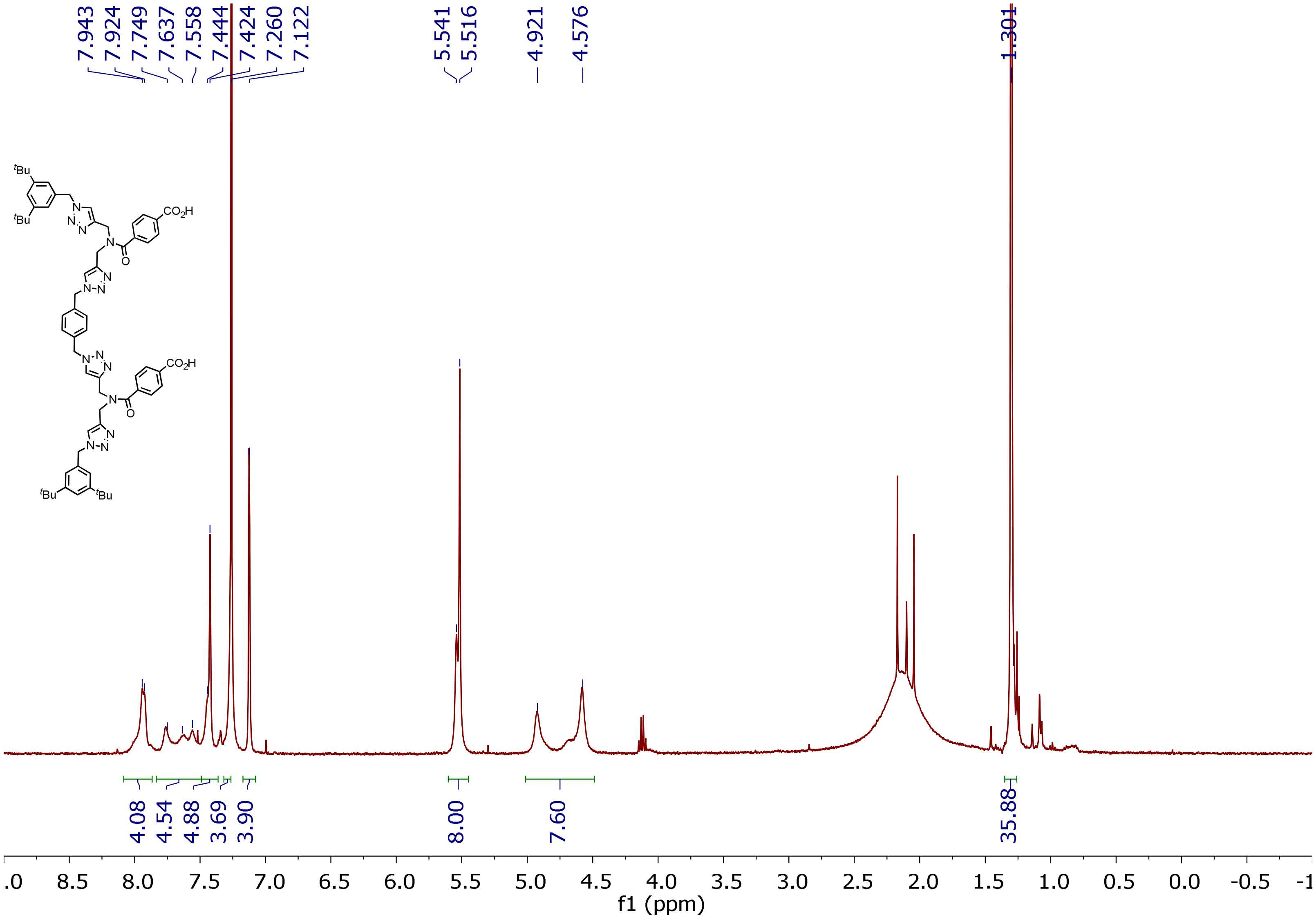

^{13}C-NMR (100.6 MHz, $CDCl_3$) Compound S10.

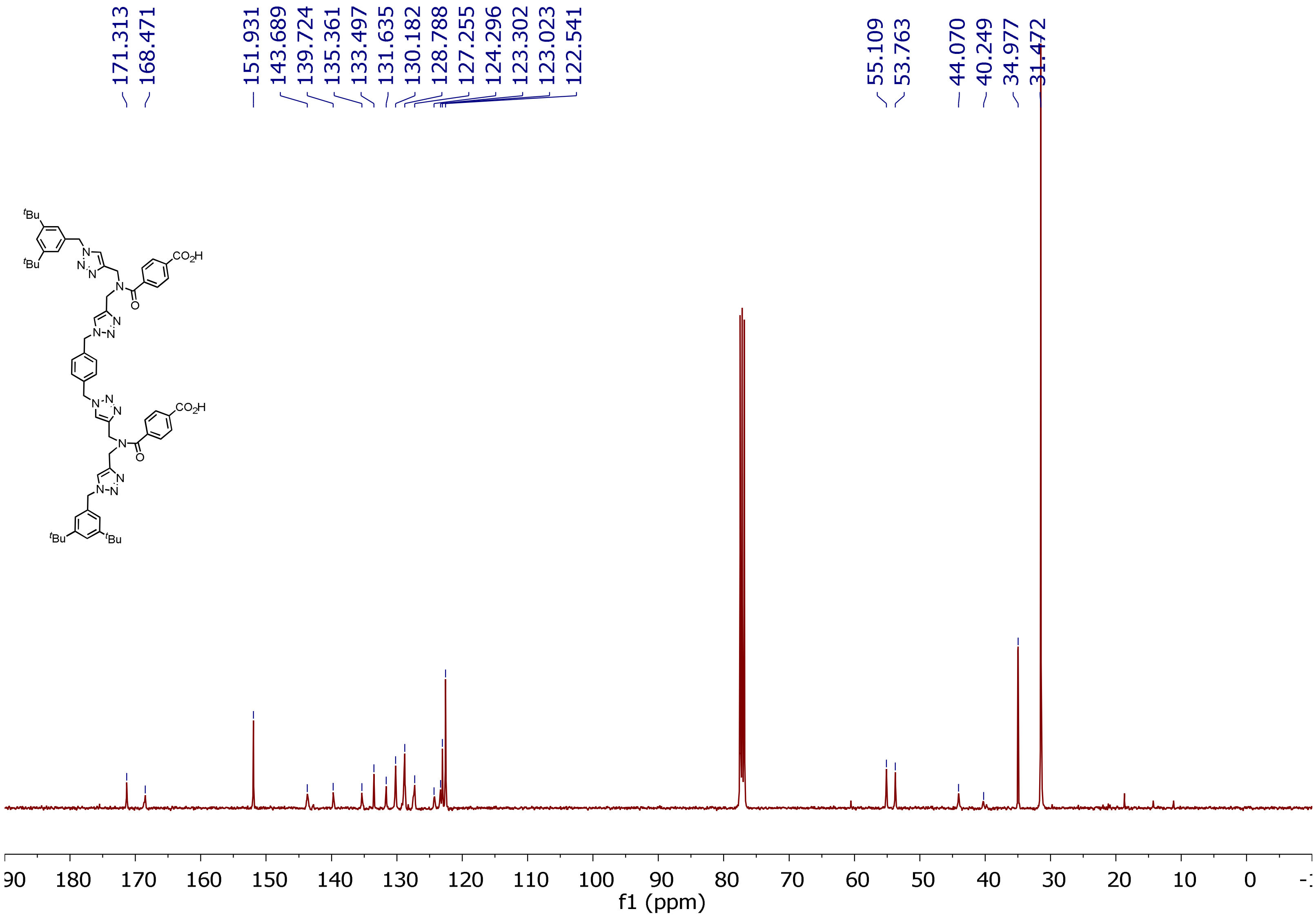

Compound S11.

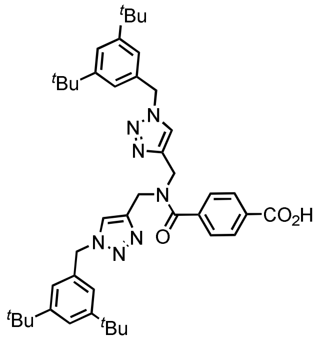

To a solution of **S8** (0.133 g, 0.178 mmol) in a mixture of MeOH (5 mL) and $CHCl_3$ (1 mL) was added NaOH 2N solution (0.267 mL, 0.534 mmol). The reaction was stirred overnight at 60 ^oC and then the solution was carefully quenched with 5% dilute HCl and extracted with EtOAc (3x) followed by washing with H_2O and brine. The organic layer was dried over anhydrous $MgSO_4$ and concentrate under vacuum, affording **S11** (0.110 g, 85 %) as a light yellow solid.

Melting point: 85-87 ^oC.

^{1}H NMR (400 MHz, $CDCl_3$): δ_H = 8.06 (d, 2H, *J* = 8.0 Hz), 7.67 (s partially overlapped, 2H), 7.66 (d, 2H, *J* = 8.0 Hz), 7.42 (s, 2H), 7.11 (s, 4H), 5.52 and 5.50 (bs, 4H, rotamers), 4.69 and 4.60 (bs, 4H, rotamers), 1.30 (s, 38H).

^{13}C NMR (100.6 MHz, $CDCl_3$): δ_C = 171.0, 169.3, 152.0 , 140.2, 133.7, 133.6, 131.1, 130.4, 127.7, 123.9, 123.4, 123.1, 123.0, 122.5, 55.2 and 55.1 (rotamers), 43.8 and 39.5 (rotamers), 35.1, 31.5.

HRMS (ES+): calcd for $C_{44}H_{58}N_7O_3$ 732.4601 $[M+H]^+$, found 732.4604 $[M+H]^+$.

FT-IR (ATR): ν_{max} 2963, 2906, 2867, 1716, 1636, 1248, 1221 and 772 cm^{-1}.

^{1}H-NMR (400 MHz, $CDCl_3$) Compound S11.

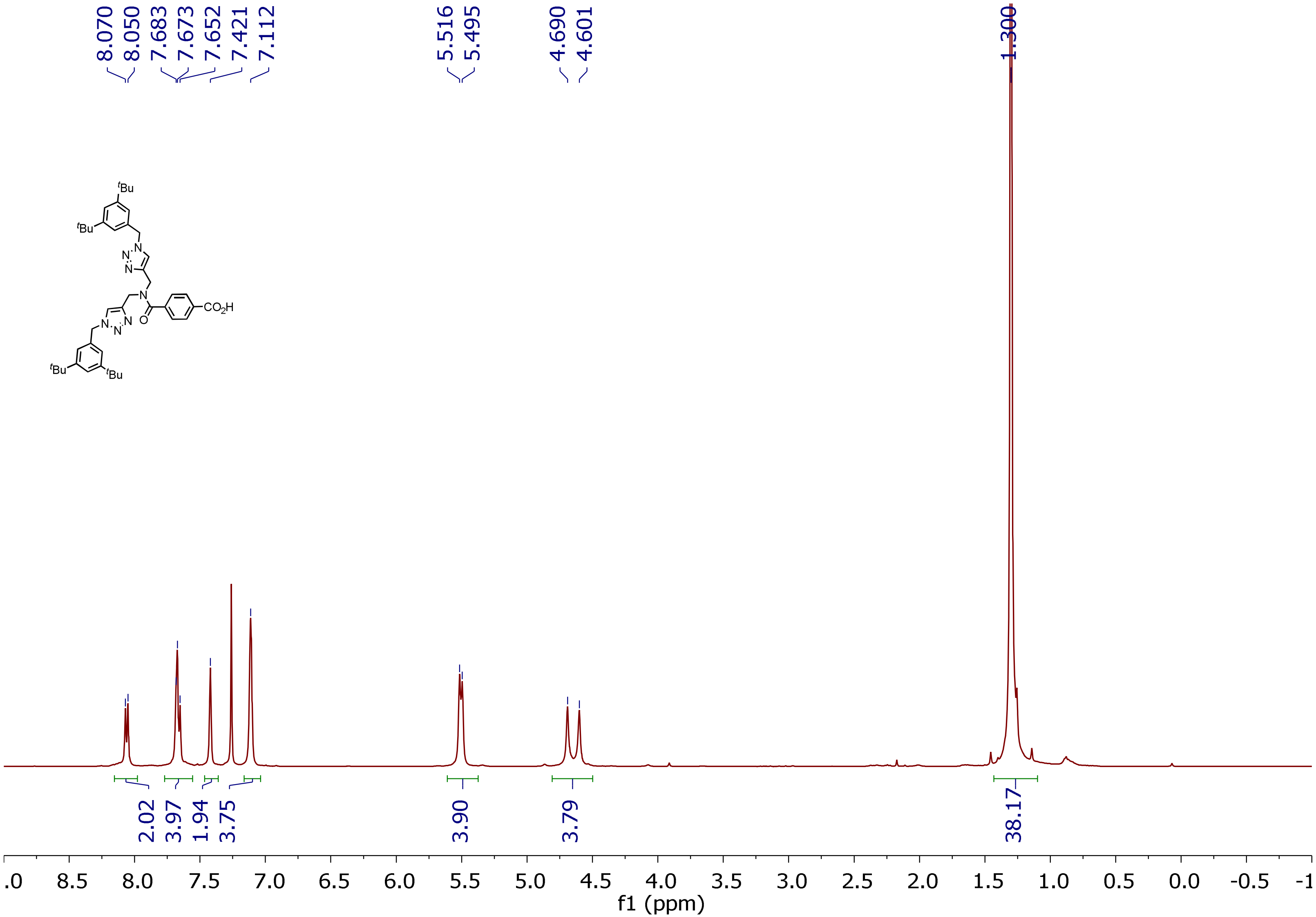

^{13}C-NMR (100.6 MHz, $CDCl_3$) Compound S11.

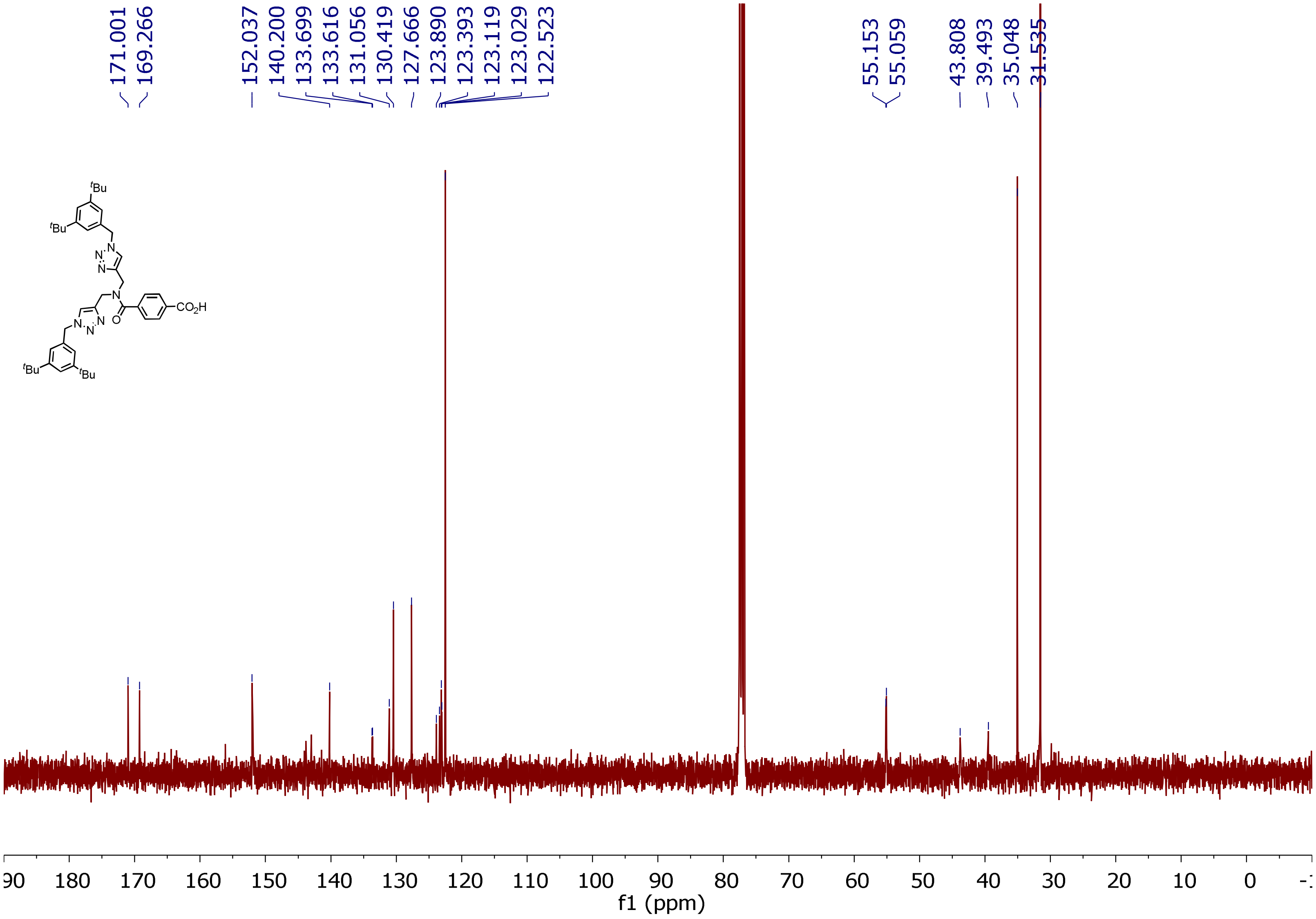

Compound S12.

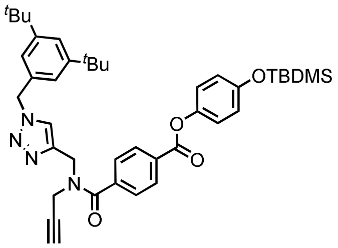

Compound **S5** (1.126 g, 2.23 mmol), $Cu(CH_3CN)_4PF_6$ (0.011 g, 0.03 mmol) and TBTA (0.016 g, 0.03 mmol) were mixed in a round-bottom flask and, under N_2, THF (32 mL) was added. A solution of 1-(azidomethyl)-3,5-di-*tert*-butylbenzene **S6** (0.077 g, 0.31 mmol) in THF (2 mL) was added and the reaction was stirred overnight at room temperature. The solvent was evaporated to dryness and the crude was purified by flash column chromatography on silica gel (gradient from 10% to 100%of EtOAc in Pet. Ether) to afford **S12** (0.147 g, 66%) as a foam and the corresponding disubstituted compound **S13** (0.065 g, 22%) as a foam.

^{1}H NMR (400 MHz, $CDCl_3$): δ_H = 8.22 (d, 2H, *J* = 8.0 Hz), 7.71 (s partially overlapped, 1H), 7.64 (d, 2H, *J* = 8.0 Hz), 7.42 (t, 1H, *J* = 2.0 Hz), 7.11 (s, 2H), 7.07 (d, 2H, *J* = 9.0 Hz), 6.87 (d, 2H, *J* = 9.0 Hz), 5.51 (s, 2H), 4.86, 4.65, 4.37 and 4.09 (bs, 4H, rotamers), 2.34 and 2.24 (bs, 1H, rotamers), 1.31 (s, 18H), 0.99 (s, 9H), 0.21 (s, 6H).

^{13}C NMR (125.7 MHz, $CDCl_3$): δ_C = 170.2, 164.8, 153.6, 152.0, 144.8, 143.7, 139.8, 131.4, 130.5, 127.6, 127.4, 123.6, 123.0, 122.5, 122.4, 120.8, 78.4, 73.6, 55.0, 40.1 and 39.4 (rotamers), 35.1, 31.5, 29.9, 25.8, 18.3, -4.3.

HRMS (ES+): calcd for $C_{41}H_{53}N_4O_4Si$ 693.3836 $[M+H]^+$, found 693.3834 $[M+H]^+$.

FT-IR (ATR): ν_{max} 2960, 2931, 2860, 1739, 1645, 1502, 1259, 1192, 1075 and 775 cm^{-1}.

^{1}H-NMR (400 MHz, $CDCl_3$) Compound S12.

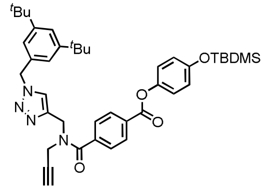

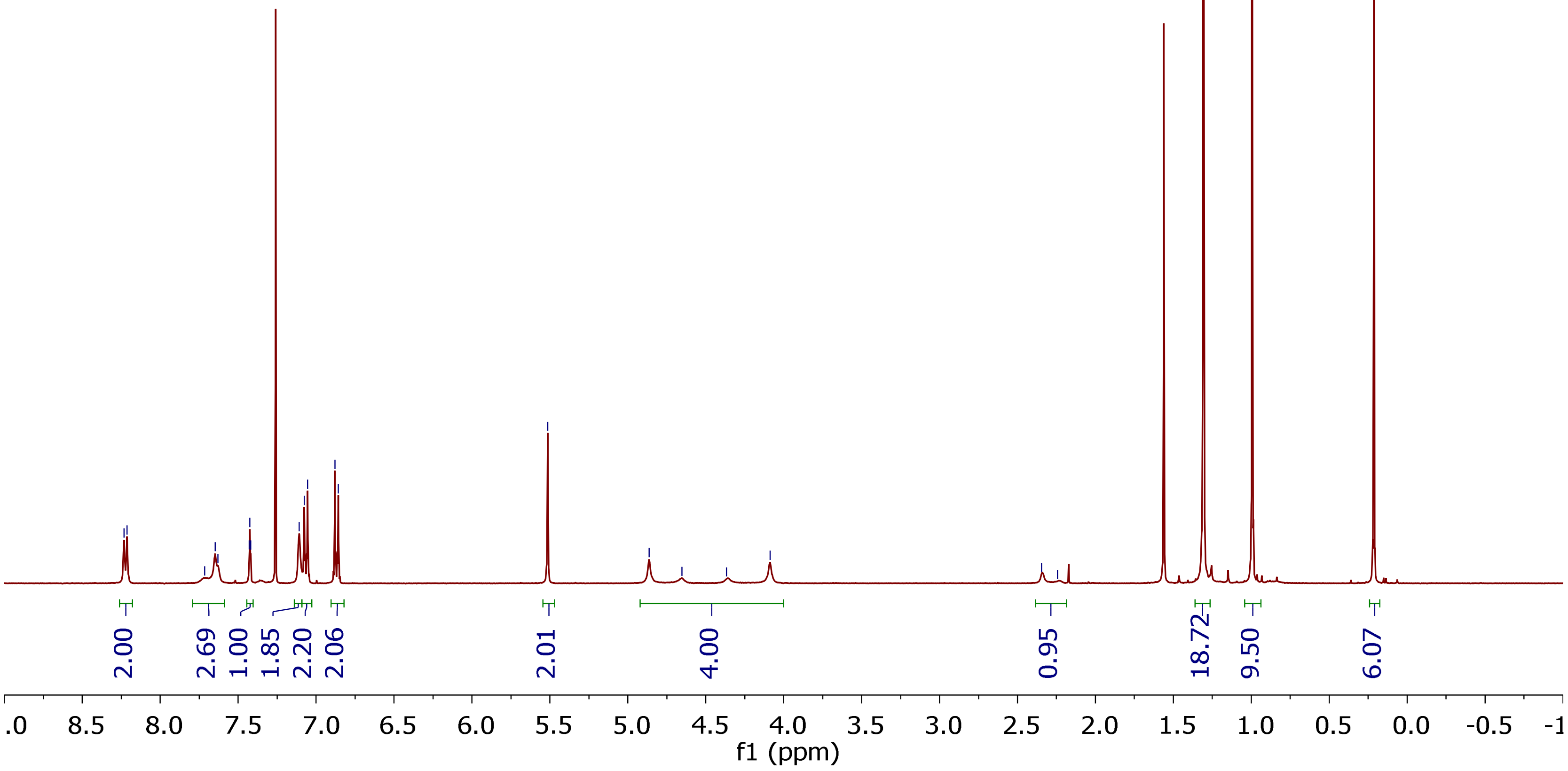

^{13}C-NMR (125.7 MHz, $CDCl_3$) Compound S12.

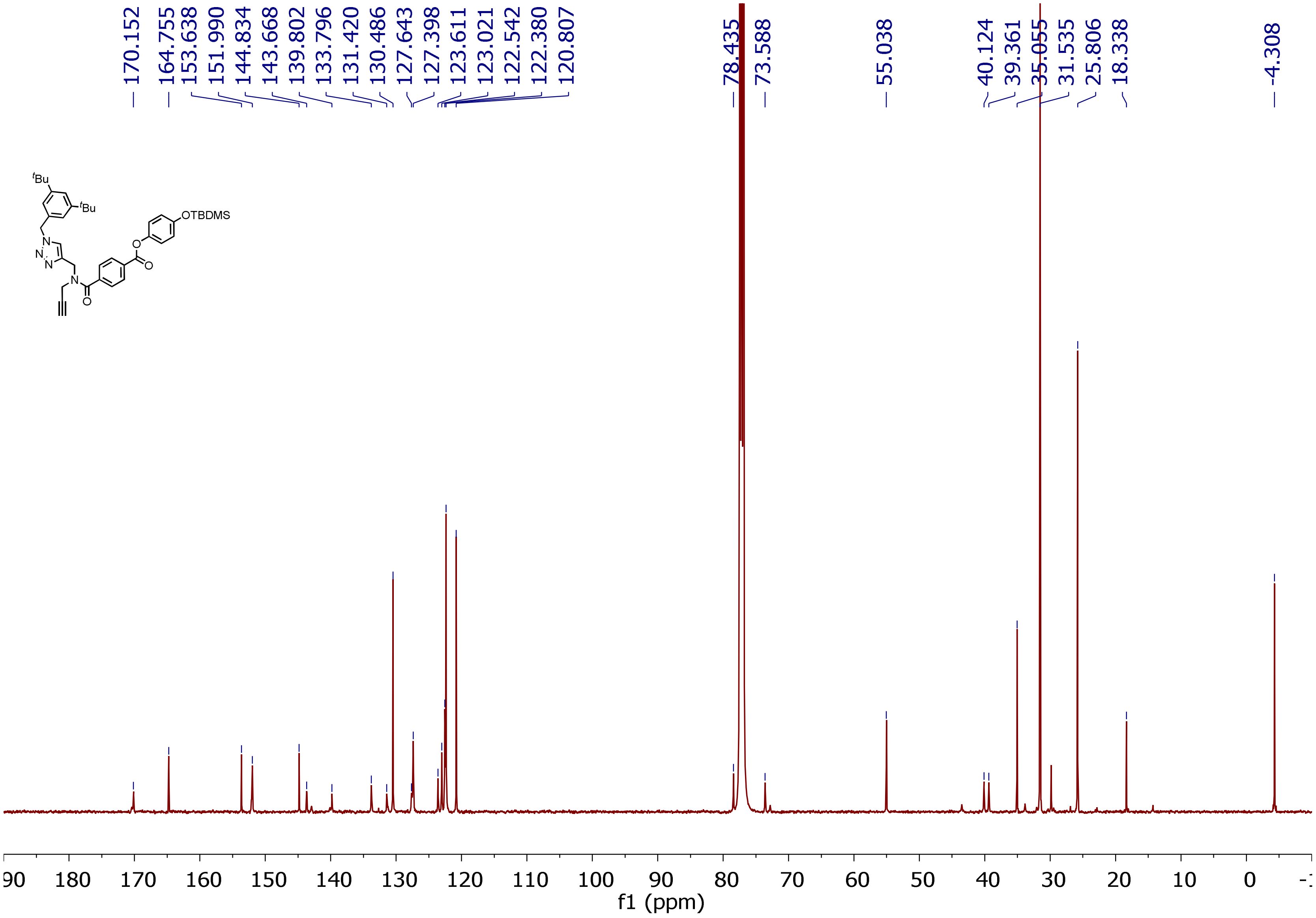

Compound S13.

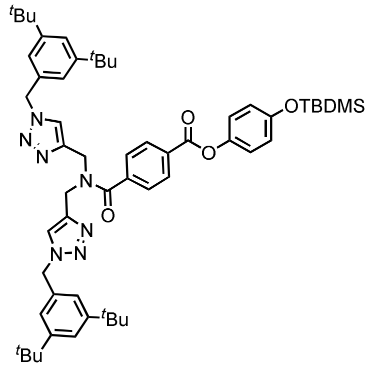

^{1}H NMR (400 MHz, CDCl$_3$): δ_H = 8.18 (d, 2H, *J* = 8.0 Hz), 7.74-765 (m, 4H), 7.43 (s, 1H), 7.12 (s, 4H), 7.07 (d, 2H, *J* = 9.0 Hz), 6.86 (d, 2H, *J* = 9.0 Hz), 5.50 (s, 4H), 4.97-458 (bs, 4H, rotamers), 1.30 (s, 36H), 0.99 (s, 9H), 0.21 (s, 6H).

^{13}C NMR (125.7 MHz, CDCl$_3$): δ_C = 170.8, 164.8, 153.6, 152.1, 144.9, 140.3, 133.7, 131.2, 130.5, 127.9, 127.4, 124.0, 123.1, 122.6, 122.4, 120.8, 55.1, 43.7 and 39.2 (rotamers), 35.0, 31.5, 25.8, 18.3, -4.3.

HRMS (ES+): calcd for $C_{56}H_{76}N_7O_4Si$ 938.5728 $[M+H]^+$, found 938.5726 $[M+H]^+$.

FT-IR (ATR): ν_{max} 2957, 2932, 2862, 1740, 1640, 1502, 1258, 1192, 842 and 775 cm^{-1}.

^{1}H-NMR (400 MHz, $CDCl_3$) Compound S13.

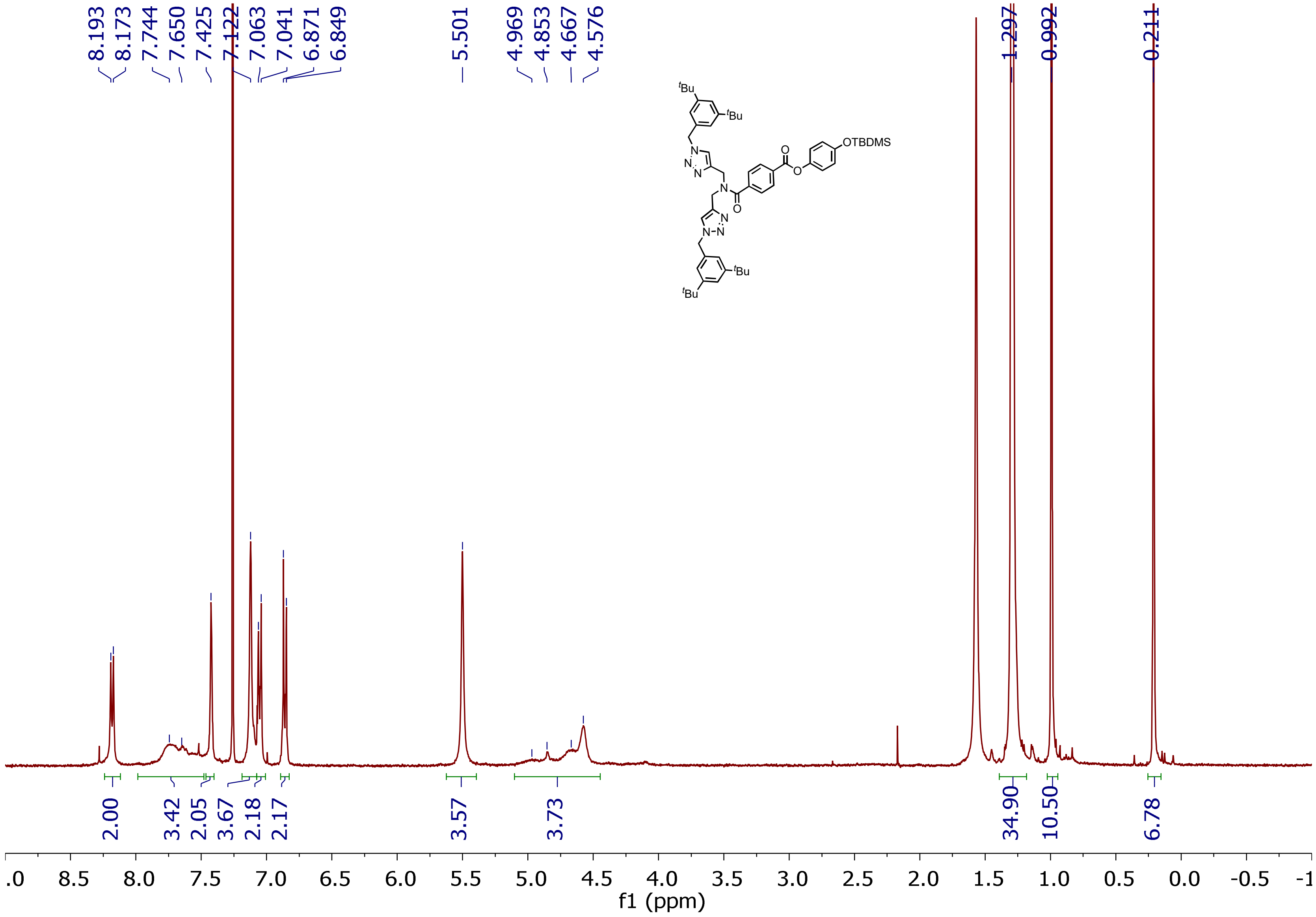

^{13}C-NMR (125.7 MHz, CDCl$_3$) Compound S13.

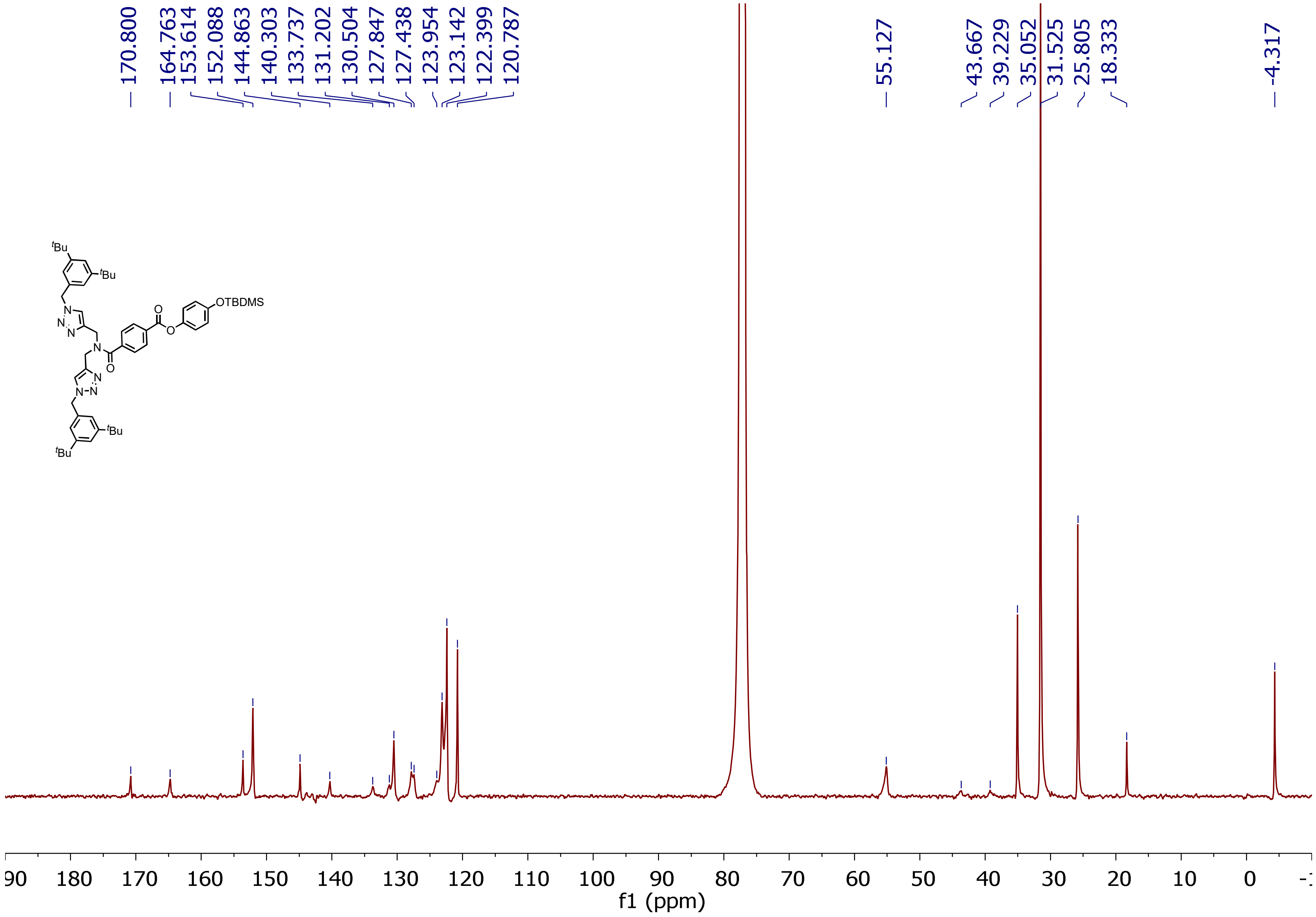

Compound S14.

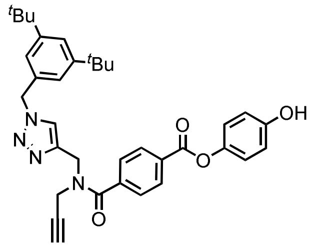

A solution of **S12** (0.137 g, 0.19 mmol) in dry THF (5 mL) was treated at 0 °C with TBAF solution (1M in THF, 0.193 mL, 0.19 mmol). The reaction was stirred for 5 minutes and quenched with 5% soln. HCl and extracted with EtOAc (3x) followed by washing with H_2O and brine The organic layer was dried over anhydrous $MgSO_4$ and concentrate under vacuum. The crude was repetitively dissolved in $CHCl_3$ and evaporated, and dried under high vacuum to remove traces of TBDMS-F. Compound **S14** was obtained as a foam (0.111 g, quantitative).

^{1}H NMR (400 MHz, $CDCl_3$): δ_H = 8.22 (d, 2H, *J* = 8.0 Hz), 7.71 (s partially overlapped, 1H), 7.64 (m, 2H), 7.44 (t, 1H, *J* = 1.5 Hz), 7.11 (s, 2H), 7.06 (d, 2H, *J* = 9.0 Hz), 6.86 (d, 2H, *J* = 9.0 Hz), 5.52 (s, 2H), 4.87, 4.65, 4.36 and 4.09 (bs, 4H, rotamers), 2.34 and 2.23 (bs, 1H, rotamers), 1.31 (s, 18H).

^{13}C NMR (125.7 MHz, $CDCl_3$): δ_C = 170.2, 164.9, 153.7, 152.0, 144.4, 143.6, 139.8, 133.8, 131.4, 130.5, 127.4, 123.6, 123.0, 122.7, 122.6, 116.2, 78.4, 73.6, 55.1, 40.2 and 39.4 (rotamers), 35.1, 31.5.

HRMS (ES+): calcd for $C_{35}H_{39}N_4O_4$ 579.2971 $[M+H]^+$, found 579.2973 $[M+H]^+$.

FT-IR (ATR): ν_{max} 2958, 1735, 1640, 1600, 1506, 1457, 1264, 1192, 1077 cm^{-1}.

^{1}H-NMR (400 MHz, $CDCl_3$) Compound S14.

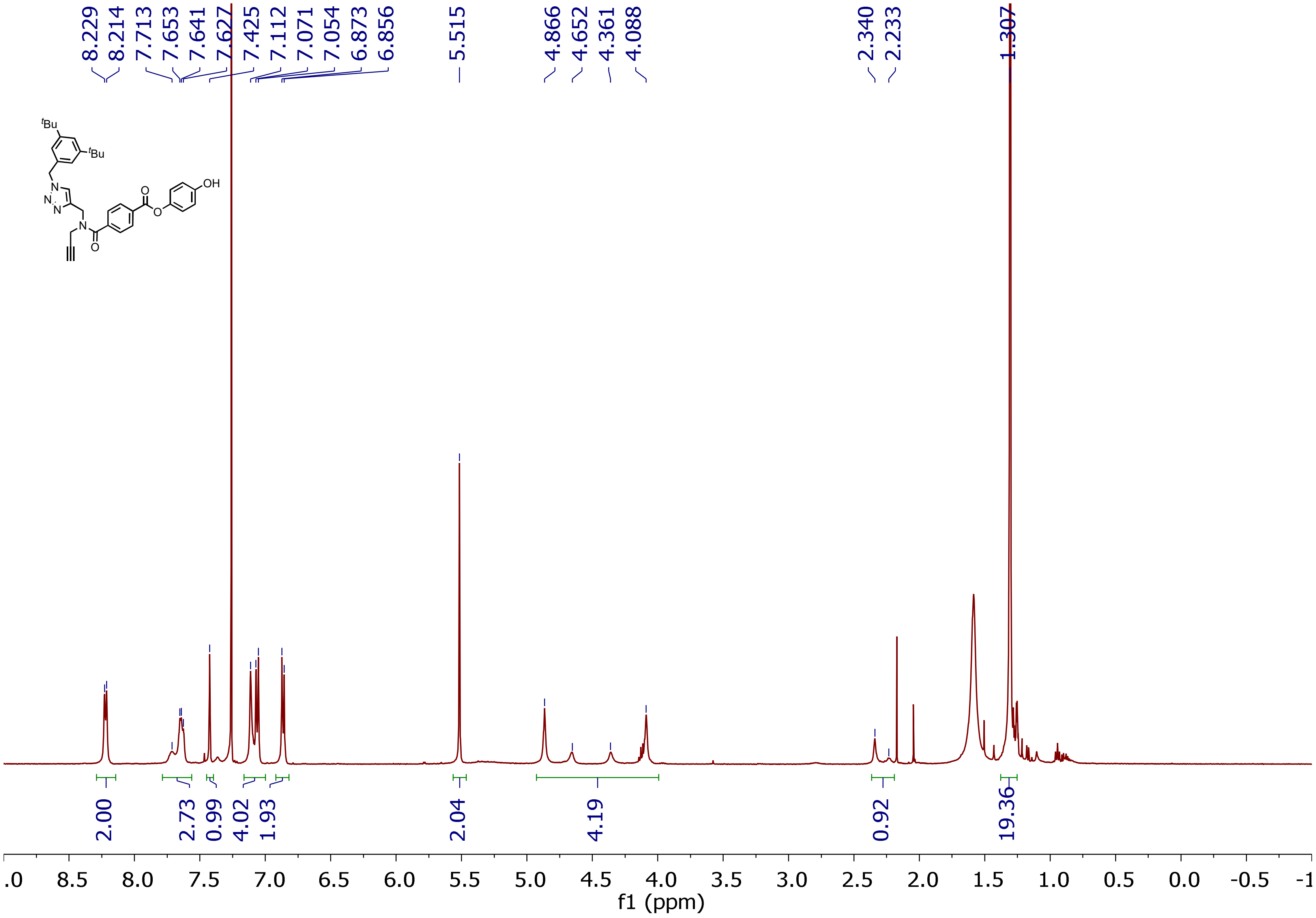

^{13}C-NMR (125.7 MHz, $CDCl_3$) Compound S14.

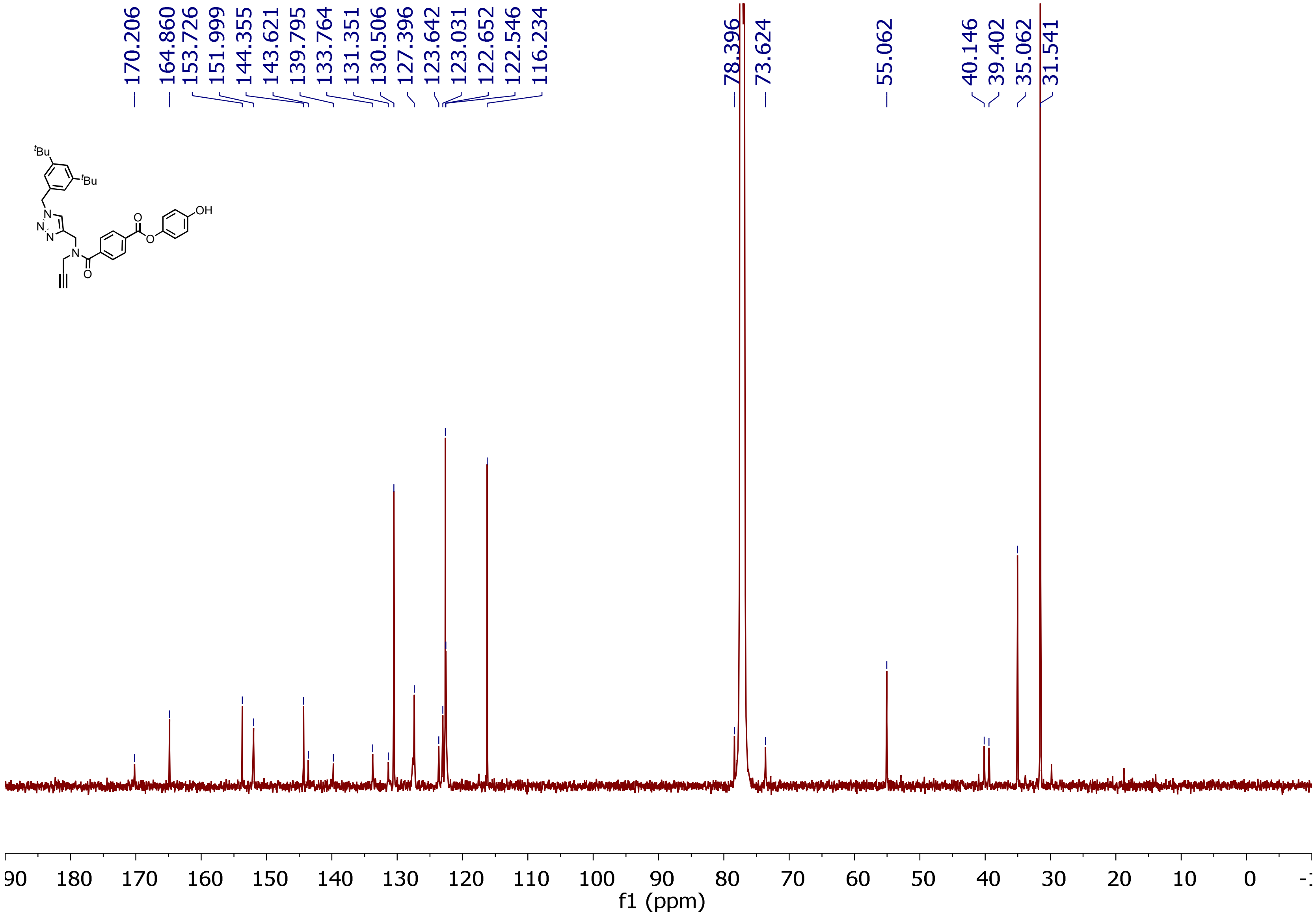

Compound S15.

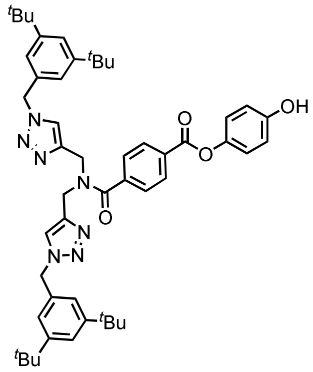

A solution of **S13** (0.249 g, 0.27 mmol) in dry THF (5 mL) was treated at 0 ^{o}C with TBAF solution (1M in THF, 0.266 mL, 0.27 mmol). The reaction was stirred for 5 minutes and quenched with 5% soln. HCl and extracted with EtOAc (3x) followed by washing with H_2O and brine The organic layer was dried over anhydrous $MgSO_4$ and concentrate under vacuum. The crude material was purified by flash column chromatography on silica gel (gradient from 0% to 3%of MeOH in CH_2Cl_2) to afford compound **S15** (0.191 g, 87 %) as a white solid.

Melting point: 111-113 ^{o}C.

^{1}H NMR (400 MHz, CDCl$_3$): δ_H = 8.16 (d, 2H, *J* = 8.5 Hz), 7.92 (bs, 1H), 7.76 (d, 2H, *J* = 8.5 Hz), 7.75 and 7.73 (s, 2H, rotamers), 7.43 (t, 2H, *J* = 2 Hz), 7.12 (d, 4H, *J* = 2 Hz), 6.99 (d, 2H, *J* = 9.0 Hz), 6.83 (d, 2H, *J* = 9.0 Hz), 5.52 and 5.51 (bs, 4H, rotamers), 4.68 and 4.57 (bs, 4H, rotamers), 1.31 (s, 38H).

^{13}C NMR (100.6 MHz, CDCl$_3$): δ_C = 171.0, 164.3, 154.6, 152.0, 143.8, 140.0, 133.6, 131.1, 130.4, 127.9, 124.1, 123.4, 123.0, 122.5, 122.4, 116.3, 55.1, 44.0 and 39.4 (rotamers), 35.0, 31.5.

HRMS (ES+): calcd for $C_{50}H_{62}N_7O_4$ 824.4863 $[M+H]^+$, found 824.4866 $[M+H]^+$.

FT-IR (ATR): ν_{max} 3300, 2963, 17341632, 1263, 1248, 1191, 1076 and 754 cm^{-1}.

^{1}H-NMR (400 MHz, CDCl$_3$) Compound S15.

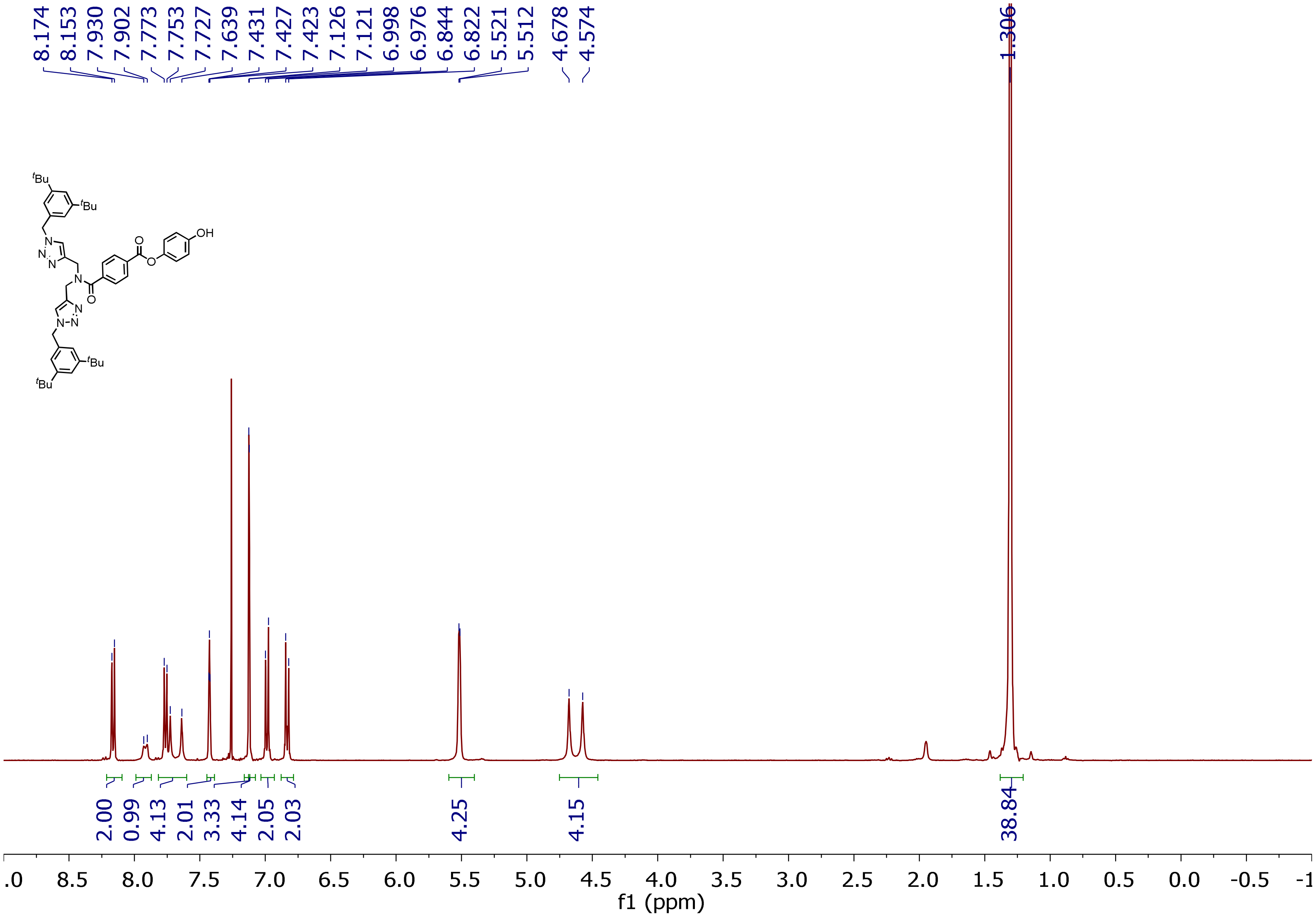

^{13}C-NMR (100.6 MHz, CDCl$_3$) Compound S15.

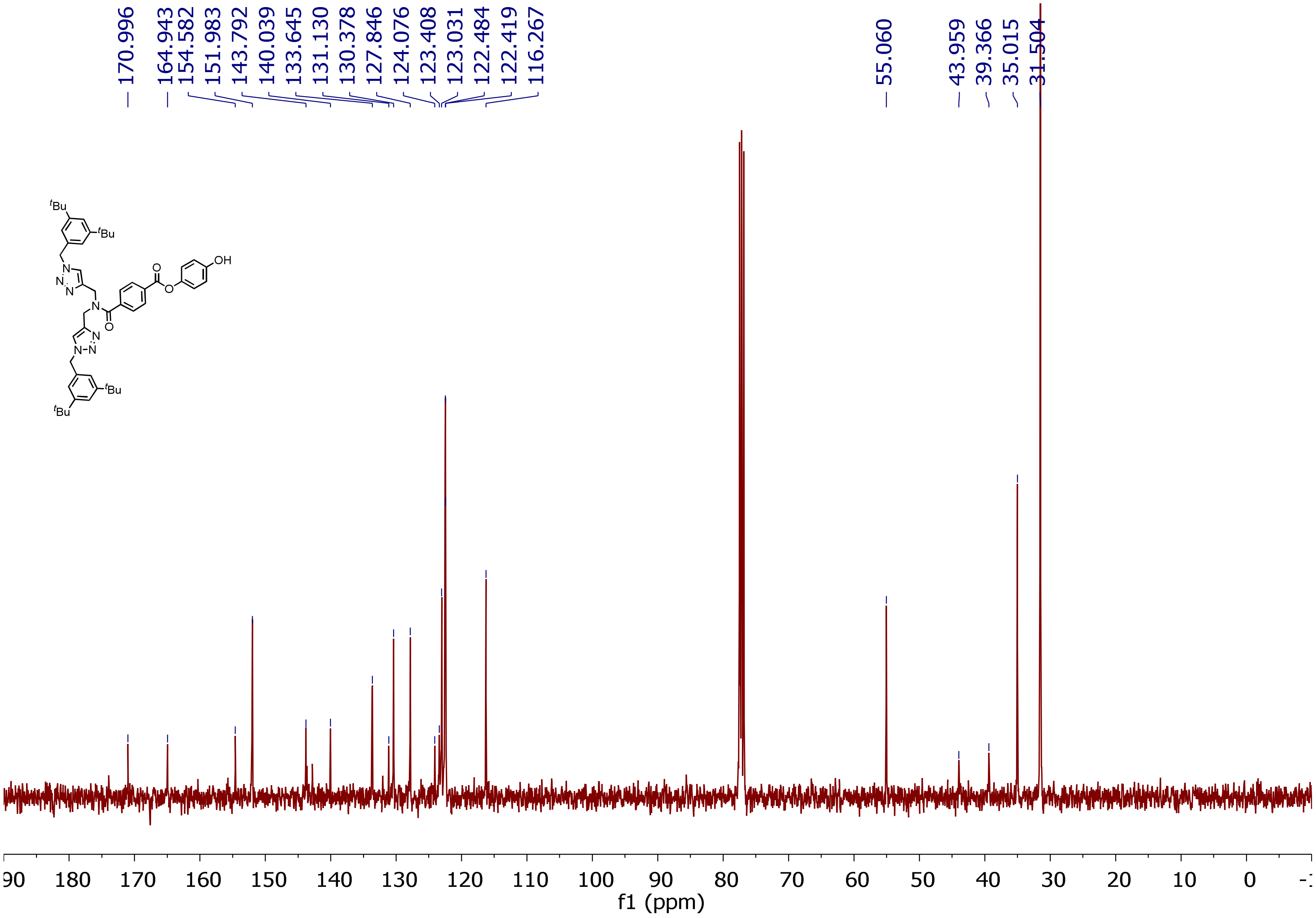

Compound S16.

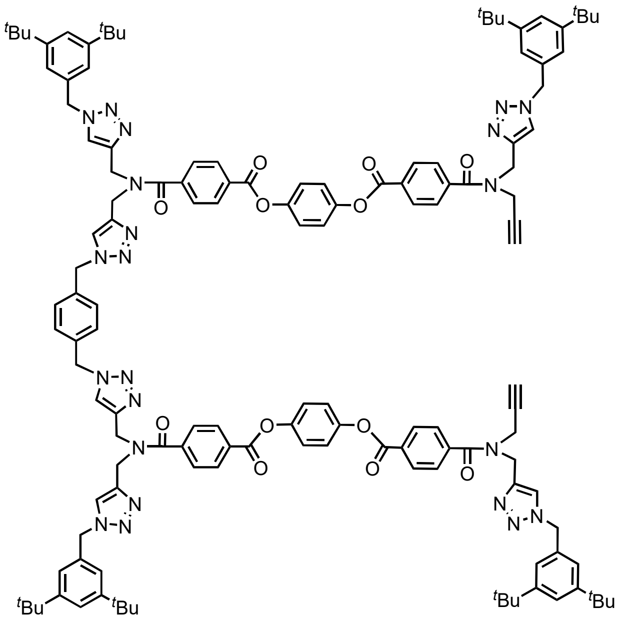

Compound **S10** (0.174 g, 0.15 mmol), compound **S14** (0.196 g, 0.33 mmol), EDC (0.069 g, 0.36 mmol) and DMAP (0.004 g, 0.03 mmol) were dissolved under N_2 atmosphere in dry CH_2Cl_2 (4 ml). The solution was stirred overnight under N_2 atmosphere at room temperature. The crude was diluted with EtOAc (5 mL) and washed with 5% aq. soln. HCl (3x), H_2O (1x) and brine. The organic phase was dried with $MgSO_4$ and concentrated *in vacuo*. The crude material was purified by flash column chromatography on silica gel (gradient from 0% to 4% of MeOH in CH_2Cl_2) to afford compound **S16** (0.283 g, 83 %) as a white solid.

Melting point: 124-126 ^oC.

^{1}H NMR (400 MHz, $CDCl_3$): δ_H = 8.23 (m, 8H), 7.80 (d, 4H, *J* = 8.0 Hz), 7.77-7-64 (m, 8H), 7.63 and 7.56 (s, 2H, rotamers), 7.42 (m, 4H), 7.31 (s, 4H), 7.28 (s, 8H), 7.11 (s, 8H), 5.52 (m, 12H), 4.87, 4.67 and 4.56 (bs, 12H, rotamers), 4.36 and 4.09 (bs, 4H), 2.35 and 2.24 (bs, rotamers), 1.31 (s, 36H), 1.30 (s, 36H).

^{13}C NMR (125.7 MHz, $CDCl_3$): δ_C = 170.7, 170.1, 164.5, 164.4, 152.0, 148.5, 148.4, 144.1, 143.8, 143.6, 143.3, 143.0, 140.6, 140.0, 135.4, 133.7, 131.0, 130.7, 130.6, 130.5, 129.0, 129.0, 128.0, 127.7, 127.5, 123.9, 123.6, 123.4, 123.0, 122.8, 122.8, 122.5, 78.4, 73.6, 55.0, 53.8, 44.2, 43.9, 40.1, 39.6 and 39.4 (rotamers), 35.0, 31.5.

HRMS (ES+): calcd for $C_{136}H_{149}N_{22}O_{12}$ 2282.1725 $[M+H]^+$, found 2282.1733 $[M+H]^+$.

FT-IR (ATR): ν_{max} 2963, 2867, 1736, 1635, 1259, 1249, 1172, 1072 and 751 cm^{-1}.

^{1}H-NMR (400 MHz, $CDCl_3$) Compound S16.

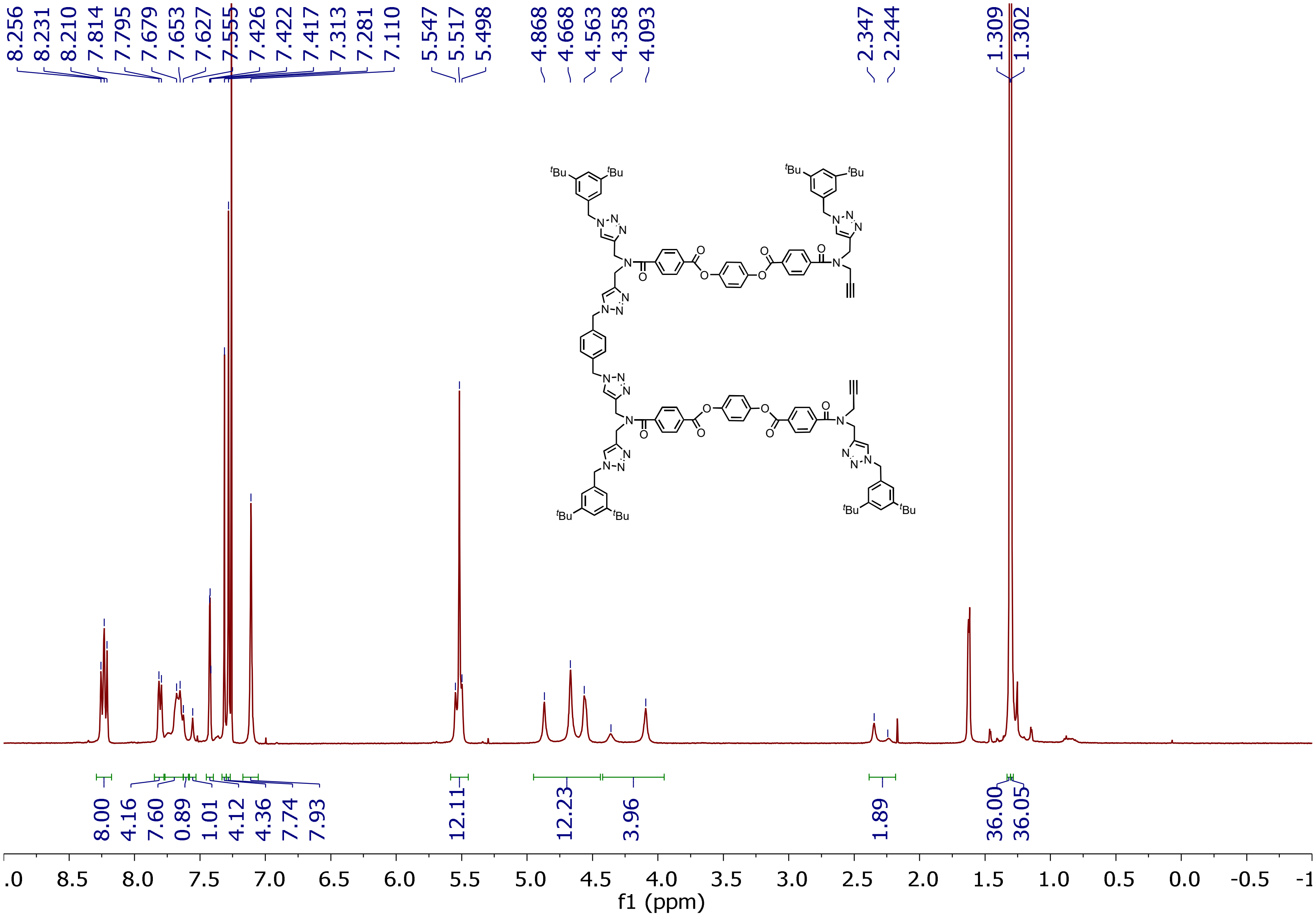

^{13}C-NMR (125.7 MHz, $CDCl_3$) Compound S16.

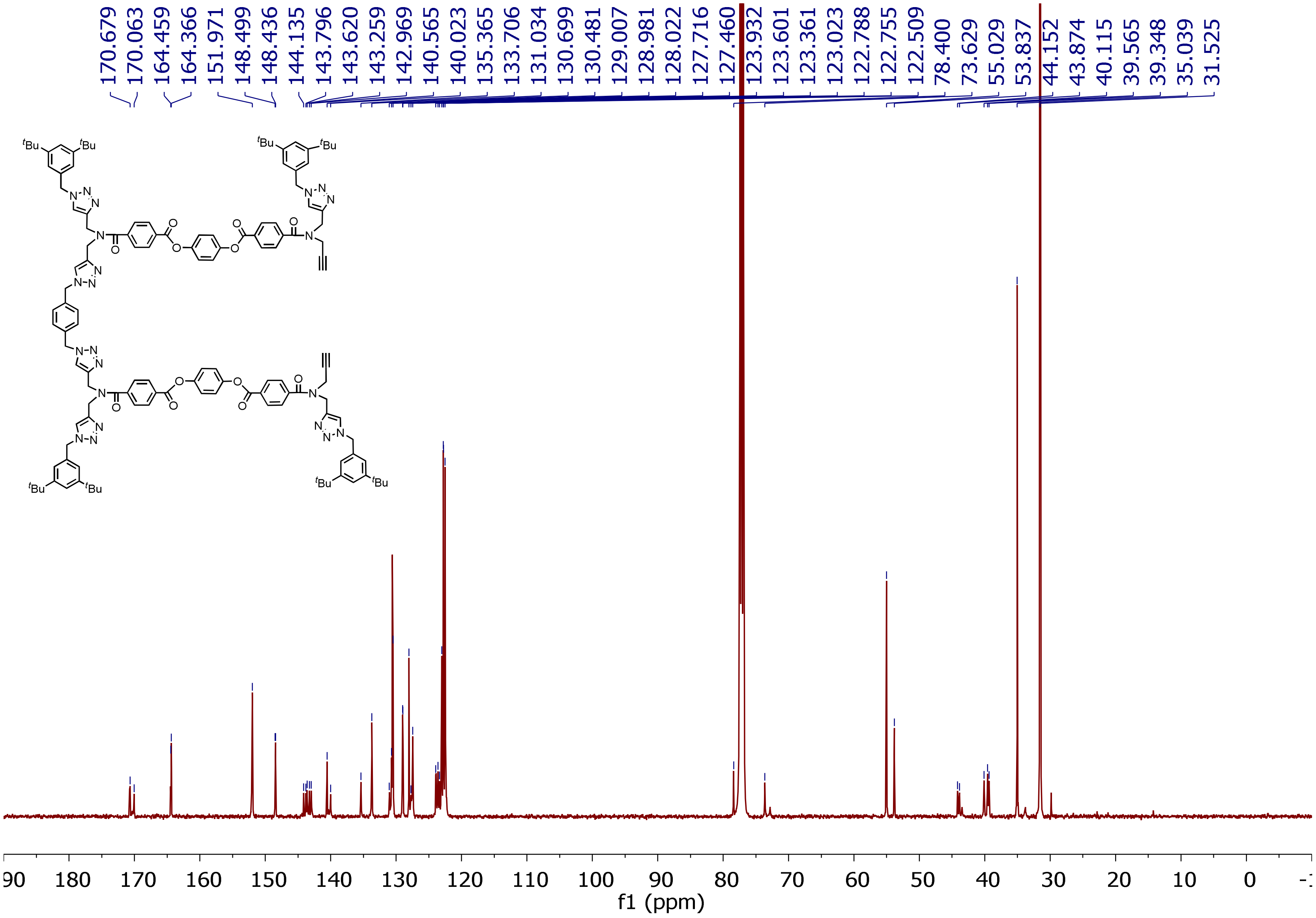

Compound 1.

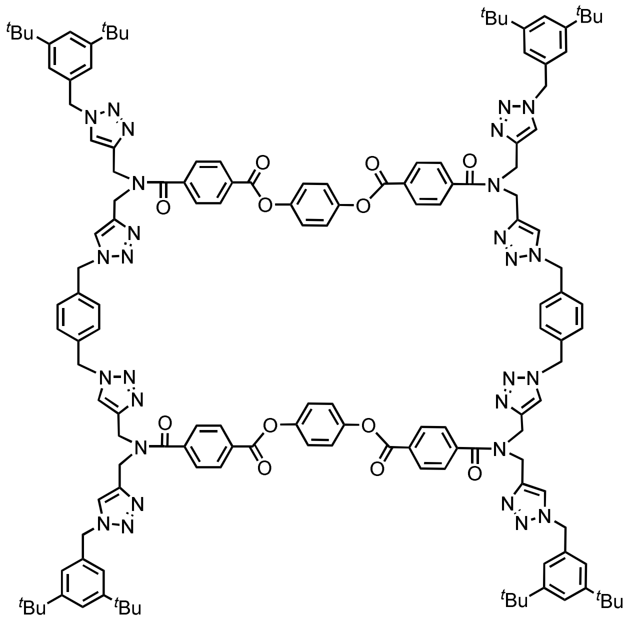

Compound **S16** (14 mg, 0.062 mmol), $Cu(CH_3CN)_4PF_6$ (0.6 mg, 0.015 mmol) and TBTA (0.8 g, 0.01 mmol) were mixed in a round-bottom flask and, under N_2, THF (25 mL) was added. A solution of 1,4-bis(azidomethyl)benzene **S9** (1.2 mg, 0.062 mmol) in THF (0.5 mL) was added and the reaction was stirred at room temperature for 48h. The solvent was evaporated to dryness and the crude was purified by flash column chromatography on silica gel (gradient from 0% to 6% of MeOH in CH_2Cl_2) to afford **1** (9 mg, 66%) as a white solid.

Melting point: 151-153 ^{o}C.

^{1}H NMR (400 MHz, $CDCl_3$): δ_H = 8.20 (m, 8H), 7.77 (m, 8H), 7.68-7.51 (m, 8H), 7.42 (s, 4H), 7.30 (m, 8H), 7.23 (m, 8H), 7.11 (s, 8H), 5.54-5.50 (m, 16 H), 4.67 and 4.52 (bs, 16H), 1.30 (s, 72H).

^{13}C NMR (125.7 MHz, $CDCl_3$): δ_C = 170.7, 164.5, 152.0, 148.5, 143.8, 143.0, 140.6, 135.4, 133.7, 130.7, 130.5, 129.3, 129.2, 128.9, 128.4, 128.15, 128.0, 123.9, 123.0, 122.8, 122.5, 55.0, 53.8, 44.2, 43.9, 39.7 and 39.6 (rotamers), 35.1, 31.5.

HRMS (ES+): calcd for $C_{144}H_{157}N_{28}O_{12}$ 2470.2536 $[M+H]^+$, found 2470.2471 $[M+H]^+$.

FT-IR (ATR): ν_{max} 2962, 2925, 1738, 1635, 1261, 1174, 1073 and 772 cm^{-1}.

^{1}H-NMR (400 MHz, $CDCl_3$) Compound 1.

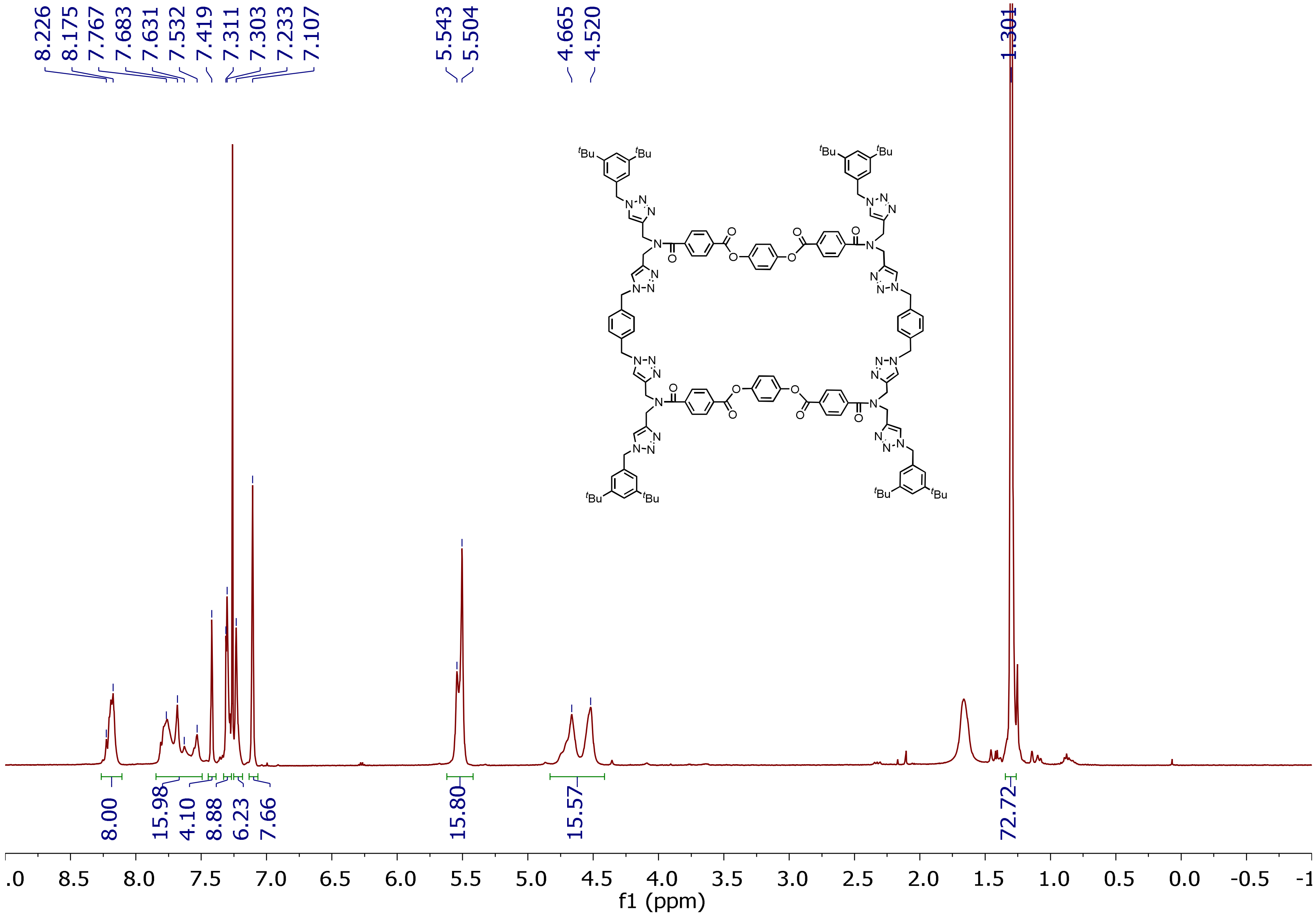

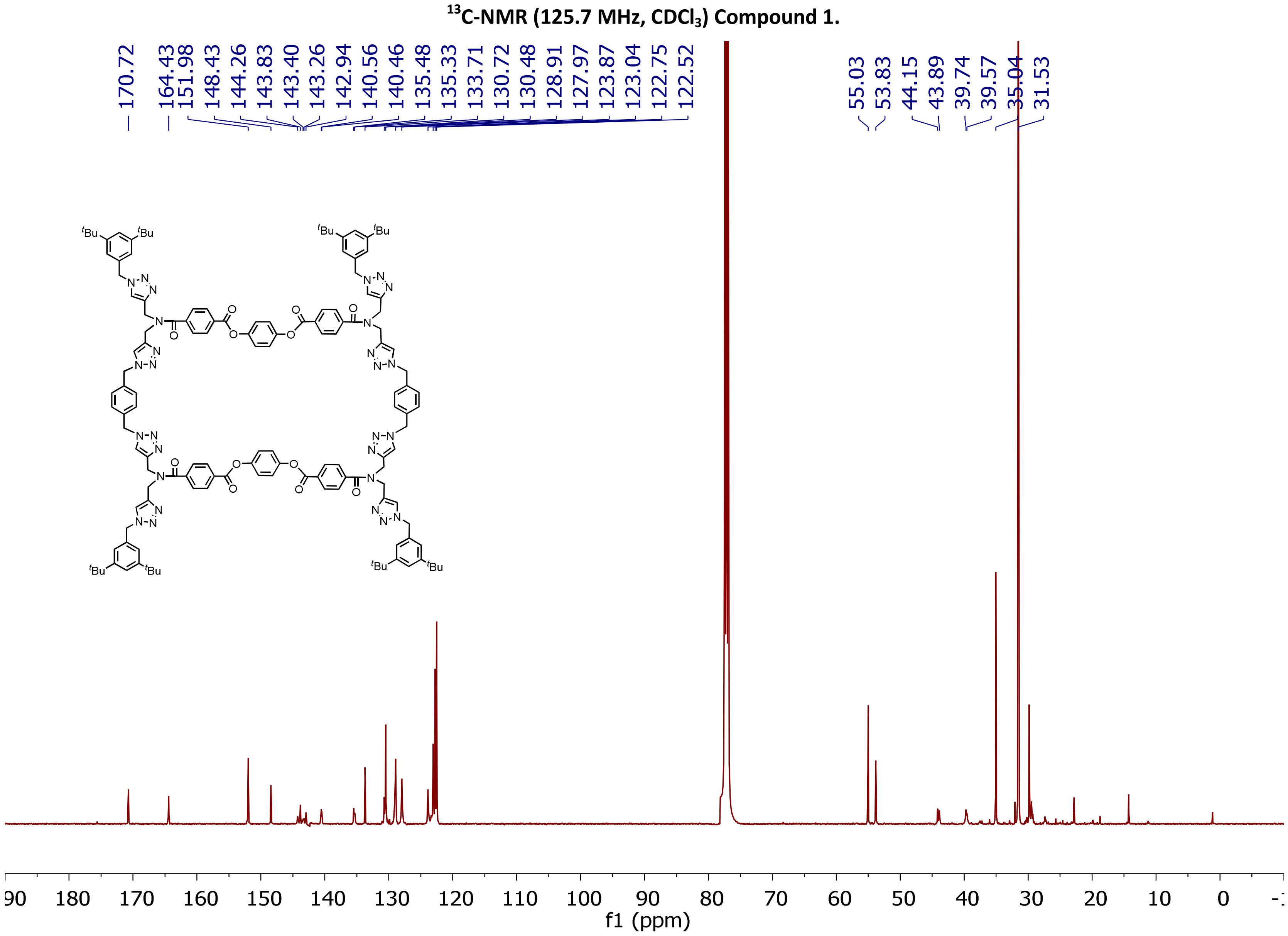

^{13}C-NMR (125.7 MHz, CDCl$_3$) Compound 1.
170.72
164.43
151.98
148.43
144.26
143.83
143.40
143.26
142.94
140.56
140.46
135.48
135.33
133.71
130.72
130.48
128.91
127.97
123.87
123.04
122.75
122.52
55.03
53.83
44.15
43.89
39.74
39.57
35.04
31.53
f1 (ppm)

Compound 2.

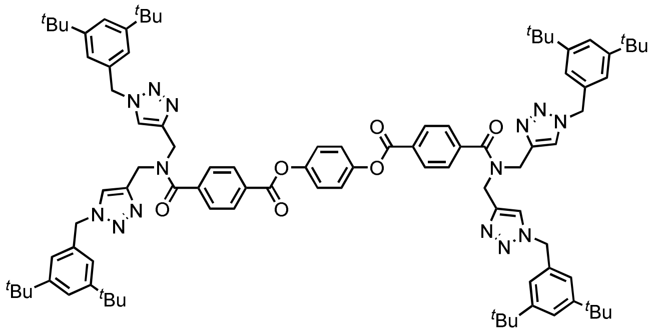

Compound **S11** (0.104 g, 0.14 mmol), compound **S15** (0.124 g, 0.15 mmol), EDC (0.030 g, 0.16 mmol) and DMAP (0.002 g, 0.01 mmol) were dissolved under N_2 atmosphere in dry CH_2Cl_2 (4 ml). The solution was stirred under N_2 atmosphere at room temperature for 1h. The crude was diluted with EtOAc (5 mL) and washed with 5% aq. soln. HCl (3x), H_2O (1x) and brine. The organic phase was dried with $MgSO_4$ and concentrated *in vacuo*. The crude material was purified by flash column chromatography on silica gel (gradient from 25% to 90%of EtOAc in Pet. Ether) to afford compound **2** (0.171, 79 %) as a white solid.

Melting point: 102-104 ^{o}C.

^{1}H NMR (400 MHz, CDCl$_3$): δ_H = 8.22 (d, 4H, *J* = 8.5 Hz), 7.81 (d, 4H, *J* = 8.5 Hz), 7.69 and 7.60 (s, 4H, rotamers), 7.28 (s, 4H), 7.42 (t, 4H, *J* = 2 Hz), 7.11 (s, 8H), 5.53 and 5.50 (bs, 4H, rotamers), 4.67 and 4.56 (bs, 8H, rotamers), 1.31 (s, 72H).

^{13}C NMR (100.6 MHz, CDCl$_3$): δ_C = 170.6, 164.5, 152.0, 148.5, 143.8 and 143.0 (rotamers), 140.6, 133.8, 130.7, 130.5, 128.0, 123.8, 123.0, 122.7, 122.5, 55.0, 43.9 and 39.3 (rotamers), 35.0, 31.5.

HRMS (ES+): calcd for $C_{94}H_{117}N_{14}O_6$ 1537.9281 $[M+H]^+$, found 1537.9261 $[M+H]^+$.

FT-IR (ATR): ν_{max} 2961, 2905, 2868, 1739, 1635, 1260, 1249, 1173, 1072 and 756 cm^{-1}.

^{1}H-NMR (400 MHz, $CDCl_3$) Compound 2.

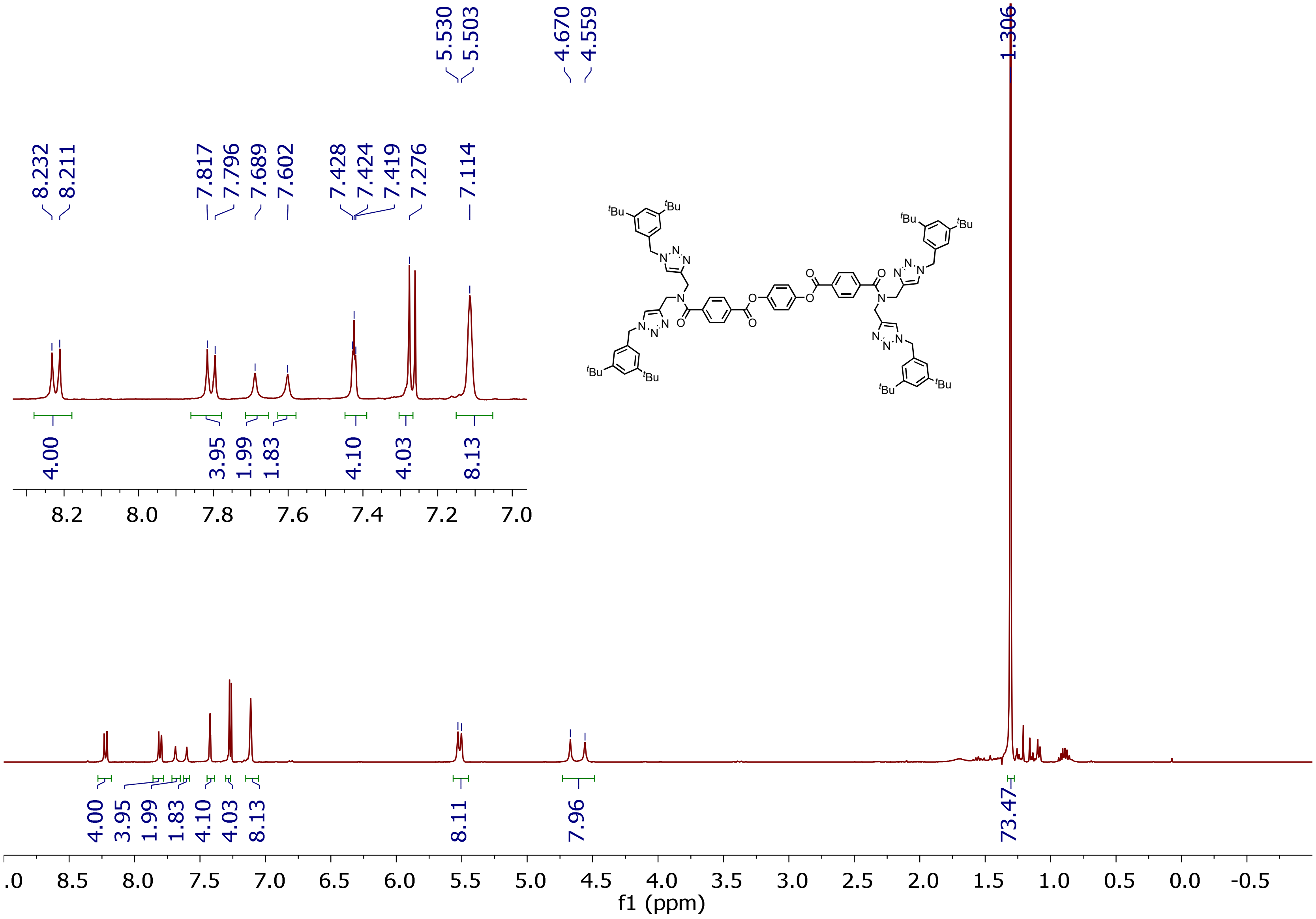

^{13}C-NMR (100.6 MHz, CDCl$_3$) Compound 2.

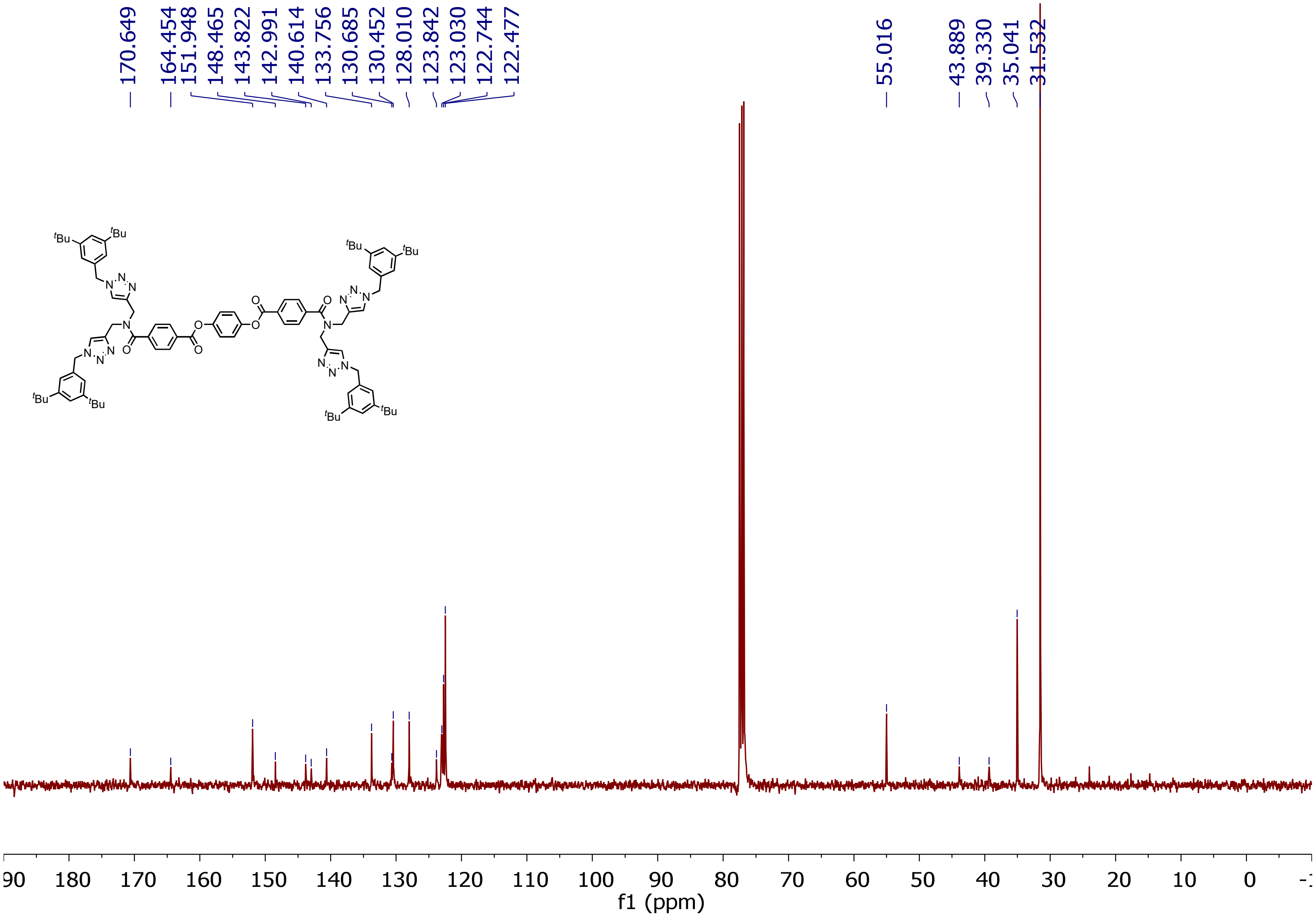

Methyl 4-(diisopropylcarbamoyl)benzoate (S18).

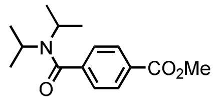

Methyl 4-chlorocarbonylbenzoate **S17** (0.500g, 2.52 mmol) was dissolved in dry CH_2Cl_2 (3 mL9 under N_2 atmosphere and the solution added to a solution of diisopropylamine (0.856 mL, 6.11 mmol) in dry CH_2Cl_2 (7 mL). The reaction was stirred at room temperature for 2h. The solvent was evaporated to dryness and the crude dissolved in EtOAc and washed with 5% aq. soln. HCl (2x), sat. soln. $NaHCO_3$ (1x), 2N NaOH (1x), sat. soln. $NaHCO_3$ (1x), H_2O (1x) and brine. The organic phase was dried with $MgSO_4$ and concentrated *in vacuo*. The crude material was purified by flash column chromatography on silica gel (gradient from 5% to 50%of EtOAc in Pet. Ether) to afford **S18** (0.607 g, quantitative) as a white solid.

Melting point: 83-84 ^{o}C.

^{1}H NMR (400 MHz, $CDCl_3$): δ_H = 8.05 (d, 2H, *J* = 8.5 Hz), 7.36 (d, 2H, *J* = 8.5 Hz), 3.92 (s, 3H), 3.72 and 3.54 (bs, 2H, rotamers), 1.54 and 1.12 (bs, 12H, rotamers).

^{13}C NMR (125.7 MHz, $CDCl_3$): δ_C = 170.1, 166.7, 143.3, 130.4, 130.1, 125.7, 52.4, 51.1 and 46.1 (rotamers), 20.8.

HRMS (ES+): calcd for $C_{15}H_{22}NO_3$ 264.1600 $[M+H]^+$, found 264.1601 $[M+H]^+$.

FT-IR (ATR): ν_{max} 2968, 2932, 1724, 1632, 1438, 1340, 1276, 1107 and 717 cm^{-1}.

^{1}H-NMR (400 MHz, $CDCl_3$) Methyl 4-(diisopropylcarbamoyl)benzoate (S18).

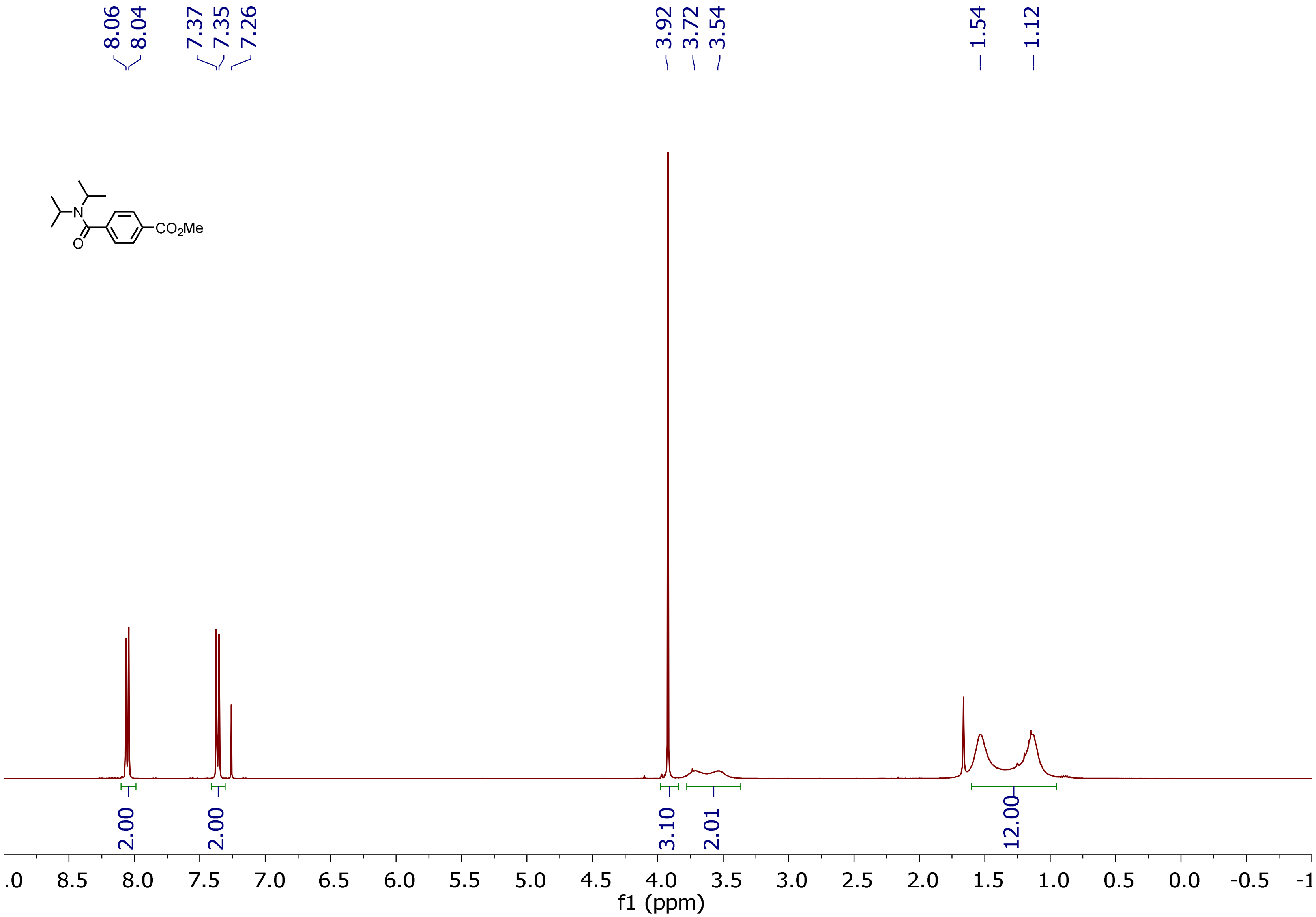

^{13}C-NMR (100.6 MHz, $CDCl_3$) Methyl 4-(diisopropylcarbamoyl)benzoate (S18).

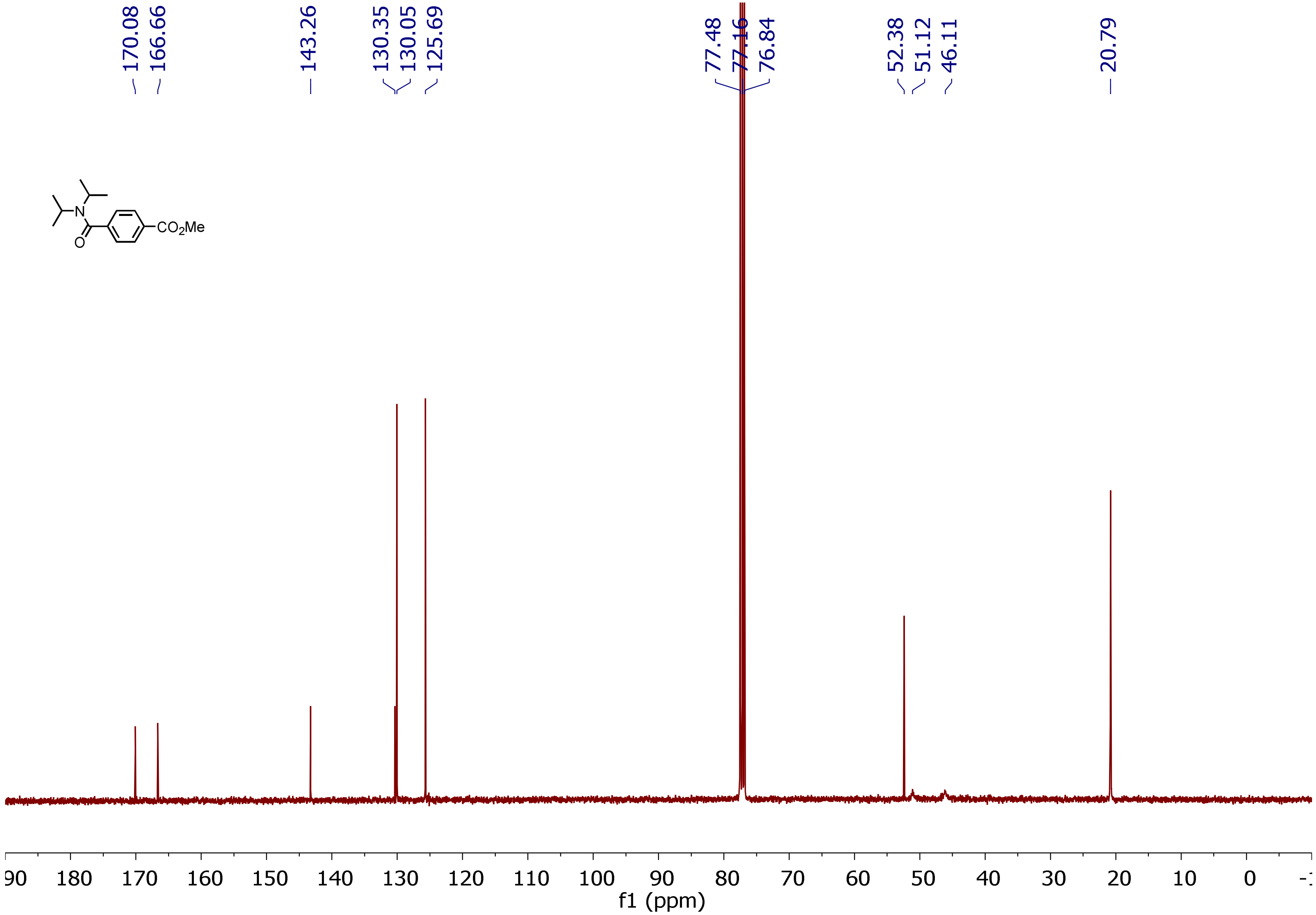

Compound 3.

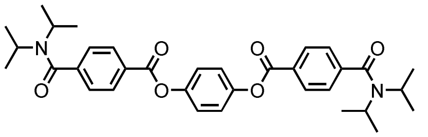

Compound **S18** (0.585 g, 2.49 mmol) was dissolved in MeOH (10 mL) and 2N NaOH solution (3.75 mL, 7.46 mmol) was added. After stirring at room temperature for 16 h, the solvent was removed under vacuum and the crude dissolved in H_2O and acidified to pH 2-3 with 0.1 N HCl soln.. The slurry was extracted with EtOAc (3x) and the combined organic phase was washed with brine, dried with $MgSO_4$ and concentrated, yielding the corresponding carboxylic acid derivative (0.450 g), which was used without further purification. A solution of the carboxylic acid (0.450 g, 2.03 mmol) in DMF (4 mL) was treated with hydroquinone (0.112 g, 1.02 mmol), EDC (0.468 g, 2.44 mmol) and DMAP (0.013 g, 0.10 mmol). After 3 days of stirring at room temperature, the reaction was diluted with EtOAc and washed with 0.1 N HCl soln. (1x), 5% LiCl soln. (2x) and brine. The organic phase dried with $MgSO_4$ and concentrated. The resulting crude was purified by flash column chromatography on silica gel (gradient from 0% to 50%of EtOAc in Pet. Ether) to afford **3** (0.463 g, 65% over 2 steps) as a white solid.

Melting point: 259-260 ^{o}C.

^{1}H NMR (400 MHz, CDCl$_3$): δ_H = 8.23 (d, 2H, *J* = 8.0 Hz), 7.45 (d, 2H, *J* = 8.0 Hz), 7.29 (s, 4H), 3.74 and 3.55 (bs, 4H, rotamers), 1.56 and 1.16 (bs, 24H, rotamers).

^{13}C NMR (125.7 MHz, CDCl$_3$): δ_C = 169.9, 164.6, 148.5, 144.0, 130.7, 129.5, 125.9, 122.8, 51.1 and 46.1 (rotamers), 20.8.

HRMS (ES+): calcd for $C_{34}H_{41}N_2O_6$ 573.2965 $[M+H]^+$, found 573.2949 $[M+H]^+$.

FT-IR (ATR): ν_{max} 2971, 1740, 1342, 1261, 1172, 1071, 1015 and 755 cm^{-1}.

^{1}H-NMR (400 MHz, $CDCl_3$) Compound 3.

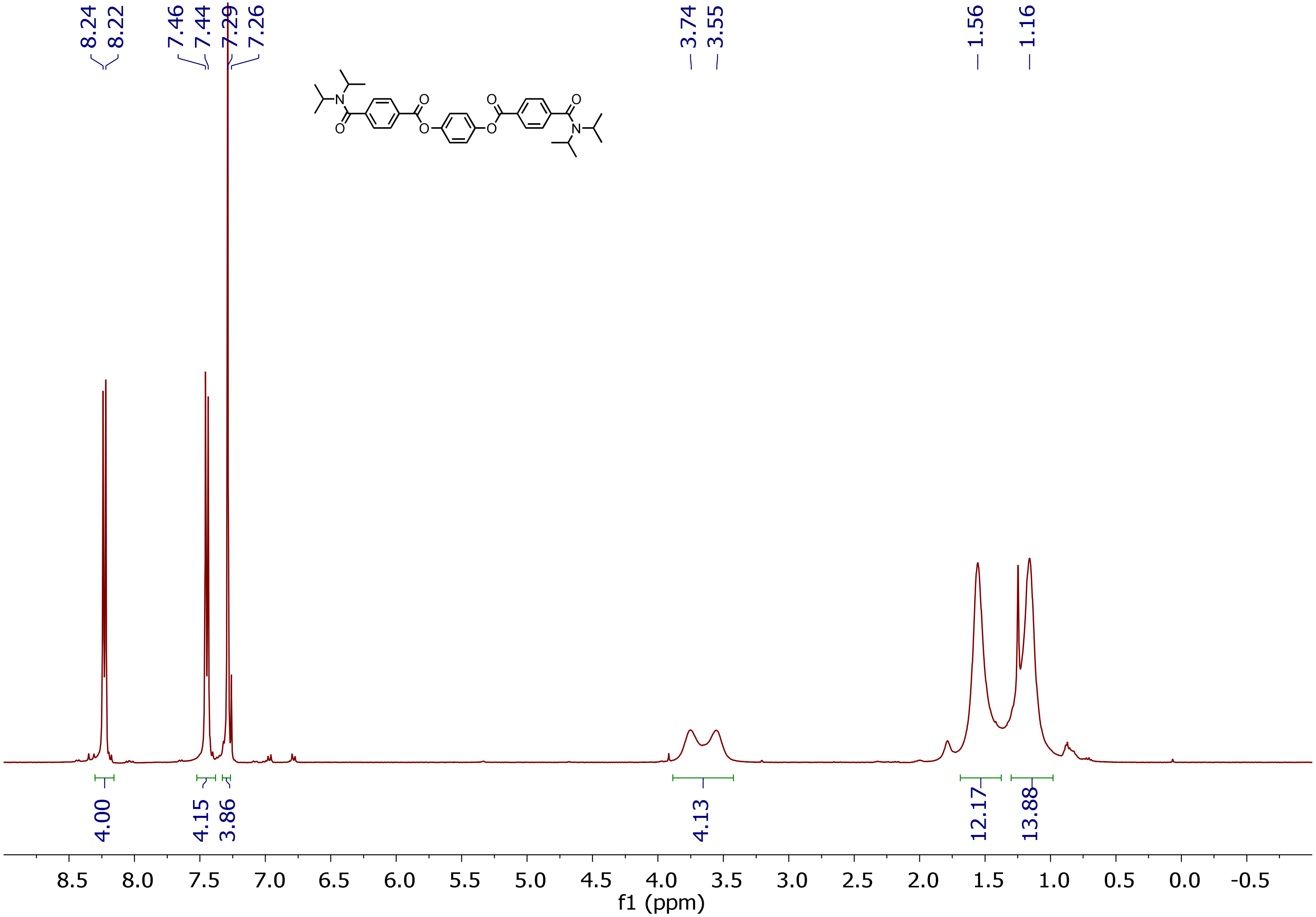

^{13}C-NMR (100.6 MHz, CDCl$_3$) Compound 3.

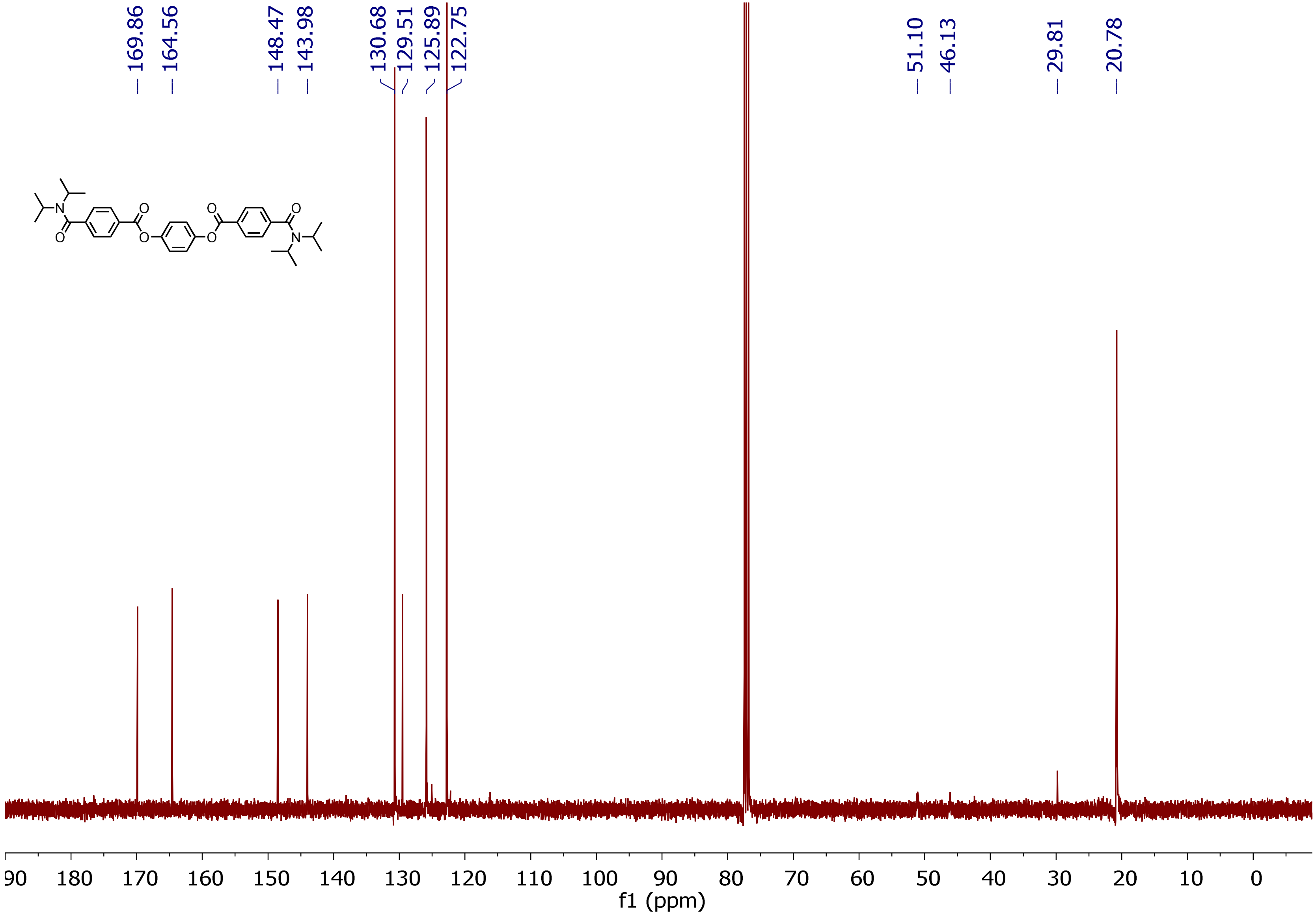

References

[S1] A. Takahashi, M. Sakai and T. Kato. *Polym. J.* **1980**, *12*, 335.

[S2] Schrödinger Release 2016-4: MacroModel, Schrödinger, LLC, New York, NY, 2016.

[S3] The PyMOL Molecular Graphics System, Version 1.6 Schrödinger, LLC.

[S4] J. J. Gassensmith, L. Barr, J. M. Baumes, A. Paek, A. Nguyen and B. D. Smith. *Org. Lett.* **2008**, *10*, 3343.

[S5] J. R. Thomas, X. Liu and P. J. Hergenrother. *J. Am. Chem. Soc.* **2005**, *127*, 12434.
